# Adherence to the Atrial fibrillation Better Care pathway and the risk of adverse health outcomes in older care home residents with atrial fibrillation: a retrospective data linkage study 2003–18

**DOI:** 10.1093/ageing/afae021

**Published:** 2024-02-22

**Authors:** Leona A Ritchie, Stephanie L Harrison, Peter E Penson, Ashley Akbari, Fatemeh Torabi, Joe Hollinghurst, Daniel Harris, Oluwakayode B Oke, Asangaedem Akpan, Julian P Halcox, Sarah E Rodgers, Gregory Y H Lip, Deirdre A Lane

**Affiliations:** Liverpool Centre for Cardiovascular Science, University of Liverpool, Liverpool John Moores University and Liverpool Heart and Chest Hospital, Liverpool, UK; Department of Cardiovascular and Metabolic Medicine, Institute of Life Course and Medical Sciences, University of Liverpool, Liverpool L7 8TX, UK; School of Pharmacy and Biomolecular Sciences, Liverpool John Moores University, Liverpool L3 3AF, UK; Liverpool Centre for Cardiovascular Science, University of Liverpool, Liverpool John Moores University and Liverpool Heart and Chest Hospital, Liverpool, UK; Department of Cardiovascular and Metabolic Medicine, Institute of Life Course and Medical Sciences, University of Liverpool, Liverpool L7 8TX, UK; Registry of Senior Australians, South Australian Health and Medical Research Institute, Adelaide, SA, Australia; Liverpool Centre for Cardiovascular Science, University of Liverpool, Liverpool John Moores University and Liverpool Heart and Chest Hospital, Liverpool, UK; School of Pharmacy and Biomolecular Sciences, Liverpool John Moores University, Liverpool L3 3AF, UK; Population Data Science, Swansea University Medical School, Swansea University, Swansea, Wales SA2 8PP, UK; Population Data Science, Swansea University Medical School, Swansea University, Swansea, Wales SA2 8PP, UK; Population Data Science, Swansea University Medical School, Swansea University, Swansea, Wales SA2 8PP, UK; Population Data Science, Swansea University Medical School, Swansea University, Swansea, Wales SA2 8PP, UK; Tritech Institute, Hywel Dda University Health Board, Bynea, Llanelli SA14 9TE, UK; Department of Renal Medicine, East Kent Hospital NHS Foundation Trust, Ashford TN24 0LZ, UK; Department of Geriatric Medicine, Bunbury Regional Hospital, WA Country Health Service – South West, Bunbury 6230, Australia; Division of Internal Medicine, University of Western Australia, Perth WA 6009, Australia; Curtin Medical School, Faculty of Health Sciences, Curtin University, Perth WA 6845, Australia; Population Data Science, Swansea University Medical School, Swansea University, Swansea, Wales SA2 8PP, UK; Department of Public Health, Policy and Systems, Institute of Population Health, University of Liverpool, Liverpool L69 3GF, UK; Liverpool Centre for Cardiovascular Science, University of Liverpool, Liverpool John Moores University and Liverpool Heart and Chest Hospital, Liverpool, UK; Department of Cardiovascular and Metabolic Medicine, Institute of Life Course and Medical Sciences, University of Liverpool, Liverpool L7 8TX, UK; Danish Center for Health Services Research, Department of Clinical Medicine, Aalborg University, Aalborg DK-9220, Denmark; Liverpool Centre for Cardiovascular Science, University of Liverpool, Liverpool John Moores University and Liverpool Heart and Chest Hospital, Liverpool, UK; Department of Cardiovascular and Metabolic Medicine, Institute of Life Course and Medical Sciences, University of Liverpool, Liverpool L7 8TX, UK; Danish Center for Health Services Research, Department of Clinical Medicine, Aalborg University, Aalborg DK-9220, Denmark

**Keywords:** atrial fibrillation, care homes, health outcomes, older people, integrated care

## Abstract

**Background:**

The Atrial fibrillation Better Care (ABC) pathway is the gold-standard approach to atrial fibrillation (AF) management, but the effect of implementation on health outcomes in care home residents is unknown.

**Objective:**

To examine associations between ABC pathway adherence and stroke, transient ischaemic attack, cardiovascular hospitalisation, major bleeding, mortality and a composite of all these outcomes in care home residents.

**Methods:**

A retrospective cohort study of older care home residents (≥65 years) in Wales with AF was conducted between 1 January 2003 and 31 December 2018 using the Secure Anonymised Information Linkage Databank. Adherence to the ABC pathway was assessed at care home entry using pre-specified definitions. Cox proportional hazard and competing risk models were used to estimate the risk of health outcomes according to ABC adherence.

**Results:**

From 14,493 residents (median [interquartile range] age 87.0 [82.6–91.2] years, 35.2% male) with AF, 5,531 (38.2%) were ABC pathway adherent. Pathway adherence was not significantly associated with risk of the composite outcome (adjusted hazard ratio, 95% confidence interval [CI]: 1.01 [0.97–1.05]). There was a significant independent association observed between ABC pathway adherence and a reduced risk of myocardial infarction (0.70 [0.50–0.98]), but a higher risk of haemorrhagic stroke (1.59 [1.06–2.39]). ABC pathway adherence was not significantly associated with any other individual health outcomes examined.

**Conclusion:**

An ABC adherent approach in care home residents was not consistently associated with improved health outcomes. Findings should be interpreted with caution owing to difficulties in defining pathway adherence using routinely collected data and an individualised approach is recommended.

## Key Points

- Implementation and definition of an Atrial fibrillation Better Care (ABC) approach in older care home residents is complex.
- Adherence to ABC using pre-specified routine data definitions was not consistently associated with improved health outcomes.
- ABC implementation should be individualised and focus on medication prescription, active non-prescription and de-prescribing.
- Development of an internationally comparable routine data definition of ABC adherence would be beneficial for future studies.

## Introduction

In Wales, approximately one in six older adults living in care homes, also known as nursing homes or residential aged care, have a diagnosis of atrial fibrillation (AF). For these individuals, AF has been associated with a higher risk of ischaemic stroke, cardiovascular hospitalisations, and cardiovascular and all-cause mortality [1]. Due to the high prevalence of multi-morbidity, frailty, cognitive impairment and polypharmacy in this population, effective management of AF can be complex [2].

The net clinical benefit of oral anticoagulants (OACs) compared to no treatment or antiplatelet agents is clear for most older adults with AF, as the reduction in risk of stroke typically outweighs a potential increased risk of bleeding [3]. Despite this, more than one quarter of older adults moving to care homes in Wales with AF may not be prescribed OACs, and increasing age is a potential factor associated with non-prescription of OACs [4]. However, there is evidence that there has been an increase in the use of OACs over time in older adults with AF [4, 5]. For instance, the proportion of people with AF prescribed OACs in care homes in Wales has substantially increased from 33% in 2003 to 73% in 2018 [4]. Similarly, in a large study of older adults aged ≥80 years in the USA, the proportion of people with AF prescribed OACs also increased significantly following the introduction of non-vitamin K antagonist OACs [6].

The Atrial fibrillation Better Care (ABC) pathway was developed as a simple, holistic and integrated approach to the management of AF [7]. The use of the ABC pathway has been recommended by international clinical guidelines and consists of three components to (A) avoid stroke with the use of oral anticoagulants, (B) better symptom management with rate or rhythm control, and (C) improve management of cardiovascular and other comorbidities [8]. Observational studies in people with AF have consistently suggested that adherence to the ABC pathway is associated with a lower risk of major adverse outcomes such as stroke and mortality, including in older adults with frailty, polypharmacy or multi-morbidity [9–12]. Definitions of adherence to A, B and C pathway components used in previous analyses are variable and were determined by availability of relevant prescription, laboratory and clinical data [9–12].

To date, no previous study has examined the applicability of the ABC pathway and its associations with clinical outcomes for older adults living in care homes. Many older adults living in care homes are approaching the end of life, and the proportion of deaths within 1 year of admission to care homes in Europe has been estimated at 42% [13]. There have been increasing efforts to de-prescribe medications for older adults approaching the end of life due to the potential negative effects of polypharmacy [14, 15]. This adds a layer of complexity in optimising medicine management in this population. For instance, in the ABC pathway, cardiovascular comorbidity management should be optimised, which would typically involve the use of statins for conditions such as coronary artery disease. However, randomised control trial evidence has suggested that the discontinuation of statins is safe and may improve quality of life in adults with a life expectancy less than 1 year [16].

In this study, the aim is to examine associations between AF management in accordance with the ABC pathway and outcomes including stroke, transient ischaemic attack (TIA), cardiovascular hospitalisation, major bleeding and mortality in older adults living in care homes in Wales.

## Methods

A retrospective cohort study was conducted using information within the Secure Anonymised Information Linkage (SAIL) Databank on CARE home residents in Wales aged ≥65 years with a record of AF (any sub-type or atrial flutter) prior to moving to a care home between 1 January 2003 and 31 December 2018. The methodology of this project work (SAIL CARE-AF) has previously been published [1, 4]. See Supplementary Methods for more information on data contained within the SAIL Databank.

**Figure 1:**
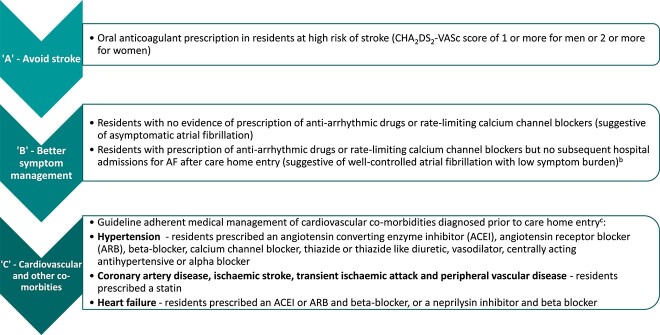
Definitions used to assess adherence to individual ABC pathway components at care home entry^a^. ^a^Prescription of medications within 6 months prior to care home entry used as a proxy for prescription at care home entry; ^b^subsequent hospital admissions for atrial fibrillation identified if ICD10-code ‘I48—atrial fibrillation and flutter’ was listed at any position (including the primary position) within the PEDW episode table after care home entry until end of follow-up (resident died, moved out of Wales or end of study (31 December 2018)); ^c^residents with no cardiovascular comorbidities prior to care home entry were also classified as ‘C’ adherent to maintain representativeness of the cohort.

Adherence to individual ABC pathway components was assessed at care home entry using pre-specified definitions (Figure 1) based on clinical guidelines for the management of AF and adapted from definitions of A, B and C components used in previous analyses of ABC adherence [10, 17–27]. ‘A’ was defined as OAC prescription in residents at high risk of stroke (CHA_2_DS_2_-VASc score of 1 or more for men or 2 or more for women); ‘B’ included residents with asymptomatic AF or AF with well-controlled symptoms, defined as no evidence of prescription of anti-arrhythmic drugs or rate-limiting calcium channel blockers prior to care home entry, or prescription of either of these two drug classes with no subsequent hospital admissions for AF after care entry; ‘C’ was defined as guideline-adherent medical management of hypertension, coronary artery disease, ischaemic stroke, TIA, peripheral vascular disease and heart failure that had been diagnosed prior to care home entry (Figure 1). Residents with no cardiovascular comorbidities prior to care home entry were also classified as ‘C’ adherent. This was to maintain representativeness of the AF cohort, as not all care home residents with AF have cardiovascular comorbidities. Prescription of medications within 6 months prior to care home entry was used as a proxy for prescription at care home entry. Residents who fulfilled all three ABC pathway criteria were classified as ‘ABC’ adherent, and those who did not were classified as ‘non-ABC’ adherent.

### Outcomes

Outcomes of interest were ischaemic stroke, haemorrhagic stroke, stroke of unknown classification, TIA, cardiovascular hospitalisation, major bleeding, myocardial infarction, cardiovascular and all-cause mortality, and a composite of these outcomes (excluding cardiovascular mortality). All participants were followed up until they died, moved out of Wales or until the end of study (31 December 2018). Data were requested up until December 2018 to prevent confounding arising from the effects of COVID-19 pandemic on mortality rates and the completeness of routinely collected data.

### Statistical analyses

The incidence per 1,000 person-years of all outcomes was reported for residents by ABC adherence on care home entry. Using Cox proportional hazard regression models, the hazard ratios (HRs) for the composite and individual outcomes were estimated according to ABC adherence at care home entry. Fine–Grey competing risk models were also used to estimate the sub-distribution hazard ratio (sHR) of individual outcomes using mortality as a competing risk. All residents were followed up until they died, moved out of Wales or until the end of study (31 December 2018). All analyses were adjusted for age, sex, Welsh Index of Multiple Deprivation (Version 2011), electronic Frailty Index, smoking history, dementia, pulmonary disease, cancer, peptic ulcer disease, stroke (CHA_2_DS_2_-VASc) and bleeding (HAS-BLED) risk assessment scores [28, 29]. A sensitivity analysis was performed to adjust for the individual components that constitute CHA_2_DS_2_-VASc and HAS-BLED risk assessment scores, listed in Supplementary Table 4. Multicollinearity was assessed using the Variance Inflation Factor (Supplementary Tables 5 and 6). Proportional hazards assumption was tested by visual inspection of plots of Schoenfeld residuals.

Secondary analyses were also undertaken to examine associations between partial ABC adherence (0 or 1 components fulfilled versus 2 or 3, 0 components fulfilled versus AB or BC or AC groups) and the risk of the composite and individual outcomes.

## Results

### Characteristics of study cohort

From the SAIL CARE-AF cohort (*n* = 86,602) [1, 4], there were 14,493 (16.7%) people (median [interquartile range, IQR] age 87.0 [82.6–91.2] years, *n* = 5,103, 35.2% male) with a diagnosis of AF prior to care home entry between 2003 and 2018. Of these, 5,531 (38.2%) fulfilled all three ABC criteria (ABC adherent group) at the point of care entry. Adherence to individual ABC pathway components were ‘A’ *n* = 7,057 (48.7%), ‘B’ *n* = 13,702 (94.5%) and ‘C’ *n* = 11,068 (76.4%) (Supplementary Figure 1). There were 2,949 residents without any documented cardiovascular comorbidity who were classified as ‘C’ adherent. Baseline characteristics according to ABC status are reported in Table 1. Residents in the ABC adherent group were slightly younger than those who did not fulfil all ABC criteria (non-ABC adherent group) (median [IQR] 86.2 [81.8–90.1] versus 87.6 [83.1–91.8] years, respectively), but a higher proportion had cardiovascular comorbidities (hypertension, 3,195 [57.8%] versus 3,772 [42.1%]; coronary artery disease (including myocardial infarction, angina and other acute or chronic ischaemic heart disease), 500 [9.0%] versus 631 [7.0%]; ischaemic stroke, 1,090 [19.7%] versus 1,142 [12.7%]; TIA, 353 [6.4%] versus 443 [4.9%]; peripheral vascular disease, 421 [7.6%] versus 434 [4.8%]; and heart failure, 1,790 [32.4%] versus 2,414 [26.9%]) (Table 1). Both ABC and non-ABC adherent groups were assessed to have the same median stroke risk on care home entry (median CHA_2_DS_2_-VASc of 4), but the ABC group had a higher bleeding risk (median HAS-BLED of 3 versus 2). On care home entry, antiplatelets were the most commonly prescribed medication in 10,285 (71.0%) of residents with AF. Less than half of residents were prescribed an anticoagulant (*n* = 7,057, 48.9%), beta blocker (*n* = 5,350, 36.9%), angiotensin-converting enzyme inhibitor (*n* = 4,274, 29.5%) or angiotensin receptor blocker (*n* = 1,002, 6.9%) (Table 1).

**Table 1:** Characteristics of adults aged ≥65 years within the SAIL databank on care home entry (2003–18), by ABC adherence

Characteristics	All residents, *n* (%) (*n* = 14,493)	ABC group^e^, *n* (%) (*n* = 5,531)	Non-ABC group^e^, *n* (%) (*n* = 8,962)
**Demographics**			
Age, median (IQR)	87.0 (82.6, 91.2)	86.2 (81.8, 90.1)	87.6 (83.1, 91.8)
Age category	770 (5.3)	340 (6.1)	430 (4.8)
65–74 years			
75–84 years	4,682 (32.3)	2,016 (36.4)	2,666 (29.7)
85–94 years	7,859 (54.2)	2,903 (52.5)	4,956 (55.3)
≥95 years	1,182 (8.2)	272 (4.9)	910 (10.2)
Male	5,103 (35.2)	2,114 (38.2)	2,989 (33.4)
WIMD quintile	2,459 (17.1)	930 (17.1)	1,529 (17.2)
1 (most deprived)			
2	3,053 (21.3)	1,173 (21.6)	1,880 (21.1)
3	3,435 (23.9)	1,308 (24.1)	2,127 (23.9)
4	2,842 (19.8)	1,061 (19.5)	1,781 (20.0)
5 (least deprived)	2,554 (17.8)	959 (17.7)	1,595 (17.9)
**Frailty**			
No frailty	1,864 (12.9)	178 (3.2)	1,686 (18.8)
Mild	3,714 (25.6)	1,173 (21.2)	2,541 (28.4)
Moderate	5,145 (35.5)	2,235 (40.4)	2,910 (32.5)
Severe	3,770 (26.0)	1945 (35.2)	1,825 (20.4)
**Stroke risk**			
CHA_2_DS_2_-VASc score, median (IQR)	4 (3, 5)	4 (3, 5)	4 (3, 5)
**Bleed risk**			
HAS-BLED score, median (IQR)	3 (2, 3)	3 (2, 4)	2 (2, 3)
**Social history**			
Smoking history	3,996 (27.6)	1,891 (34.2)	2,105 (23.5)
Alcoholism	1,223 (8.4)	570 (10.3)	653 (7.3)
Heavy drinker	224 (1.5)	84 (1.5)	140 (1.6)
**Comorbidities**			
Any stroke	2,929 (20.2)	1,396 (25.2)	1,533 (17.1)
Stroke (unknown)	618 (4.3)	290 (5.2)	328 (3.7)
Ischaemic stroke	2,232 (15.4)	1,090 (19.7)	1,142 (12.7)
Haemorrhagic stroke	336 (2.3)	155 (2.8)	181 (2.0)
TIA	796 (5.5)	353 (6.4)	443 (4.9)
Myocardial infarction	1,131 (7.8)	500 (9.0)	631 (7.0)
Heart failure	4,204 (29.0)	1,790 (32.4)	2,414 (26.9)
Alzheimer’s disease	162 (1.1)	55 (1.0)	107 (1.2)
Vascular dementia	552 (3.8)	214 (3.9)	338 (3.8)
Young onset dementia^a^	<5 (<1)	<5 (<1)	<5 (<1)
Other dementia^b^	542 (3.7)	190 (3.4)	352 (3.9)
Asthma	1,214 (8.4)	510 (9.2)	704 (7.9)
COPD	1,794 (12.4)	720 (13.0)	1,074 (12.0)
Other pulmonary disease^a^	<10 (<0.1)	<10 (<0.1)	<10 (<0.1)
Peptic ulcer	422 (2.9)	162 (2.9)	260 (2.9)
Diabetes	728 (5.0)	348 (6.3)	380 (4.2)
Renal disease	1,052 (7.3)	481 (8.7)	571 (6.4)
Liver disease	45 (0.3)	19 (0.3)	26 (0.3)
Cancer	2,236 (15.4)	953 (17.2)	1,283 (14.3)
Hypertension	6,967 (48.1)	3,195 (57.8)	3,772 (42.1)
Dyslipidaemia	1,765 (12.2)	924 (16.7)	841 (9.4)
Vascular disease	855 (5.9)	42 (7.6)	434 (4.8)
Aortic plaque^a^	<10 (<0.1)	<10 (<0.1)	<10 (<0.1)
Major bleeding	2,635 (18.2)	1,221 (22.1)	1,414 (15.8)
Thromboembolism	464 (3.2)	267 (4.8)	197 (2.2)
**Medications** ^c^			
Angiotensin-converting enzyme inhibitor	4,274 (29.5)	2,185 (39.5)	2,089 (23.3)
Angiotensin receptor blocker	1,002 (6.9)	548 (9.9)	454 (5.1)
Beta blocker	5,350 (36.9)	3,033 (54.8)	2,317 (25.9)
Anti-arrhythmic	664 (4.6)	111 (2.0)	553 (6.2)
Glycoside	4,752 (32.8)	2,101 (38.0)	2,651 (29.6)
Rate-limiting calcium channel blocker	718 (5.0)	133 (2.4)	585 (6.5)
Dihydropyridine calcium channel blocker	1,733 (12.0)	819 (14.8)	914 (10.2)
Thiazide or thiazide-like diuretic	1,057 (7.3)	454 (8.2)	603 (6.7)
Alpha blocker	393 (2.7)	196 (3.5)	197 (2.2)
Vasodilator	^d^	12 (0.2)	<5 (<1)
Centrally acting antihypertensive	63 (0.4)	29 (0.5)	34 (0.4)
Statin	7,457 (51.5)	4,134 (74.7)	3,323 (37.1)
Oral anticoagulant	7,057 (48.7)	5,531 (100)	1,526 (17.0)
Antiplatelet	10,285 (71.0)	4,135 (74.8)	6,150 (68.6)
Non-steroidal anti-inflammatory	6,210 (42.8)	2,811 (50.8)	3,399 (37.9)

^a^Estimate only to maintain resident anonymity

^b^Other or unspecified dementia

^c^Record of prescription within 6 months prior to care home entry used as a proxy for prescription on care entry

^d^Not possible to calculate

^e^ABC group, residents who fulfilled all three ABC criteria; non-ABC group, residents who did not fulfil all ABC criteria, but may have fulfilled one or two components

AF, atrial fibrillation; CHA_2_DS_2_-VASc, stroke risk assessment scoring 1 point each for female sex, age 65–74 years, history of heart failure, diabetes, hypertension, vascular disease and 2 points each for history of stroke/transient ischaemic attack/venous thromboembolism and age ≥75 years; COPD, chronic obstructive pulmonary disease; HAS-BLED, bleeding risk assessment scoring 1 point each for age >65 years, uncontrolled hypertension, liver disease, renal disease, harmful alcohol use, stroke history, prior major bleeding or a predisposition to bleeding, labile international normalised ratio and medication usage predisposing to bleeding; IQR, interquartile range; TIA, transient ischaemic attack; WIMD, Welsh Index of Multiple Deprivation

### Incidence and risk of adverse outcomes according to ABC pathway adherence

The median [IQR] follow-up of the cohort was 402.5 [125–930] days. The crude number of events and incidence of outcomes per 1,000 person-years by ABC adherence status are reported in Table 2. In Cox regression analyses, ABC adherence was not significantly associated with the risk of the composite outcome, ischaemic stroke, stroke of unknown origin, TIA, major bleeding, and cardiovascular or all-cause mortality after adjusting for covariates. ABC adherence was associated with a significantly reduced risk of myocardial infarction (adjusted hazard ratio [aHR], 95% CI: 0.70 [0.50–0.98], *P* = 0.040), but a higher risk of haemorrhagic stroke (1.59 [1.06–2.39], *P* = 0.025) (Table 2). These findings were corroborated when individual components of the CHA_2_DS_2_-VASc and HAS-BLED risk assessments were adjusted for in sensitivity analyses (Table 2). The Variance Inflation Factor was <1.7 for all covariates (Supplementary Tables 5 and 6). There was no evidence that the proportional hazards had been violated for individual covariates.

**Table 2:** Incidence and risk of the composite outcome, stroke, transient ischaemic attack, cardiovascular hospitalisation, major bleeding and mortality in care home residents aged ≥65 years by ABC status on care home entry (2003–18)—Cox regression analysis

	Number of events, *n*^c^ (%)	Incident rate per 1,000 person-years (95% CI)	Unadjusted hazard ratio (95% CI), *P* value	Adjusted hazard ratio^a^ (95% CI), *P* value	Adjusted hazard ratio^b^ (95% CI), *P* value
**Composite** ^d^					
Non-ABC	8,129 (90.7)	577.29 (564.84–590.02)	1	1	1
ABC	4,539 (82.1)	607.66 (590.15–625.69)	1.03 (0.99–1.07), *P* = 0.129	1.01 (0.97–1.05), *P* = 0.588	1.02 (0.98–1.06), *P* = 0.333
**Ischaemic stroke**					
Non-ABC	258 (2.9)	15.74 (13.93–17.78)	1	1	1
ABC	150 (2.7)	17.22 (14.67–20.22)	1.08 (0.88–1.32), *P* = 0.468	1.16 (0.93–1.44), *P* = 0.181	1.16 (0.94–1.44), *P* = 0.169
**Haemorrhagic stroke**					
Non-ABC	58 (0.6)	3.51 (2.72–4.55)	1	1	1
ABC	51 (0.9)	5.83 (4.43–7.67)	**1.57 (1.08**–**2.29), *P* = 0.019**	**1.59 (1.06**–**2.39), *P* = 0.025**	**1.58 (1.06**–**2.37), *P* = 0.026**
**Stroke of unknown origin**					
Non-ABC	82 (0.9)	4.93 (3.97–6.13)	1	1	1
ABC	55 (1.0)	6.12 (4.67–7.99)	1.18 (0.84–1.67), *P* = 0.343	1.04 (0.72–1.51), *P* = 0.816	1.06 (0.73–1.52), *P* = 0.773
**Transient ischaemic attack**					
Non-ABC	76 (0.8)	4.64 (3.70–5.81)	1	1	1
ABC	44 (0.8)	5.07 (3.77–6.81)	1.07 (0.74–1.55), *P* = 0.727	1.12 (0.76–1.65), *P* = 0.579	1.12 (0.76–1.65), *P* = 0.571
**Cardiovascular hospitalisation**					
Non-ABC	2,122 (23.7)	150.39 (144.10–156.94)	1	1	1
ABC	1,265 (22.9)	169.43 (160.32–179.06)	**1.08 (1.01**–**1.16), *P* = 0.022**	1.00 (0.93–1.08), *P* = 0.908	1.03 (0.96–1.11), *P* = 0.436
**Major bleeding**					
Non-ABC	394 (4.4)	24.39 (22.09–26.94)	1	1	1
ABC	246 (4.4)	29.15 (25.72–33.04)	1.16 (0.99–1.36), *P* = 0.066	1.15 (0.97–1.37), *P* = 0.100	1.17 (0.99–1.39), *P* = 0.070
**Myocardial infarction**					
Non-ABC	140 (1.6)	8.52 (7.22–10.05)	1	1	1
ABC	54 (1.0)	6.08 (4.64–7.96)	**0.70 (0.51**–**0.96), *P* = 0.026**	**0.70 (0.50**–**0.98), *P* = 0.040**	**0.72 (0.52**–**1.00), *P* = 0.054**
**All-cause mortality**					
Non-ABC	7,960 (88.8)	478.72 (468.29–489.38)	1	1	1
ABC	4,362 (78.9)	493.82 (479.32–508.76)	1.01 (0.98–1.05), *P* = 0.453	1.02 (0.98–1.06), *P* = 0.393	1.02 (0.99–1.07), *P* = 0.224
**Cardiovascular mortality**					
Non-ABC	731 (8.2)	44.00 (40.92–47.32)	1	1	1
ABC	371 (6.7)	41.88 (37.81–46.40)	0.93 (0.82–1.05), *P* = 0.245	0.92 (0.81–1.05), *P* = 0.238	0.93 (0.82–1.06), *P* = 0.300

ABC, Atrial fibrillation Better Care; AF, atrial fibrillation; CI, confidence interval

Significant results in **bold**

^a^Hazard ratio adjusted for age, sex, Welsh Index of Multiple Deprivation, electronic Frailty Index, smoking, dementia, pulmonary disease, cancer, peptic ulcer disease, CHA_2_DS_2_VASc and HAS-BLED risk assessment scores

^b^Hazard ratio adjusted for age, sex, Welsh Index of Multiple Deprivation, electronic Frailty Index, smoking, dementia, pulmonary disease, cancer, peptic ulcer disease and individual components that constitute CHA_2_DS_2_VASc and HAS-BLED risk assessment scores

^c^ *n* = 8,962 people in non-ABC group and *n* = 5,531 people in ABC group, used as denominator for calculation of percentage

^d^Composite of ischaemic stroke, haemorrhagic stroke, stroke of unknown classification, transient ischaemic attack, cardiovascular hospitalisation, major bleeding, myocardial infarction and all-cause mortality

When mortality was considered as a competing risk, ABC adherence remained significantly associated with a reduced risk of myocardial infarction (adjusted sub-distribution hazard ratio [aSHR], 95% CI: 0.66 [0.47–0.93], *P* = 0.018) and a significantly higher risk of haemorrhagic stroke (1.54 [1.02–2.32], *P* = 0.038) (Table 3). There were no statistically significant associations observed between ABC adherence and other adverse health outcomes (Table 3).

**Table 3:** Incidence and risk of stroke, transient ischaemic attack, cardiovascular hospitalisation, major bleeding and mortality in care home residents aged ≥65 years by ABC status on care home entry (2003–18)—competing risk analysis

	Unadjusted sub-distribution hazard ratio (95% CI), *P* value	Adjusted sub-distribution hazard ratio^a^ (95% CI), *P* value	Adjusted sub-distribution hazard ratio^b^ (95% CI), *P* value
**Ischaemic stroke**			
Non-ABC	1	1	1
ABC	1.00 (0.82–1.23), *P* = 0.969	1.09 (0.87–1.36), *P* = 0.466	1.08 (0.86–1.35), *P* = 0.509
**Haemorrhagic stroke**			
Non-ABC	1	1	1
ABC	**1.50 (1.03**–**2.19), *P* = 0.035**	**1.54 (1.02**–**2.32), *P* = 0.038**	**1.51 (1.01**–**2.25), *P* = 0.044**
**Stroke of unknown origin**			
Non-ABC	1	1	1
ABC	1.12 (0.79–1.58), *P* = 0.528	1.00 (0.67–1.48), *P* = 0.989	1.00 (0.68–1.48), *P* = 0.969
**Transient ischaemic attack**			
Non-ABC	1	1	1
ABC	1.00 (0.69–1.45), *P* = 0.994	1.05 (0.71–1.55), *P* = 0.799	1.05 (0.72–1.54), *P* = 0.800
**Cardiovascular hospitalisation**			
Non-ABC	1	1	1
ABC	1.03 (0.96–1.11), *P* = 0.375	0.96 (0.89–1.04), *P* = 0.309	0.98 (0.91–1.06), *P* = 0.600
**Major bleeding**			
Non-ABC	1	1	1
ABC	1.09 (0.93–1.28), *P* = 0.306	1.09 (0.92–1.30), *P* = 0.334	1.10 (0.92–1.30), *P* = 0.291
**Myocardial infarction**			
Non-ABC	1	1	1
ABC	**0.65 (0.48**–**0.90), *P* = 0.008**	**0.66 (0.47**–**0.93), *P* = 0.018**	**0.67 (0.48**–**0.95), *P* = 0.025**
**All-cause mortality**			
Non-ABC	1	1	1
ABC	1.01 (0.98–1.05), *P* = 0.453^c^	1.02 (0.98–1.06), *P* = 0.393^c^	1.02 (0.99–1.07), *P* = 0.224^c^
**Cardiovascular mortality**			
Non-ABC	1	1	1
ABC	0.93 (0.82–1.05), *P* = 0.245^c^	0.92 (0.81–1.05), *P* = 0.238^c^	0.93 (0.82–1.06), *P* = 0.300^c^

ABC, Atrial fibrillation Better Care; AF, atrial fibrillation; CI, confidence interval

Significant results in **bold**

^a^Main analysis—sub-distribution hazard ratio adjusted for age, sex, Welsh Index of Multiple Deprivation, electronic Frailty Index, smoking, dementia, pulmonary disease, cancer, peptic ulcer disease, CHA_2_DS_2_VASc and HAS-BLED risk assessment scores

^b^Sensitivity analysis—sub-distribution hazard ratio adjusted for age, sex, Welsh Index of Multiple Deprivation, electronic Frailty Index, smoking, dementia, pulmonary disease, cancer, peptic ulcer disease and individual components that constitute CHA_2_DS_2_VASc and HAS-BLED risk assessment scores

^c^Hazard ratio not sub-distributed, standard Cox regression analysis

In secondary analyses, adherence to an increasing number of ABC criteria (0 or 1 components fulfilled versus 2 or 3) was associated with a reduced risk of the composite outcome (0.949 [0.903–0.997], *P* = 0.037) and cardiovascular hospitalisation (0.87 [0.79–0.96], *P* = 0.005) when two pathway components were fulfilled (versus 0 or 1) (Supplementary Table 7). Full adherence to the ABC pathway (versus 0 or 1 criteria fulfilled) was associated with a significantly higher risk of haemorrhagic stroke (1.95 [1.05–3.65], *P* = 0.035). Results from competing risk analyses are reported in Supplementary Table 8.

### Associations between partial ABC adherence and outcomes

Fulfilment of the AC and BC criteria was associated with a reduced risk of the composite outcome compared to non-adherence to AC and BC (HRs [95% CIs] 0.84 [0.76–0.93], *P* = 0.001 and 0.96 [0.93–1.00], *P* = 0.045, respectively). In contrast, adherence to AB criteria was associated with a higher risk of the composite outcome when compared to AB non-adherence (1.09 [1.02–1.17], *P* = 0.011) (Supplementary Table 9). Adherence to the BC criteria was associated with a reduced risk of cardiovascular hospitalisation (aHR 0.86 [0.80–0.93], *P* < 0.001) (Supplementary Table 9). Adherence to the AC criteria was also associated with a reduced risk of all-cause mortality (aHR 0.78 [0.70–0.87], *P* < 0.001), but an increased risk of cardiovascular hospitalisation (aHR 1.40 [1.19–1.63], *P* < 0.001). There was no association between AB, BC or AC groups and the risk of ischaemic stroke, haemorrhagic stroke, stroke of unknown origin, TIA, major bleeding, myocardial infarction or cardiovascular mortality (Supplementary Table 9). Results from competing risk analyses are reported in Supplementary Table 10. Similarly, no associations were observed between AB, BC or AC groups and ischaemic stroke, haemorrhagic stroke, stroke of unknown origin, TIA, myocardial infarction or cardiovascular mortality. Adherence to the BC criteria was associated with a reduced risk of cardiovascular hospitalisation, and adherence to the AC criteria continued to be associated with a reduced risk of all-cause mortality and an increased risk of cardiovascular hospitalisation (Supplementary Table 7).

## Discussion

To our knowledge, this is the first study to examine associations between ABC pathway adherence for AF management and adverse health outcomes in a large population-based study exclusively of older adults living in care homes in Wales. The principal findings from this study are as follows: (i) complete adherence to the ABC pathway was not significantly associated with the risk of the composite outcome; (ii) ABC pathway adherence was associated with a significantly reduced risk of myocardial infarction, but an increased risk of haemorrhagic stroke; and (iii) when higher mortality rates were accounted for in this cohort, pathway adherence continued to be associated with a reduced risk of myocardial infarction but an increased risk of haemorrhagic stroke.

This is the first study that has not consistently demonstrated ABC pathway adherence to significantly reduce the risk of a composite of adverse health outcomes. This contrasts with findings from two observational data registries including clinically complex cohorts of adults [10, 11]. ABC pathway adherence was reported to be significantly associated with a reduced risk of the composite outcome including all-cause hospitalisation and all-cause death in people with multi-morbidity (*n* = 1,723, HR 0.61, 95% CI 0.44–0.85), polypharmacy (*n* = 1,222, HR 0.68, 95% CI 0.47–1.00) and recent hospitalisation (*n* = 1,360, HR 0.59, 95% CI 0.42–0.85) [10]. In 8,289 people presenting with at least one of the following, frailty, multi-morbidity and/or polypharmacy, full adherence to the ABC pathway was associated with a reduced risk of the composite outcome of thromboembolic events, acute coronary syndrome and cardiovascular death (aHR 0.70, 95% CI 0.58–0.85) [11]. Differences with the current study findings likely result from the use of different registries, variations in the average age of participants, definitions of ABC adherence, composite outcomes and measures of frailty. Previous studies also had longer average follow-ups of 3.7 (IQR 2.8–4.6) years [10] and 730 (IQR 701–749) days [11], compared to 402 days in the current analyses.

The association between ABC adherence and lower risk of myocardial infarction but higher risk of haemorrhagic stroke found in this study may be driven by the high incidence of concomitant OAC and antiplatelet therapy reported for 4,135 (74.8%) ABC adherent residents at the point of care entry. Investigation of the appropriateness of antithrombotic therapy and the temporal association between prescription of antithrombotics and incident haemorrhagic stroke was not possible within the constraints of SAIL. A small proportion of residents were recorded to have concomitant hospitalisation events due to AF (used in the definition of ‘B’ adherence) and myocardial infarction. This may have resulted in some residents being mislabelled as ‘B’ non-adherent if AF was recorded as a comorbidity in residents hospitalised with MI, rather than a primary cause of admission. All associations in this study should be interpreted with caution. Despite some associations reaching statistical significance, it is difficult to gauge if these are clinically meaningful due to the complexity of the study cohort and reliance on retrospective, routinely collected health data. Furthermore, results from analyses to investigate associations between partial ABC adherence and outcomes were inconsistent and it is not possible to draw any definitive conclusions.

A retrospective registry study in Korea investigated application of the ABC pathway in people with low (*n* = 221, 542), intermediate (*n* = 37, 341) and high (*n* = 4, 101) frailty risk using ICD-10 diagnostic codes to calculate the Hospital Frailty Risk Score and frailty category [9]. Benefits of ABC pathway adherence were observed in the high frailty risk group for all-cause death (HR 0.74, 95% CI 0.56–0.97) but not for the composite outcome of all-cause death, ischaemic stroke, heart failure admission, acute myocardial infarction and major bleeding (HR 0.79, 95% CI 0.59–1.05) [9]. The authors do not speculate why this may be the case; however, it highlights the importance of careful, individualised application of the ABC pathway in people with severe frailty to ensure benefit is maximised and harm reduced as much as possible. In this study, a higher proportion of the ABC adherent group were classified as severely frail using the electronic Frailty Index compared to the non-ABC adherent group (ABC adherent *n* = 1,945 [35.2%] versus non-ABC adherent *n* = 1,825 [20.4%]).

Prescription of medication for AF management in care home residents must be individualised after assessment of the net risk versus benefit of treatment in the context of often guarded prognoses. Person-centric decisions that may include non-prescription of medication and de-prescribing are integral to medicines optimisation and it is paramount that maintenance and improvement of quality of life underpin all treatment decisions [30–32]. Due to the inherent complexity of medication management in this cohort, it could be argued that application of standardised definitions of ABC pathway adherence is too simplistic and a ‘one size fits all’ management approach is flawed. This may help explain the findings of this study. It is likely that some residents will not be adherent to the ABC pathway by definition as a result of active decision-making, with a focus on quality of life in the context of prognosis, consideration of other chronic conditions and risk of adverse medication-related effects.

### Strengths and limitations

To date, this is the only study that has investigated application of the ABC pathway in older care home residents. Use of routinely collected data on a national scale spanning a decade maximises representativeness and generalisability of study findings. However, reliance on positive recordings of diagnoses and medication prescription 6 months prior to care entry as a proxy for ABC adherence and attainment of clinic end-points (for example, blood pressure control and normalisation of cholesterol levels) may have resulted in misclassification of residents to the ABC adherent and non-ABC adherent groups. Furthermore, it is not possible to explore temporal associations between prescription of different antithrombotics prior to care home entry and this could have influenced the study’s results. Outcome events in any position within the PEDW episode table were recorded; therefore, it is possible some may have been incorrectly inputted as a relevant comorbidity rather than a cause of hospital admission. It is also possible that the proportion of residents’ adherent to the ‘B’ component of the pathway was overestimated using this study’s definition of AF symptom management and this could have impacted the findings. Whilst the follow-up period in this study was less than previous studies of clinically complex cohorts, it is similar to a reported length of stay in care homes (time from care home entry to death) of 462 days [33]. This may also explain the lower event rates for some adverse outcomes including haemorrhagic stroke, stroke or unknown classification, TIA and MI, which could have influenced study results.

### Clinical implications

Implementation of the ABC pathway in older care home residents is complex and may not always translate to improved health outcomes when standardised definitions of adherence to each component of the pathway are used. Pathway implementation in a real-world cohort of care home residents is recommended to explore this further, and one pilot and feasibility study of a pharmacist-led medicines optimisation for care home residents with AF using the ABC framework has recently been completed to test this [34]. An adaption of the ABC pathway could be developed for older people with multiple chronic conditions to support individualised decision-making. In future studies, assessment of ABC pathway adherence should be personalised and focus on optimal AF management with implementation of a holistic approach of medication prescription, active non-prescription and de-prescribing. Development of an internationally comparable routine data definition of the ABC pathway would also be beneficial, even if this is a requisite for change in what data are routinely collected.

## Conclusions

An ABC adherent approach to AF management in care home residents was not consistently associated with improved health outcomes. These findings may be explained by difficulties in defining pathway adherence in care home residents and an individualised approach is recommended.

## Supplementary Material

Supplementary_data_12Feb24_FINAL_clean
