# Supplementary material for: Adherence to the Atrial fibrillation Better Care pathway and the risk of adverse health outcomes in older care home residents with atrial fibrillation: a retrospective data linkage study 2003–18
Source: Age Ageing. 2024 Feb 22;53(2):afae021. doi: 10.1093/ageing/afae021 (PMC10891424; doi:10.1093/ageing/afae021)
Supplement: Supplementary_data_12Feb24_FINAL_clean [file supplementary_data_12feb24_final_clean.docx]

**Adherence to the Atrial Fibrillation Better Care pathway and the risk of adverse health outcomes in older care home residents with atrial fibrillation: a routine data linkage study 2003-2018**

**Supplementary Material**

**Contents**

[Supplementary methods 3](#_Toc156482725)

[Supplementary Table 1. ICD-10 codes to identify conditions in the Patient Episode Database for Wales (PEDW) data source. 5](#_Toc156482726)

[Supplementary Table 2. Read codes version 2 to identify conditions/medications in the Welsh Longitudinal General Practice (WLGP) data source. 20](#_Toc156482727)

[Supplementary Table 3. The RECORD statement – checklist of items, extended from the STROBE statement that should be reported in observational studies using routinely collected health data. 86](#_Toc156482728)

[Supplementary Figure 1. Adherence to the ABC pathway and its components 91](#_Toc156482729)

[Supplementary Table 4. Components of CHA_2_DS2-VASc and HAS-BLED risk assessment scores adjusted for in sensitivity analyses. 92](#_Toc156482730)

[Supplementary Table 5. Assessment of multicollinearity between covariates (including CHA_2_DS_2_VASc and HAS-BLED scores), using the Variance Inflation Factor. 93](#_Toc156482731)

[Supplementary Table 6. Assessment of multicollinearity between covariates (including individual components that constitute CHA_2_DS_2_VASc and HAS-BLED scores^a^), using the Variance Inflation Factor. 94](#_Toc156482732)

[Supplementary Table 7. Risk of the composite outcome, stroke, transient ischaemic attack, cardiovascular hospitalisation, major bleeding and mortality in care home residents aged ≥65 years by partial ABC adherence^a^ on care home entry (2003-2018) – Cox Regression Analysis. 95](#_Toc156482733)

[Supplementary Table 8. Risk of stroke, transient ischaemic attack, cardiovascular hospitalisation, major bleeding and mortality in care home residents aged ≥65 years by partial ABC adherence^a^ on care home entry (2003-2018) – Competing Risk Analysis. 97](#_Toc156482734)

[Supplementary Table 9. Risk of the composite outcome, stroke, transient ischaemic attack, cardiovascular hospitalisation, major bleeding and mortality in care home residents aged ≥65 years by partial ABC adherence^a^ on care home entry (2003-2018) – Cox Regression Analysis. 99](#_Toc156482735)

[Supplementary Table 10. Incidence and risk of stroke, transient ischaemic attack, cardiovascular hospitalisation, major bleeding and mortality in care home residents aged ≥65 years by ABC status on care home entry (2003-2018) – Competing Risk Analysis. 101](#_Toc156482736)

# Supplementary methods

Data sources (Welsh Demographic Service Dataset; WDSD, Welsh Longitudinal General Practice; WLGP, Patient Episode Database for Wales; PEDW) contained within the SAIL Databank [1, 2] provided anonymised, individual-level population scale routinely collected health and administrative data. The WLGP data provides ~80% coverage of patients and general practices in Wales. The PEDW secondary care data has 100% coverage of patients and services. Data were requested up until December 2018 to prevent confounding arising from the effects of COVID-19 pandemic on mortality rates and the completeness of routinely collected data. Extraction of care home resident specific data relied on the (CARE) home data source within the SAIL Databank [3]. This uses information available from the Care Inspectorate Wales (CIW) registry. Care homes in Wales are registered with CIW who carry out inspection and quality improvement. Primary care consultation and prescription data were extracted from WLGP using Read version 2 codes, and secondary care consultation data were extracted from PEDW using International Classification of Diseases version 10 (ICD-10) codes to ascertain residents’ co-morbidities and cardiovascular medications taken six months prior to care home entry (**Supplementary Tables 1 and 2**). Data sources were interrogated to determine the code lists and were further reviewed by clinical experts. Complete linked data were available from 1 January 2000 for the entire cohort and all participants had a minimum of 12 months of data coverage within the WLGP prior to moving to a care home. Residents without primary care records were excluded from the analysis. Date of death was determined from the Office for National Statistics Annual District Death Extract [4]. Outcome events listed at any position (including the primary position) within the PEDW episode table after care home entry were identified [5]. Episode sequences do not routinely contain information on a person’s prior co-morbidities. Reporting of studies conducted using observational routinely collected data (RECORD) 2015 guidelines were followed (**Supplementary Table 3**) [6].

**References**

1. Lyons RA, Jones KH, John G, et al. The SAIL databank: linking multiple health and social care datasets. BMC Med Inform Decis Mak. 2009;9:3.

2. Ford DV, Jones KH, Verplancke JP, et al. The SAIL Databank: building a national architecture for e-health research and evaluation. BMC Health Serv Res. 2009;9:157.

3. Hollinghurst J, Akbari A, Fry R, et al. Study protocol for investigating the impact of community home modification services on hospital utilisation for fall injuries: a controlled longitudinal study using data linkage. BMJ Open. 2018;8(10):e026290.

4. Digital Health and Care Wales (DHCW). Secure Anonymised Information Linkage (SAIL) Annual District Death Extract (ADDE). Available from: <https://web.www.healthdatagateway.org/dataset/15cf4241-abad-4dcc-95b0-8cd7c02be999> [cited 15 August 2021].

5. Health Data Research Innovation Gateway. Patient Episode Dataset for Wales (PEDW). Available from: <https://web.www.healthdatagateway.org/dataset/4c33a5d2-164c-41d7-9797-dc2b008cc852> [cited 5 December 2023].

6. Benchimol EI, Smeeth L, Guttmann A, et al. The REporting of studies Conducted using Observational Routinely-collected health Data (RECORD) statement. PLoS Med. 2015;12(10):e1001885.

# Supplementary Table 1. ICD-10 codes to identify conditions in the Patient Episode Database for Wales (PEDW) data source.

| **ICD-10 code** | **ICD-10 definition** |
| --- | --- |
| **Cardiovascular hospitalisation** | |
| I210 | Acute transmural myocardial infarction of anterior wall |
| I211 | Acute transmural myocardial infarction of inferior wall |
| I212 | Acute transmural myocardial infarction of other sites |
| I213 | Acute transmural myocardial infarction of unspecified site |
| I214 | Acute subendocardial myocardial infarction |
| I219 | Acute myocardial infarction unspecified |
| I220 | Subsequent myocardial infarction of anterior wall |
| I221 | Subsequent myocardial infarction of inferior wall |
| I228 | Subsequent myocardial infarction of other sites |
| I229 | Subsequent myocardial infarction of unspecified site |
| I230 | Haemopericardium as current complication following acute myocardial infarction |
| I231 | Atrial septal defect as current complication following acute myocardial infarction |
| I232 | Ventricular septal defect as current complication following acute myocardial infarction |
| I233 | Rupture of cardiac wall without haemopericardium as current complication following acute myocardial infarction |
| I234 | Rupture of chordae tendineae as current complication following acute myocardial infarction |
| I235 | Rupture of papillary muscle as current complication following acute myocardial infarction |
| I236 | Thrombosis of atrium, auricular appendage, and ventricle as current complications following acute myocardial infarction |
| I238 | Other current complications following acute myocardial infarction |
| I252 | Old myocardial infarction |
| I241 | Dressler syndrome (post myocardial infarction syndrome) |
| I50 | Congestive heart failure |
| I110 | Left ventricular failure |
| I130 | Hypertensive heart disease with congestive heart failure |
| I132 | Hypertensive heart and renal disease with congestive heart failure and renal failure |
| I420 | Dilated cardiomyopathy |
| I81 | Portal vein thrombosis |
| I260 | Pulmonary embolism with mention of acute cor pulmonale |
| I269 | Pulmonary embolism without mention of acute cor pulmonale |
| I270 | Primary pulmonary hypertension |
| I271 | Kyphoscoliotic heart disease |
| I272 | Other secondary pulmonary hypertension |
| I278 | Other specified pulmonary heart diseases |
| I279 | Pulmonary heart disease, unspecified |
| I280 | Arteriovenous fistula of pulmonary vessels |
| I281 | Aneurysm of pulmonary artery |
| I288 | Other specified diseases of pulmonary vessels |
| I289 | Disease of pulmonary vessels, unspecified |
| I820 | Budd-Chiari syndrome |
| I821 | Thrombophlebitis migrans (disorder of abnormal blood coagulation and thrombosis of superficial veins |
| I636 | Cerebral infarction due to cerebral venous thrombosis, non-pyogenic |
| I676 | Nonpyogenic thrombosis of intracranial venous system |
| I822 | Embolism and thrombosis of vena cava |
| I823 | Embolism and thrombosis of renal vein |
| I829 | Embolism and thrombosis of unspecified vein |
| I800 | Phlebitis and thrombophlebitis of superficial vessels of lower extremities |
| I801 | Phlebitis and thrombophlebitis of femoral vein |
| I802 | Phlebitis and thrombophlebitis of other deep vessels of lower extremities |
| I803 | Phlebitis and thrombophlebitis of lower extremities, unspecified |
| I808 | Phlebitis and thrombophlebitis of other sites |
| I809 | Phlebitis and thrombophlebitis of unspecified site |
| I830 | Varicose veins of lower extremities with ulcer |
| I831 | Varicose veins of lower extremities with inflammation |
| I832 | Varicose veins of lower extremities with ulcer and inflammation |
| I839 | Varicose veins of lower extremities without ulcer or inflammation |
| I860 | Sublingual varices |
| I861 | Scrotal varices |
| I862 | Pelvic varices |
| I863 | Vulval varices |
| I864 | Gastric varices |
| I868 | Varicose veins of other specified sites |
| I840 | Internal thrombosed haemorrhoids |
| I841 | Internal haemorrhoids with other complications |
| I842 | Internal haemorrhoids without complication |
| I843 | External thrombosed haemorrhoids |
| I844 | External haemorrhoids with other complications |
| I845 | External haemorrhoids without complication |
| I846 | Residual haemorrhoidal skin tags |
| I847 | Unspecified thrombosed haemorrhoids |
| I848 | Unspecified haemorrhoids with other complications |
| I849 | Unspecified haemorrhoids without complication |
| I850 | Oesophageal varices with bleeding |
| I859 | Oesophageal varices without bleeding |
| I983 | Oesophageal varices with bleeding in diseases classified elsewhere |
| I870 | Post thrombotic syndrome |
| 1871 | Compression of vein |
| 1872 | Venous insufficiency (chronic, peripheral) |
| I878 | Other specified disorders of veins |
| I879 | Disorder of vein unspecified |
| I880 | Nonspecific mesenteric lymphadenitis |
| I881 | Chronic lymphadenitis, except mesenteric |
| I888 | Other nonspecific lymphadenitis |
| I889 | Nonspecific lymphadenitis, unspecified |
| I890 | Lymphodema not elsewhere classified |
| I891 | Lymphangitis |
| I898 | Other specified non infective disorders of lymphatic vessels and lymph nodes |
| I899 | Non infective disorder of lymphatic vessels and lymph nodes, unspecified |
| I740 | Embolism and thrombosis of abdominal aorta |
| I741 | Embolism and thrombosis of other and unspecified parts of aorta |
| I742 | Embolism and thrombosis of upper extremities |
| I743 | Embolism and thrombosis of lower extremities |
| I744 | Embolism and thrombosis of extremities, unspecified |
| I745 | Embolism and thrombosis of iliac artery |
| I748 | Embolism and thrombosis of other arteries |
| I749 | Embolism and thrombosis of unspecified artery |
| I770 | Arteriovenous fistula, acquired |
| I771 | Stricture of artery |
| I772 | Rupture of artery |
| I773 | Arterial fibromuscular dysplasia |
| I774 | Coeliac artery compression syndrome |
| I775 | Necrosis of artery |
| I776 | Arteritis, unspecified |
| I778 | Other specified disorders of arteries and arterioles |
| I779 | Disorder of arteries and arterioles, unspecified |
| I780 | Hereditary haemorrhagic telangiectasia |
| 1781 | Naevus non-neoplastic |
| 1788 | Other diseases of capillaries |
| 1789 | Diseases of capillaries, unspecified |
| I791 | Aortitis in diseases classified elsewhere |
| I792 | Peripheral angiopathy in diseases classified elsewhere |
| I798 | Other disorders of arteries, arterioles and capillaries in diseases classified elsewhere |
| D62 | Acute post-haemorrhagic anaemia |
| J942 | Haemothorax |
| H113 | Conjunctival haemorrhage |
| H356 | Retinal haemorrhage |
| H431 | Vitreous haemorrhage |
| N02 | Recurrent and persistent haematuria |
| R04 | Haemorrhage from respiratory passages |
| R31 | Unspecified haematuria |
| R58 | Haemorrhage, not classified elsewhere |
| K250 | Gastric ulcer acute with haemorrhage |
| K252 | Gastric ulcer acute with haemorrhage and perforation |
| K254 | Gastric ulcer chronic or unspecified with haemorrhage |
| K260 | Duodenal ulcer acute with haemorrhage |
| K262 | Duodenal ulcer acute with haemorrhage and perforation |
| K264 | Duodenal ulcer chronic or unspecified with haemorrhage |
| K270 | Peptic ulcer acute with haemorrhage |
| K272 | Peptic ulcer acute with haemorrhage and perforation |
| K274 | Peptic ulcer chronic or unspecified with haemorrhage |
| K280 | Gastrojejunal ulcer acute with haemorrhage |
| K282 | Gastrojejunal ulcer acute with haemorrhage and perforation |
| K290 | Gastrojejunal ulcer chronic or unspecified with haemorrhage |
| S063 | Focal brain injury |
| S064 | Epidural haemorrhage |
| S065 | Traumatic subdural haemorrhage |
| S066 | Traumatic subarachnoid haemorrhage |
| N950 | Postmenopausal bleeding |
| H313 | Choroidal haemorrhage and rupture |
| N93 | Other abnormal uterine and vaginal bleeding |
| R31X | Unspecified haematuria |
| N938 | Other specified abnormal uterine and vaginal bleeding |
| K921 | Melaena |
| K920 | Haematemesis |
| N939 | Abnormal uterine and vaginal bleeding, unspecified |
| K762 | Central haemorrhagic necrosis of liver |
| T810 | Haemorrhage and haematoma complicating a procedure, not elsewhere classified |
| T792 | Traumatic secondary and recurrent haemorrhage |
| N924 | Excessive bleeding in the premenopausal period |
| N421 | Congestion and haemorrhage of prostate |
| D698 | Other specified haemorrhagic conditions |
| H450 | Vitreous haemorrhage in diseases classified elsewhere |
| D683 | Haemorrhagic disorder due to circulating anticoagulants |
| K922 | Gastrointestinal haemorrhage, unspecified |
| I983 | Oesophageal varices with bleeding in diseases classified elsewhere |
| D699 | Haemorrhagic condition, unspecified |
| K762 | Central haemorrhagic necrosis of liver |
| K226 | Gastro-oesophageal laceration-haemorrhage syndrome |
| I48 | Atrial fibrillation and flutter |
| I00 | Rheumatic fever without mention of heart involvement |
| I01 | Rheumatic fever with heart involvement |
| I02 | Rheumatic chorea |
| I30 | Acute pericarditis |
| I31 | Other diseases of pericardium |
| I32 | Pericarditis in diseases classified elsewhere |
| I34 | Non-rheumatic mitral valve disorders |
| I35 | Non-rheumatic aortic valve disorders |
| I36 | Non-rheumatic tricuspid valve disorders |
| I37 | Pulmonary valve disorders |
| I05 | Rheumatic mitral valve disease |
| I06 | Rheumatic aortic valve disease |
| I07 | Rheumatic tricuspid valve disease |
| I08 | Multiple valve diseases |
| I09 | Other rheumatic heart diseases |
| I10 | Essential [primary] hypertension |
| I11 | Hypertensive heart disease |
| I12 | Hypertensive renal disease |
| I13 | Hypertensive heart and renal disease |
| I15 | Secondary hypertension |
| I33 | Acute and subacute endocarditis |
| I38 | Endocarditis valve unspecified |
| I39 | Endocarditis and heart valve disorders in diseases classified elsewhere |
| I40 | Acute myocarditis |
| I41 | Myocarditis in diseases classified elsewhere |
| I514 | Myocarditis unspecified |
| I42 | Cardiomyopathy |
| I43 | Cardiomyopathy in diseases classified elsewhere |
| I440 | Atrioventricular block, first degree |
| I441 | Atrioventricular block, second degree |
| I442 | Atrioventricular block, complete |
| I443 | Other and unspecified atrioventricular block |
| I444 | Left anterior facsicular block |
| I445 | Left posterior fascicular block |
| I446 | Other and unspecified fascicular block |
| I447 | Left bundle branch block, unspecified |
| I450 | Right fascicular block |
| I451 | Other and unspecified right bundle branch block |
| I452 | Bifascicular block |
| I453 | Trifascicular block |
| I454 | Nonspecific intraventricular block |
| I455 | Other specified heart block |
| I456 | Pre-excitation syndrome |
| I458 | Other specified conduction disorders |
| I459 | Conduction disorder, unspecified |
| I460 | Cardiac arrest with successful resuscitation |
| I461 | Sudden cardiac death, so described |
| I469 | Cardiac arrest, unspecified |
| I470 | Re-entry ventricular arrhythmia |
| I471 | Supraventricular tachycardia |
| I472 | Ventricular tachycardia |
| I479 | Paroxysmal tachycardia unspecified |
| I490 | Ventricular fibrillation or flutter |
| I491 | Atrial premature depolarisation |
| I492 | Junctional premature depolarisation |
| I493 | Ventricular premature depolarisation |
| I494 | Other and unspecified premature depolarisation |
| I495 | Sick sinus syndrome |
| I498 | Other specified cardiac arrhythmias |
| I499 | Cardiac arrhythmia unspecified |
| G454 | Transient global amnesia |
| I670 | Dissection of cerebral arteries, nonruptured |
| I671 | Cerebral aneurysm, nonruptured |
| I672 | Cerebral atherosclerosis |
| I673 | Progressive vascular leukoencephalopathy |
| I674 | Hypertensive encephalopathy |
| I675 | Moyamoya disease |
| I677 | Cerebral arteritis not elsewhere classified |
| I680 | Cerebral amyloid angiopathy |
| I681 | Cerebral arteritis in infectious and parasitic diseases classified elsewhere |
| I682 | Cerebral arteritis in other diseases classified elsewhere |
| I690 | Sequelae of subarachnoid haemorrhage |
| 1691 | Sequelae of intracerebral haemorrhage |
| 1692 | Sequelae of other non-traumatic haemorrhage |
| 1693 | Sequelae of cerebral infarction |
| 1694 | Sequelae of stroke, not specified as haemorrhage or infarction |
| 1698 | Sequelae of other and unspecified cerebrovascular diseases |
| G458 | Other transient cerebral ischaemic attacks and related syndromes |
| G459 | Transient cerebral ischaemic attack, unspecified |
| I702 | Atherosclerosis of arteries of extremities |
| I710 | Dissection of aorta any part |
| I711 | Thoracic aortic aneurysm, ruptured |
| I712 | Thoracic aortic aneurysm, without mention of rupture |
| I713 | Abdominal aortic aneurysm, ruptured |
| I714 | abdominal aortic aneurysm, without mention of rupture |
| I715 | Thoracoabdominal aortic aneurysm ruptured |
| I716 | Thoracoabdominal aortic aneurysm, without mention of rupture |
| I718 | Aortic aneurysm of unspecified site, ruptured |
| 1719 | Aortic aneurysm of unspecified site, without mention of rupture |
| I790 | Aneurysm of aorta in diseases classified elsewhere |
| I730 | Raynaud syndrome |
| I731 | Thromboangiitis obliterans |
| I738 | Other specified peripheral vascular diseases |
| I739 | Peripheral vascular disease unspecified |
| R02 | Gangrene, not elsewhere classified |
| I700 | Atherosclerosis of the aorta |
| I701 | Atherosclerosis of the renal artery |
| I708 | Atherosclerosis of other arteries |
| I709 | Generalised and unspecified atherosclerosis |
| I720 | Aneurysm and dissection of carotid artery |
| I721 | Aneurysm and dissection of artery of upper extremity |
| I722 | Aneurysm and dissection of renal artery |
| I723 | Aneurysm and dissection of iliac artery |
| I724 | Aneurysm and dissection of artery of lower extremity |
| I725 | Aneurysm and dissection of other precerebral arteries |
| I728 | Aneurysm and dissection of other specified arteries |
| I729 | Aneurysm and dissection of unspecified site |
| I200 | Unstable angina |
| I201 | Angina pectoris with documented spasm |
| I208 | Other forms of angina pectoris |
| 1209 | Angina pectoris unspecified |
| I240 | Coronary thrombosis not resulting in myocardial infarction |
| I248 | Other forms of acute ischaemic heart disease as a result of coronary failure/insufficiency |
| I249 | Acute ischaemic heart disease, unspecified |
| I250 | Atherosclerotic cardiovascular disease, so described |
| I251 | Atherosclerotic heart disease |
| I253 | Aneurysm of the heart |
| I254 | Aneurysm of the coronary artery |
| I255 | Ischaemic cardiomyopathy |
| I256 | Silent myocardial ischaemia |
| I258 | Other forms of chronic ischaemic heart disease |
| I259 | Chronic ischaemic heart disease, unspecified |
| I510 | Cardiac septal defect, acquired |
| I511 | Rupture of chordae tendineae, not elsewhere classified |
| I512 | Rupture of papillary muscle, not elsewhere classified |
| I513 | Intracardiac thrombosis, not elsewhere classified |
| I515 | Myocardial degeneration |
| I516 | Cardiovascular disease unspecified |
| I517 | Cardiomegaly |
| I518 | Other ill defined heart disease |
| I519 | Heart disease, unspecified |
| I520 | Other heart disorders in bacterial diseases classified elsewhere, |
| I521 | Other heart disorders in other infectious and parasitic diseases classified elsewhere |
| I528 | Other heart disorders in other diseases classified elsewhere |
| I950 | Idiopathic hypotension |
| I951 | Orthostatic hypotension |
| I952 | Hypotension due to drugs |
| I958 | Other hypotension |
| I959 | Hypotension, unspecified |
| I970 | Postcardiotomy syndrome |
| I971 | Other function disturbances following cardiac surgery |
| I972 | Postmastectomy lymphedema syndrome |
| I978 | Other post procedural disorders of circulatory system not elsewhere classified |
| I979 | Postprocedural disorder of circulator system, unspecified |
| I980 | Cardiovascular syphilis |
| I981 | Cardiovascular disorders in other infectious and parasitic diseases classified elsewhere |
| I982 | Oesophageal varices without bleeding in diseases classified elsewhere |
| I983 | Oesophageal varices without bleeding in diseases classified elsewhere |
| I988 | Other specified disorders of circulatory system in diseases classified elsewhere |
| I99 | Other and unspecified disorders of circulatory system |
| I600 | Subarachnoid haemorrhage from carotid siphon and bifurcation |
| I601 | Subarachnoid haemorrhage from middle cerebral artery |
| I602 | Subarachnoid haemorrhage from anterior communicating artery |
| I603 | Subarachnoid haemorrhage from posterior communicating artery |
| I604 | Subarachnoid haemorrhage from basilar artery |
| I605 | Subarachnoid haemorrhage from vertebral artery |
| I606 | Subarachnoid haemorrhage from other intracranial arteries |
| I607 | Subarachnoid haemorrhage from intracranial arteryunspec |
| I608 | Other subarachnoid haemorrhage |
| I609 | Subarachnoid haemorrhageunspecified |
| I610 | Intracerebral haemorrhage in hemispheresubcortical |
| I611 | Intracerebral haemorrhage in hemispherecortical |
| I612 | Intracerebral haemorrhage in hemisphereunspecified |
| I613 | Intracerebral haemorrhage in brain stem |
| I614 | Intracerebral haemorrhage in cerebellum |
| I615 | Intracerebral haemorrhageintraventricular |
| I616 | Intracerebral haemorrhagemultiple localized |
| I618 | Other intracerebral haemorrhage |
| I619 | Intracerebral haemorrhageunspecified |
| I620 | Subdural haemorrhage (acute)(nontraumatic) |
| I621 | Nontraumatic extradural haemorrhage, Nontraumatic epidural haemorrhage |
| I629 | Intracranial haemorrhage (nontraumatic)unspecified |
| I630 | Cerebral infarct due to thrombosis of precerebral arteries |
| I631 | Cerebral infarction due to embolism of precerebral arteries |
| I632 | Cereb infarct due unsp occlusion or stenos precerebrl arts |
| I633 | Cerebral infarction due to thrombosis of cerebral arteries |
| I634 | Cerebral infarction due to embolism of cerebral arteries |
| I635 | Cerebrl infarct due unspec occlusion or stenos cerebrl arts |
| I636 | Cereb infarct due cerebral venous thrombosisnonpyogenic |
| I638 | Other cerebral infarction |
| I639 | Cerebral infarctionunspecified |
| G460 | Middle cerebral artery syndrome |
| G461 | Anterior cerebral artery syndrome |
| G462 | Posterior cerebral artery syndrome |
| G463 | Brain stem stroke syndrome |
| G464 | Cerebellar stroke syndrome |
| G465 | Pure motor lacunar stroke syndrome |
| G466 | Pure sensory lacunar syndrome |
| G467 | Other lacunar syndromes |
| G468 | Other vascular syndromes of brain in cerebrovascular diseases |
| G450 | Vertebro-basilar artery syndrome |
| G451 | Carotid artery syndrome (hemispheric) |
| G452 | Multiple and bilateral precerebral artery syndromes |
| I64X | Stroke not specified as haemorrhage or infarction |
| I678 | Other specified cerebrovascular diseases |
| I679 | Cerebrovascular disease, unspecified |
| I688 | Other cerebrovascular disorders in diseases classified elsewhere |
| I650 | Occlusion and stenosis of vertebral artery |
| I651 | Occlusion and stenosis of basilaR artery |
| I652 | Occlusion and stenosis of carotid artery |
| I653 | Occlusion and stenosis of multiple and bilateral precerebral arteries |
| I658 | Occlusion and stenosis of unspecified precerebral artery |
| I659 | Occlusion and stenosis of unspecified precerebral artery |
| I660 | Occlusion and stenosis of middle cerebral artery |
| I661 | Occlusion and stenosis of anterior cerebral artery |
| I662 | Occlusion and stenosis of posterior cerebral artery |
| I663 | Occlusion and stenosis of cerebellar arteries |
| I664 | Occlusion and stenosis of multiple and bilateral cerebral arteries |
| I668 | Occlusion and stenosis of other cerebral artery |
| I669 | Occlusion and stenosis of unspecified cerebral artery |
| **Myocardial infarction** | |
| I210 | Acute transmural myocardial infarction of anterior wall |
| I211 | Acute transmural myocardial infarction of inferior wall |
| I212 | Acute transmural myocardial infarction of other sites |
| I213 | Acute transmural myocardial infarction of unspecified site |
| I214 | Acute subendocardial myocardial infarction |
| I219 | Acute myocardial infarction unspecified |
| I220 | Subsequent myocardial infarction of anterior wall |
| I221 | Subsequent myocardial infarction of inferior wall |
| I228 | Subsequent myocardial infarction of other sites |
| I229 | Subsequent myocardial infarction of unspecified site |
| I230 | Haemopericardium as current complication following acute myocardial infarction |
| I231 | Atrial septal defect as current complication following acute myocardial infarction |
| I232 | Ventricular septal defect as current complication following acute myocardial infarction |
| I233 | Rupture of cardiac wall without haemopericardium as current complication following acute myocardial infarction |
| I234 | Rupture of chordae tendineae as current complication following acute myocardial infarction |
| I235 | Rupture of papillary muscle as current complication following acute myocardial infarction |
| I236 | Thrombosis of atrium, auricular appendage, and ventricle as current complications following acute myocardial infarction |
| I238 | Other current complications following acute myocardial infarction |
| I252 | Old myocardial infarction |
| I241 | Dressler syndrome (post myocardial infarction syndrome) |
| **Heart failure** | |
| I50 | Congestive heart failure |
| I110 | Left ventricular failure |
| I130 | Hypertensive heart disease with congestive heart failure |
| I132 | Hypertensive heart and renal disease with congestive heart failure and renal failure |
| I420 | Dilated cardiomyopathy |
| **Coronary artery disease** | |
| I210 | acute transmural myocardial infarction of anterior wall |
| I211 | acute transmural myocardial infarction of inferior wall |
| I212 | acute transmural myocardial infarction of other sites |
| I213 | acute transmural myocardial infarction of unspecified site |
| I214 | acute subendocardial myocardial infarction |
| I219 | acute myocardial infarction unspecified |
| I220 | subsequent myocardial infarction of anterior wall |
| I221 | subsequent myocardial infarction of inferior wall |
| I228 | subsequent myocardial infarction of other sites |
| I229 | subsequent myocardial infarction of unspecified site |
| I230 | haemopericardium as current complication following acute myocardial infarction |
| I231 | atrial septal defect as current complication following acute myocardial infarction |
| I232 | ventricular septal defect as current complication following acute myocardial infarction |
| I233 | rupture of cardiac wall without haemopericardium as current complication following acute myocardial infarction |
| I234 | rupture of chordae tendineae as current complication following acute myocardial infarction |
| I235 | rupture of papillary muscle as current complication following acute myocardial infarction |
| I236 | thrombosis of atrium, auricular appendage, and ventricle as current complications following acute myocardial infarction |
| I238 | other current complications following acute myocardial infarction |
| I252 | old myocardial infarction |
| I241 | dressler syndrome (post myocardial infarction syndrome) |
| I200 | unstable angina |
| I201 | angina pectoris with documented spasm |
| I208 | other forms of angina pectoris |
| 1209 | angina pectoris unspecified |
| I240 | coronary thrombosis not resulting in myocardial infarction |
| I248 | other forms of acute ischaemic heart disease as a result of coronary failure/insufficiency |
| I249 | acute ischaemic heart disease, unspecified |
| I250 | atherosclerotic cardiovascular disease, so described |
| I251 | atherosclerotic heart disease |
| I254 | aneurysm of the coronary artery |
| I255 | ischaemic cardiomyopathy |
| I256 | silent myocardial ischaemia |
| I258 | other forms of chronic ischaemic heart disease |
| I259 | chronic ischaemic heart disease, unspecified |
| **Venous thromboembolism** | |
| I260 | Pulmonary embolism with mention of acute cor pulmonale |
| I269 | Pulmonary embolism without mention of acute cor pulmonale |
| I636 | Cerebral infarction due to cerebral venous thrombosis, non-pyogenic |
| I676 | Nonpyogenic thrombosis of intracranial venous system |
| I822 | Embolism and thrombosis of vena cava |
| I823 | Embolism and thrombosis of renal vein |
| I829 | Embolism and thrombosis of unspecified vein |
| **Major bleeding** | |
| D62 | Acute post-haemorrhagic anaemia |
| J942 | Haemothorax |
| H113 | Conjunctival haemorrhage |
| H356 | Retinal haemorrhage |
| H431 | Vitreous haemorrhage |
| N02 | Recurrent and persistent haematuria |
| R04 | Haemorrhage from respiratory passages |
| R31 | Unspecified haematuria |
| R58 | Haemorrhage, not classified elsewhere |
| K250 | Gastric ulcer acute with haemorrhage |
| K252 | Gastric ulcer acute with haemorrhage and perforation |
| K254 | Gastric ulcer chronic or unspecified with haemorrhage |
| K260 | Duodenal ulcer acute with haemorrhage |
| K262 | Duodenal ulcer acute with haemorrhage and perforation |
| K264 | Duodenal ulcer chronic or unspecified with haemorrhage |
| K270 | Peptic ulcer acute with haemorrhage |
| K272 | Peptic ulcer acute with haemorrhage and perforation |
| K274 | Peptic ulcer chronic or unspecified with haemorrhage |
| K280 | Gastrojejunal ulcer acute with haemorrhage |
| K282 | Gastrojejunal ulcer acute with haemorrhage and perforation |
| K290 | Gastrojejunal ulcer chronic or unspecified with haemorrhage |
| S063 | Focal brain injury |
| S064 | Epidural haemorrhage |
| S065 | Traumatic subdural haemorrhage |
| S066 | Traumatic subarachnoid haemorrhage |
| N950 | Postmenopausal bleeding |
| H313 | Choroidal haemorrhage and rupture |
| N93 | Other abnormal uterine and vaginal bleeding |
| R31X | Unspecified haematuria |
| N938 | Other specified abnormal uterine and vaginal bleeding |
| K921 | Melaena |
| K920 | Haematemesis |
| N939 | Abnormal uterine and vaginal bleeding, unspecified |
| K762 | Central haemorrhagic necrosis of liver |
| T810 | Haemorrhage and haematoma complicating a procedure, not elsewhere classified |
| T792 | Traumatic secondary and recurrent haemorrhage |
| N924 | Excessive bleeding in the premenopausal period |
| N421 | Congestion and haemorrhage of prostate |
| D698 | Other specified haemorrhagic conditions |
| H450 | Vitreous haemorrhage in diseases classified elsewhere |
| D683 | Haemorrhagic disorder due to circulating anticoagulants |
| K922 | Gastrointestinal haemorrhage, unspecified |
| I983 | Oesophageal varices with bleeding in diseases classified elsewhere |
| D699 | Haemorrhagic condition, unspecified |
| K762 | Central haemorrhagic necrosis of liver |
| K226 | Gastro-oesophageal laceration-haemorrhage syndrome |
| **Atrial fibrillation** | |
| I48 | Atrial fibrillation and flutter |
| **Hypertension** | |
| I10 | Essential [primary] hypertension |
| I11 | Hypertensive heart disease |
| I12 | Hypertensive renal disease |
| I13 | Hypertensive heart and renal disease |
| I15 | Secondary hypertension |
| **Transient ischaemic attack** | |
| G458 | Other transient cerebral ischaemic attacks and related syndromes |
| G459 | Transient cerebral ischaemic attack, unspecified |
| **Peripheral vascular disease** | |
| I702 | Atherosclerosis of arteries of extremities |
| I710 | Dissection of aorta any part |
| I711 | Thoracic aortic aneurysm, ruptured |
| I712 | Thoracic aortic aneurysm, without mention of rupture |
| I713 | Abdominal aortic aneurysm, ruptured |
| I714 | Abdominal aortic aneurysm, without mention of rupture |
| I715 | Thoracoabdominal aortic aneurysm ruptured |
| I716 | Thoracoabdominal aortic aneurysm, without mention of rupture |
| I718 | Aortic aneurysm of unspecified site, ruptured |
| 1719 | Aortic aneurysm of unspecified site, without mention of rupture |
| I790 | Aneurysm of aorta in diseases classified elsewhere |
| **Aortic plaque** | |
| I700 | Atherosclerosis of the aorta |
| **Connective tissue disease** | |
| M05 | Felty syndrome |
| M060 | Seronegative rheumatoid arthritis |
| M063 | Rheumatoid nodule |
| M069 | Rheumatoid arthritis, unspecified |
| M32 | Systemic lupus erythematosus |
| M332 | Polymyositis |
| M34 | Systemic sclerosis |
| M353 | Polymyalgia rheumatica |
| Alzheimer’s disease | |
| F00 | Dementia in Alzheimer disease |
| Vascular dementia | |
| F01 | Vascular dementia |
| **Other dementia or unspecified** | |
| F02 | Dementia in other diseases classified elsewhere |
| F03 | Unspecified dementia |
| F051 | Delirium superimposed on dementia |
| **Diabetes mellitus** | |
| E10 | Insulin-dependent diabetes mellitus |
| E11 | Non-insulin-dependent diabetes mellitus |
| E12 | Malnutrition-related diabetes mellitus |
| E13 | Other specified diabetes mellitus |
| E14 | Unspecified diabetes mellitus |
| **Peptic ulcer** | |
| K25 | Gastric ulcer |
| K26 | Duodenal ulcer |
| K27 | Peptic ulcer, site unspecified |
| K28 | Gastrojejunal ulcer |
| **Renal disease** | |
| I12 | Hypertensive renal disease |
| I13 | Hypertensive heart and renal disease |
| N01 | Rapidly progressive nephritic syndrome |
| N03 | Chronic nephritic syndrome |
| N18 | Chronic kidney disease |
| N19 | Unspecified kidney failure |
| N25 | Disorders resulting from impaired renal tubular function |
| N00 | Acute nephritic syndrome |
| N04 | Nephrotic syndrome |
| N05 | Unspecified nephritic syndrome |
| N07 | Hereditary nephropathy, not elsewhere classified |
| N11 | Chronic tubulo-interstitial nephritis |
| N14 | Drug- and heavy-metal-induced tubulo-interstitial and tubular conditions |
| N17 | Acute renal failure |
| Q61 | Cystic kidney disease |
| **Alcoholism** | |
| E244 | Alcohol-induced pseudo-Cushing syndrome |
| F10 | Mental and behavioural disorders due to use of alcohol |
| G312 | Degeneration of nervous system due to alcohol |
| G621 | Alcoholic polyneuropathy |
| G721 | Alcoholic myopathy |
| I426 | Alcoholic cardiomyopathy |
| K292 | Alcoholic gastritis |
| K70 | Alcoholic liver disease |
| K860 | Alcohol-induced chronic pancreatitis |
| O354 | Maternal care for (suspected) damage to fetus from alcohol |
| T51 | Toxic effect of alcohol |
| Z714 | Alcohol abuse counselling and surveillance |
| Z721 | Alcohol use |
| **Chronic obstructive pulmonary sisease** | |
| J40 | Bronchitis, not specified as acute or chronic |
| J41 | Simple and mucopurulent chronic bronchitis |
| J42 | Unspecified chronic bronchitis |
| J43 | Emphysema |
| J44 | Other chronic obstructive pulmonary disease |
| J47 | Bronchiectasis |
| **Asthma** | |
| J45 | Asthma |
| J46 | Status asthmaticus |
| **Other pulmonary disease** | |
| J60 | Coalworker pneumoconiosis |
| J61 | Pneumoconiosis due to asbestos and other mineral fibres |
| J62 | Pneumoconiosis due to dust containing silica |
| J63 | Pneumoconiosis due to other inorganic dusts |
| J64 | Unspecified pneumoconiosis |
| J65 | Pneumoconiosis associated with tuberculosis |
| J66 | Airway disease due to specific organic dust |
| J67 | Hypersensitivity pneumonitis due to organic dust |
| **Liver disease** | |
| K721 | Chronic hepatic failure |
| K729 | Hepatic failure, unspecified |
| K766 | Portal hypertension |
| K767 | Hepatorenal syndrome |
| B150 | Hepatitis A with hepatic coma |
| B160 | Acute hepatitis B with delta-agent (coinfection) with hepatic coma |
| B162 | Acute hepatitis B without delta-agent with hepatic coma |
| B190 | Unspecified viral hepatitis with hepatic coma |
| K704 | Alcoholic hepatic failure |
| K72 | Acute and subacute hepatic failure |
| I85 | Oesophageal varices |
| I983 | Oesophageal varices with bleeding in diseases classified elsewhere |
| K702 | Alcoholic fibrosis and sclerosis of liver |
| K703 | Alcoholic cirrhosis of liver |
| K717 | Toxic liver disease with fibrosis and cirrhosis of liver |
| K73 | Chronic hepatitis, not elsewhere classified |
| K74 | Fibrosis and cirrhosis of liver |
| K701 | Alcoholic hepatitis |
| K754 | Autoimmune hepatitis |
| **Cancer** | |
| C00 | Malignant neoplasm of lip |
| C01 | Malignant neoplasm of base of tongue |
| C02 | Malignant neoplasm of other and unspecified parts of tongue |
| C03 | Malignant neoplasm of gum |
| C04 | Malignant neoplasm of floor of mouth |
| C05 | Malignant neoplasm of palate |
| C06 | Malignant neoplasm of other and unspecified parts of mouth |
| C07 | Malignant neoplasm of parotid gland |
| C08 | Malignant neoplasm of other and unspecified major salivary glands |
| C09 | Malignant neoplasm of tonsil |
| C10 | Malignant neoplasm of oropharynx |
| C11 | Malignant neoplasm of nasopharynx |
| C12 | Malignant neoplasm of piriform sinus |
| C13 | Malignant neoplasm of hypopharynx |
| C14 | Malignant neoplasm of other and ill-defined sites in the lip, oral cavity and pharynx |
| C15 | Cervical part of oesophagus |
| C16 | Malignant neoplasm of stomach |
| C17 | Malignant neoplasm of small intestine |
| C18 | Malignant neoplasm of colon |
| C19 | Malignant neoplasm of rectosigmoid junction |
| C20 | Malignant neoplasm of rectum |
| C21 | Malignant neoplasm of anus and anal canal |
| C22 | Malignant neoplasm of liver and intrahepatic bile ducts |
| C23 | Malignant neoplasm of gallbladder |
| C24 | Malignant neoplasm of other and unspecified parts of biliary tract |
| C25 | Malignant neoplasm of pancreas |
| C26 | Malignant neoplasm of other and ill-defined digestive organs |
| C30 | Malignant neoplasm of nasal cavity and middle ear |
| C31 | Malignant neoplasm of accessory sinuses |
| C32 | Malignant neoplasm of larynx |
| C33 | Malignant neoplasm of trachea |
| C34 | Malignant neoplasm of bronchus and lung |
| C37 | Malignant neoplasm of thymus |
| C38 | Malignant neoplasm of heart, mediastinum and pleura |
| C39 | Malignant neoplasm of other and ill-defined sites in the respiratory system and intrathoracic organs |
| C40 | Malignant neoplasm of bone and articular cartilage of limbs |
| C41 | Malignant neoplasm of bone and articular cartilage of other and unspecified sites |
| C43 | Malignant melanoma of skin |
| C44 | Other malignant neoplasms of skin |
| C45 | Mesothelioma |
| C46 | Kaposi sarcoma |
| C47 | Malignant neoplasm of peripheral nerves and autonomic nervous system |
| C48 | Malignant neoplasm of retroperitoneum and peritoneum |
| C49 | Malignant neoplasm of other connective and soft tissue |
| C50 | Malignant neoplasm of breast |
| C51 | Malignant neoplasm of vulva |
| C52 | Malignant neoplasm of vagina |
| C53 | Malignant neoplasm of cervix uteri |
| C54 | Malignant neoplasm of corpus uteri |
| C55 | Malignant neoplasm of uterus, part unspecified |
| C56 | Malignant neoplasm of ovary |
| C57 | Malignant neoplasm of other and unspecified female genital organs |
| C58 | Malignant neoplasm of placenta |
| C60 | Malignant neoplasm of penis |
| C61 | Malignant neoplasm of prostate |
| C62 | Malignant neoplasm of testis |
| C63 | Malignant neoplasm of other and unspecified male genital organs |
| C64 | Malignant neoplasm of kidney, except renal pelvis |
| C65 | Malignant neoplasm of renal pelvis |
| C66 | Malignant neoplasm of ureter |
| C67 | Malignant neoplasm of bladder |
| C68 | Malignant neoplasm of other and unspecified urinary organs |
| C69 | Malignant neoplasm of eye and adnexa |
| C70 | Malignant neoplasm of meninges |
| C71 | Malignant neoplasm of brain |
| C72 | Malignant neoplasm of spinal cord, cranial nerves and other parts of central nervous system |
| C73 | Malignant neoplasm of thyroid gland |
| C74 | Malignant neoplasm of adrenal gland |
| C75 | Malignant neoplasm of other endocrine glands and related structures |
| C76 | Malignant neoplasm of other and ill-defined sites |
| C80 | Malignant neoplasm, without specification of site |
| C81 | Hodgkin lymphoma |
| C82 | Follicular lymphoma |
| C83 | Non-follicular lymphoma |
| C84 | Mature T/NK-cell lymphomas |
| C85 | Other and unspecified types of non-Hodgkin lymphoma |
| C86 | Other specified types of T/NK-cell lymphoma |
| C88 | Malignant immunoproliferative diseases |
| C90 | Multiple myeloma and malignant plasma cell neoplasms |
| C91 | Lymphoid leukaemia |
| C92 | Myeloid leukaemia |
| C93 | Monocytic leukaemia |
| C94 | Other leukaemias of specified cell type |
| C95 | Leukaemia of unspecified cell type |
| C96 | Other and unspecified malignant neoplasms of lymphoid, haematopoietic and related tissue |
| C97 | Malignant neoplasms of independent (primary) multiple sites |
| C77 | Secondary and unspecified malignant neoplasm of lymph nodes |
| C78 | Secondary malignant neoplasm of respiratory and digestive organs |
| C79 | Secondary malignant neoplasm of other and unspecified sites |
| **Haemorrhagic stroke** | |
| I600 | Subarachnoid haemorrhage from carotid siphon and bifurcation |
| I601 | Subarachnoid haemorrhage from middle cerebral artery |
| I602 | Subarachnoid haemorrhage from anterior communicating artery |
| I603 | Subarachnoid haemorrhage from posterior communicating artery |
| I604 | Subarachnoid haemorrhage from basilar artery |
| I605 | Subarachnoid haemorrhage from vertebral artery |
| I606 | Subarachnoid haemorrhage from other intracranial arteries |
| I607 | Subarachnoid haemorrhage from intracranial artery unspecified |
| I608 | Other subarachnoid haemorrhage |
| I609 | Subarachnoid haemorrhage unspecified |
| I610 | Intracerebral haemorrhage in hemisphere subcortical |
| I611 | Intracerebral haemorrhage in hemisphere cortical |
| I612 | Intracerebral haemorrhage in hemisphere unspecified |
| I613 | Intracerebral haemorrhage in brain stem |
| I614 | Intracerebral haemorrhage in cerebellum |
| I615 | Intracerebral haemorrhage intraventricular |
| I616 | Intracerebral haemorrhage multiple localized |
| I618 | Other intracerebral haemorrhage |
| I619 | Intracerebral haemorrhage unspecified |
| I620 | Subdural haemorrhage (acute)(nontraumatic) |
| I621 | Nontraumatic extradural haemorrhage, Nontraumatic epidural haemorrhage |
| I629 | Intracranial haemorrhage (nontraumatic)unspecified |
| **Ischaemic stroke** | |
| I630 | Cerebral infarct due to thrombosis of precerebral arteries |
| I631 | Cerebral infarction due to embolism of precerebral arteries |
| I632 | Cerebral infarction due to unspecified occlusion or stenosis of precerebral arteries |
| I633 | Cerebral infarction due to thrombosis of cerebral arteries |
| I634 | Cerebral infarction due to embolism of cerebral arteries |
| I635 | Cerebral infarction due to unspecified occlusion or stenosis of cerebral arteries |
| I636 | Cerebral infarction due to cerebral venous thrombosis, nonpyogenic |
| I638 | Other cerebral infarction |
| I639 | Cerebral infarction unspecified |
| G460 | Middle cerebral artery syndrome |
| G461 | Anterior cerebral artery syndrome |
| G462 | Posterior cerebral artery syndrome |
| G463 | Brain stem stroke syndrome |
| G464 | Cerebellar stroke syndrome |
| G465 | Pure motor lacunar stroke syndrome |
| G466 | Pure sensory lacunar syndrome |
| G467 | Other lacunar syndromes |
| G468 | Other vascular syndromes of brain in cerebrovascular diseases |
| G450 | Vertebro-basilar artery syndrome |
| G451 | Carotid artery syndrome (hemispheric) |
| G452 | Multiple and bilateral precerebral artery syndromes |
| **Stroke of unspecified origin** | |
| I64X | Stroke not specified as haemorrhage or infarction |
| I678 | Other specified cerebrovascular diseases |
| I679 | Cerebrovascular disease, unspecified |
| I688 | Other cerebrovascular disorders in diseases classified elsewhere |

# Supplementary Table 2. Read codes version 2 to identify conditions/medications in the Welsh Longitudinal General Practice (WLGP) data source.

| **Read code** | **Read code definition** |
| --- | --- |
| **Haemorrhagic stroke** | |
| G60..00 | Subarachnoid haemorrhage |
| G61z.00 | Intracerebral haemorrhage not otherwise specified |
| G621.00 | Subdural haemorrhage - nontraumatic |
| G61..00 | Intracerebral haemorrhage |
| G614.00 | Pontine haemorrhage |
| G604.00 | Subarachnoid haemorrhage from posterior communicating artery |
| G613.00 | Cerebellar haemorrhage |
| G60X.00 | Subarachnoid haemorrhage from intracranial artery, unspecified |
| G622.00 | Subdural haematoma - nontraumatic |
| G623.00 | Subdural haemorrhage not otherwise specified |
| G61X100 | Right sided intracerebral haemorrhage, unspecified |
| G602.00 | Subarachnoid haemorrhage from middle cerebral artery |
| G60z.00 | Subarachnoid haemorrhage not otherwise specified |
| G61X000 | Left sided intracerebral haemorrhage, unspecified |
| G600.00 | Ruptured berry aneurysm |
| G616.00 | External capsule haemorrhage |
| G617.00 | Intracerebral haemorrhage, intraventricular |
| G61X.00 | Intracerebral haemorrhage in hemisphere, unspecified |
| G610.00 | Cortical haemorrhage |
| G620.00 | Extradural haemorrhage - nontraumatic |
| G611.00 | Internal capsule haemorrhage |
| G605.00 | Subarachnoid haemorrhage from basilar artery |
| G603.00 | Subarachnoid haemorrhage from anterior communicating artery |
| G612.00 | Basal nucleus haemorrhage |
| G601.00 | Subarachnoid haemorrhage from carotid siphon and bifurcation |
| G618.00 | Intracerebral haemorrhage, multiple localized |
| G606.00 | Subarachnoid haemorrhage from vertebral artery |
| G615.00 | Bulbar haemorrhage |
| G61..11 | CVA - cerebrovascular accident due to intracerebral haemorrhage |
| G62z.00 | Intracranial haemorrhage not otherwise specified |
| G61..12 | Stroke due to intracerebral haemorrhage |
| G62..00 | Other and unspecified intracranial haemorrhage |
| G619. | Lobar cerebral haemorrhage |
| **Ischaemic stroke** | |
| G64z.00 | Cerebral infarction not otherwise specified |
| G64..00 | Cerebral arterial occlusion |
| G64z200 | Left sided cerebral infarction |
| G64z300 | Right sided cerebral infarction |
| G641.00 | Cerebral embolism |
| G640.00 | Cerebral thrombosis |
| G64z000 | Brainstem infarction |
| G64z400 | Infarction of basal ganglia |
| G641000 | Cerebral infarction due to embolism of cerebral arteries |
| G640000 | Cerebral infarction due to thrombosis of cerebral arteries |
| G64..12 | Infarction - cerebral |
| G64..11 | CVA - cerebral artery occlusion |
| G64z.12 | Cerebellar infarction |
| G64..13 | Stroke due to cerebral arterial occlusion |
| G64z.11 | Brainstem infarction not otherwise specified |
| G63y000 | Cerebral infarct due to thrombosis of precerebral arteries |
| G63y100 | Cerebral infarction due to embolism of precerebral arteries |
| G6X..00 | Cerebral infarction due to unspecified occlusion or stenosis of cerebral arteries |
| G641.11 | Cerebral embolus |
| G676000 | Cerebral infarction due to cerebral venous thrombosis, nonpyogenic |
| G6W..00 | Cerebral infarction due to unspecified occlusion or stenosis of precerebral arteries |
| G63..11 | Infarction – precerebral |
| G64z1 | Wallenberg syndrome |
| G663.00 | Brain stem stroke syndrome |
| G664.00 | Cerebellar stroke syndrome |
| G661. | Anterior cerebral artery syndrome |
| G662 | Posterior cerebral artery syndrome |
| G665. | Pure motor lacunar syndrome |
| G666. | Pure sensory lacunar syndrome |
| **Stroke of unspecified origin** | |
| G66..11 | CVA unspecified |
| G66..13 | CVA - Cerebrovascular accident unspecified |
| G667.00 | Left sided CVA |
| G668.00 | Right sided CVA |
| G66..00 | Stroke and cerebrovascular accident unspecified |
| G66..12 | Stroke unspecified |
| 13YA.00 | Stroke group member |
| G6...00 | Cerebrovascular disease |
| G6z..00 | Cerebrovascular disease not otherwise specified |
| G67..00 | Other cerebrovascular disease |
| 8HBJ.00 | Stroke / transient ischaemic attack referral |
| L440.12 | Stroke in the puerperium |
| 14A7. | H/O: CVA/stroke |
| **Hypertension** | |
| 14A2. | H/O: hypertension |
| G2... | Hypertensive disease |
| G20.. | Essential hypertension |
| G200. | Malignant essential hypertension |
| G201. | Benign essential hypertension |
| G202. | Systolic hypertension |
| G203. | Diastolic hypertension |
| G20z. | Essential hypertension not otherwise specified |
| G21.. | Hypertensive heart disease |
| G210. | Malignant hypertension heart disease |
| G2100 | Malignant hypertension heart disease-no congestive cardiac failure |
| G2101 | Malignant hypertension heart disease+- congestive cardiac failure |
| G210z | Malignant hypertension heart disease Not otherwise specified |
| G211. | Benign hypertensive heart disease |
| G2110 | Benign hypertensive heart disease - congestive cardiac failure |
| G2111 | Benign hypertensive heart disease +- congestive cardiac failure |
| G211z | Benign hypertension heart disease Not otherwise specified |
| G21z. | Hypertensive heart disease Not otherwise specified |
| G21z0 | Hypertensive heart disease Not otherwise specified-no congestive cardiac failure |
| G21z1 | Hypertensive heart disease Not otherwise specified- +congestive cardiac failure |
| G21zz | Hypertensive heart disease Not otherwise specified |
| G22.. | Hypertensive renal disease |
| G220. | Malignant hypertensive renal disease |
| G221. | Benign hypertensive renal disease |
| G222. | Hypertensive renal disease +renal failure |
| G22z. | Hypertensive renal disease Not otherwise specified |
| G23.. | Hypertensive heart +renal disease |
| G230. | Malignant hypertensive heart +renal disease |
| G231. | Benign hypertensive heart +renal disease |
| G232. | Hypertensive heart+ renal disease +heart failure |
| G233. | Hypertensive heart+ renal disease +renal fail |
| G234. | Hypertensive heart+ renal disease +both heart + renal failure |
| G23z. | Hypertensive heart+ renal disease not otherwise specified |
| G24.. | Secondary hypertension |
| G240. | Secondary malignant hypertension |
| G2400 | Secondary malign renovascular hypertension |
| G240z | Secondary malign hypertension not otherwise specified |
| G241. | Secondary benign hypertension |
| G2410 | Secondary benign renovascular hypertension |
| G241z | Secondary benign hypertension not otherwise specified |
| G244. | Hypertension secondary endocrine disorder |
| G24z. | Secondary hypertension not otherwise specified |
| G24z0 | Secondary renovascular hypertension not otherwise specified |
| G24z1 | Hypertension secondary to drug |
| G24zz | Secondary hypertension not otherwise specified |
| G25.. | Stage 1 hypertension (NICE 2011) |
| G250. | Stage 1 hypertension without end organ damage |
| G251. | Stage 1 hypertension with end organ damage |
| G26.. | Severe hypertension (NICE 2011) |
| G27.. | Hypertension resistant to drug therapy |
| G28.. | Stage 2 hypertension (NICE 2011) |
| G2y.. | Hypertensive disease otherwise specified |
| G2z.. | Hypertensive disease not otherwise specified |
| **Atrial fibrillation** | |
| 14AN. | H/O: atrial fibrillation |
| 14AR. | History of atrial flutter |
| 3272. | ECG: atrial fibrillation |
| 3273. | ECG: atrial flutter |
| 8CMW2 | Atrial fibrillation care pathway |
| G573. | Atrial fibrillation/flutter |
| G5730 | Atrial fibrillation |
| G5731 | Atrial flutter |
| G5732 | Paroxysmal atrial fibrillation |
| G5733 | Non-rheumatic atrial fibrillation |
| G5734 | Permanent atrial fibrillation |
| G5735 | Persistent atrial fibrillation |
| G5736 | Paroxysmal atrial flutter |
| G5737 | Chronic atrial fibrillation |
| G5738 | Typical atrial flutter |
| G5739 | Atypical atrial flutter |
| G573z | Atrial fibrillation/flutter Not otherwise specified |
| **Diabetes mellitus** | |
| C100112 | Non-insulin dependent diabetes mellitus |
| C10..00 | Diabetes mellitus |
| C10F.00 | Type 2 diabetes mellitus |
| C100011 | Insulin dependent diabetes mellitus |
| C10FJ00 | Insulin treated Type 2 diabetes mellitus |
| C10E.00 | Type 1 diabetes mellitus |
| C108.00 | Insulin dependent diabetes mellitus |
| C101.00 | Diabetes mellitus with ketoacidosis |
| C104.11 | Diabetic nephropathy |
| C109.00 | Non-insulin dependent diabetes mellitus |
| C109.11 | NIDDM - Non-insulin dependent diabetes mellitus |
| C108700 | Insulin dependent diabetes mellitus with retinopathy |
| C108800 | Insulin dependent diabetes mellitus - poor control |
| C106.12 | Diabetes mellitus with neuropathy |
| C109700 | Non-insulin dependent diabetes mellitus - poor control |
| C10yy00 | Other specified diabetes mellitus with other spec comps |
| C10ED00 | Type 1 diabetes mellitus with nephropathy |
| C10EM00 | Type 1 diabetes mellitus with ketoacidosis |
| C10B.00 | Diabetes mellitus induced by steroids |
| C10E.11 | Type I diabetes mellitus |
| C10FC00 | Type 2 diabetes mellitus with nephropathy |
| C10F500 | Type 2 diabetes mellitus with gangrene |
| C104y00 | Other specified diabetes mellitus with renal complications |
| C100100 | Diabetes mellitus, adult onset, no mention of complication |
| C100111 | Maturity onset diabetes |
| C103.00 | Diabetes mellitus with ketoacidotic coma |
| C106.00 | Diabetes mellitus with neurological manifestation |
| C106.13 | Diabetes mellitus with polyneuropathy |
| C104.00 | Diabetes mellitus with renal manifestation |
| C109600 | Non-insulin-dependent diabetes mellitus with retinopathy |
| C108F11 | Type I diabetes mellitus with diabetic cataract |
| C108.12 | Type 1 diabetes mellitus |
| C109.12 | Type 2 diabetes mellitus |
| C109G11 | Type II diabetes mellitus with arthropathy |
| C109012 | Type 2 diabetes mellitus with renal complications |
| C109.13 | Type II diabetes mellitus |
| C108J12 | Type 1 diabetes mellitus with neuropathic arthropathy |
| C109J12 | Insulin treated Type II diabetes mellitus |
| C109J00 | Insulin treated Type 2 diabetes mellitus |
| C10E700 | Type 1 diabetes mellitus with retinopathy |
| C10FM00 | Type 2 diabetes mellitus with persistent microalbuminuria |
| C10FB00 | Type 2 diabetes mellitus with polyneuropathy |
| C10F600 | Type 2 diabetes mellitus with retinopathy |
| C108.11 | IDDM-Insulin dependent diabetes mellitus |
| C10EH00 | Type 1 diabetes mellitus with arthropathy |
| C10E500 | Type 1 diabetes mellitus with ulcer |
| C10F000 | Type 2 diabetes mellitus with renal complications |
| 9OLA.11 | Diabetes monitored |
| C102.00 | Diabetes mellitus with hyperosmolar coma |
| C108012 | Type 1 diabetes mellitus with renal complications |
| C10N.00 | Secondary diabetes mellitus |
| C106z00 | Diabetes mellitus not otherwise specified with neurological manifestation |
| C10EP00 | Type 1 diabetes mellitus with exudative maculopathy |
| C10F.11 | Type II diabetes mellitus |
| C108.13 | Type I diabetes mellitus |
| C109711 | Type II diabetes mellitus - poor control |
| C100000 | Diabetes mellitus, juvenile type, no mention of complication |
| C109G00 | Non-insulin dependent diabetes mellitus with arthropathy |
| C108B00 | Insulin dependent diabetes mellitus with mononeuropathy |
| C109C12 | Type 2 diabetes mellitus with nephropathy |
| C10FQ00 | Type 2 diabetes mellitus with exudative maculopathy |
| C10F700 | Type 2 diabetes mellitus - poor control |
| C10FL00 | Type 2 diabetes mellitus with persistent proteinuria |
| C10B000 | Steroid induced diabetes mellitus without complication |
| C108400 | Unstable insulin dependent diabetes mellitus |
| C109900 | Non-insulin-dependent diabetes mellitus without complication |
| C10EL00 | Type 1 diabetes mellitus with persistent microalbuminuria |
| C10EK00 | Type 1 diabetes mellitus with persistent proteinuria |
| C108900 | Insulin dependent diabetes maturity onset |
| C107.11 | Diabetes mellitus with gangrene |
| C107.12 | Diabetes with gangrene |
| C10FN00 | Type 2 diabetes mellitus with ketoacidosis |
| C105.00 | Diabetes mellitus with ophthalmic manifestation |
| C10y.00 | Diabetes mellitus with other specified manifestation |
| C107200 | Diabetes mellitus, adult with gangrene |
| C10A100 | Malnutrition-related diabetes mellitus with ketoacidosis |
| C10F200 | Type 2 diabetes mellitus with neurological complications |
| C105z00 | Diabetes mellitus not otherwise specified with ophthalmic manifestation |
| C10FK00 | Hyperosmolar non-ketotic state in type 2 diabetes mellitus |
| C109400 | Non-insulin dependent diabetes mellitus with ulcer |
| C104100 | Diabetes mellitus, adult onset, with renal manifestation |
| C104z00 | Diabetes mellitus with nephropathy not otherwise specified |
| C10E800 | Type 1 diabetes mellitus - poor control |
| C10FH00 | Type 2 diabetes mellitus with neuropathic arthropathy |
| C107.00 | Diabetes mellitus with peripheral circulatory disorder |
| C109K00 | Hyperosmolar non-ketotic state in type 2 diabetes mellitus |
| C10D.00 | Diabetes mellitus autosomal dominant type 2 |
| C109J11 | Insulin treated non-insulin dependent diabetes mellitus |
| C10FF00 | Type 2 diabetes mellitus with peripheral angiopathy |
| C10K.00 | Type A insulin resistance |
| C108711 | Type I diabetes mellitus with retinopathy |
| C101y00 | Other specified diabetes mellitus with ketoacidosis |
| C100.00 | Diabetes mellitus with no mention of complication |
| C10EE00 | Type 1 diabetes mellitus with hypoglycaemic coma |
| C106100 | Diabetes mellitus, adult onset, + neurological manifestation |
| C108J00 | Insulin dependent diabetes mellitus with neuropathic arthropathy |
| C102000 | Diabetes mellitus, juvenile type, with hyperosmolar coma |
| C109500 | Non-insulin dependent diabetes mellitus with gangrene |
| C10E900 | Type 1 diabetes mellitus maturity onset |
| C10EN00 | Type 1 diabetes mellitus with ketoacidotic coma |
| C109H00 | Non-insulin dependent d m with neuropathic arthropathy |
| C108712 | Type 1 diabetes mellitus with retinopathy |
| C105100 | Diabetes mellitus, adult onset, + ophthalmic manifestation |
| C108C00 | Insulin dependent diabetes mellitus with polyneuropathy |
| C101z00 | Diabetes mellitus not otherwise specified with ketoacidosis |
| C103000 | Diabetes mellitus, juvenile type, with ketoacidotic coma |
| C108E11 | Type I diabetes mellitus with hypoglycaemic coma |
| C109612 | Type 2 diabetes mellitus with retinopathy |
| C10E200 | Type 1 diabetes mellitus with neurological complications |
| C102100 | Diabetes mellitus, adult onset, with hyperosmolar coma |
| C10F311 | Type II diabetes mellitus with multiple complications |
| C10C.00 | Diabetes mellitus autosomal dominant |
| C109D00 | Non-insulin dependent diabetes mellitus with hypoglycaemic coma |
| C10M.00 | Lipoatrophic diabetes mellitus |
| C10E400 | Unstable type 1 diabetes mellitus |
| C108F00 | Insulin dependent diabetes mellitus with diabetic cataract |
| C108E00 | Insulin dependent diabetes mellitus with hypoglycaemic coma |
| C108500 | Insulin dependent diabetes mellitus with ulcer |
| C109E12 | Type 2 diabetes mellitus with diabetic cataract |
| C10FE00 | Type 2 diabetes mellitus with diabetic cataract |
| C10E312 | Insulin dependent diabetes mellitus with multiple complications |
| C109B00 | Non-insulin dependent diabetes mellitus with polyneuropathy |
| C10z.00 | Diabetes mellitus with unspecified complication |
| C109712 | Type 2 diabetes mellitus - poor control |
| C108812 | Type 1 diabetes mellitus - poor control |
| C109212 | Type 2 diabetes mellitus with neurological complications |
| C109512 | Type 2 diabetes mellitus with gangrene |
| C108y00 | Other specified diabetes mellitus with multiple comps |
| C10EC00 | Type 1 diabetes mellitus with polyneuropathy |
| C10C.11 | Maturity onset diabetes in youth |
| C108811 | Type I diabetes mellitus - poor control |
| C10FD00 | Type 2 diabetes mellitus with hypoglycaemic coma |
| C108000 | Insulin-dependent diabetes mellitus with renal complications |
| C10F711 | Type II diabetes mellitus - poor control |
| C10F100 | Type 2 diabetes mellitus with ophthalmic complications |
| C105y00 | Other specified diabetes mellitus with ophthalmic complications |
| C109B11 | Type II diabetes mellitus with polyneuropathy |
| C10E000 | Type 1 diabetes mellitus with renal complications |
| C10E100 | Type 1 diabetes mellitus with ophthalmic complications |
| C10E300 | Type 1 diabetes mellitus with multiple complications |
| C109H11 | Type II diabetes mellitus with neuropathic arthropathy |
| C10F900 | Type 2 diabetes mellitus without complication |
| C109E11 | Type II diabetes mellitus with diabetic cataract |
| C10F400 | Type 2 diabetes mellitus with ulcer |
| C108211 | Type I diabetes mellitus with neurological complications |
| C108100 | Insulin-dependent diabetes mellitus with ophthalmic comps |
| C10EF00 | Type 1 diabetes mellitus with diabetic cataract |
| C10F611 | Type II diabetes mellitus with retinopathy |
| C109G12 | Type 2 diabetes mellitus with arthropathy |
| C10E411 | Unstable type I diabetes mellitus |
| C109011 | Type II diabetes mellitus with renal complications |
| C109100 | Non-insulin-dependent diabetes mellitus with ophthalmic complications |
| C10FB11 | Type II diabetes mellitus with polyneuropathy |
| C109A11 | Type II diabetes mellitus with mononeuropathy |
| C100z00 | Diabetes mellitus not otherwise specified with no mention of complication |
| C10E.12 | Insulin dependent diabetes mellitus |
| C10G.00 | Secondary pancreatic diabetes mellitus |
| C10FP00 | Type 2 diabetes mellitus with ketoacidotic coma |
| C108511 | Type I diabetes mellitus with ulcer |
| C108300 | Insulin dependent diabetes mellitus with multiple complications |
| C10A.00 | Malnutrition-related diabetes mellitus |
| C108200 | Insulin-dependent diabetes mellitus with neurological comps |
| C109000 | Non-insulin-dependent diabetes mellitus with renal comps |
| C101000 | Diabetes mellitus, juvenile type, with ketoacidosis |
| C10F911 | Type II diabetes mellitus without complication |
| C10EJ00 | Type 1 diabetes mellitus with neuropathic arthropathy |
| C109F00 | Non-insulin-dependent d m with peripheral angiopathy |
| C10E412 | Unstable insulin dependent diabetes mellitus |
| C101100 | Diabetes mellitus, adult onset, with ketoacidosis |
| C109F11 | Type II diabetes mellitus with peripheral angiopathy |
| C109411 | Type II diabetes mellitus with ulcer |
| C10EQ00 | Type 1 diabetes mellitus with gastroparesis |
| C109200 | Non-insulin-dependent diabetes mellitus with neuro comps |
| C109D11 | Type II diabetes mellitus with hypoglycaemic coma |
| C108A00 | Insulin-dependent diabetes without complication |
| C107400 | NIDDM with peripheral circulatory disorder |
| C10K000 | Type A insulin resistance without complication |
| C10F011 | Type II diabetes mellitus with renal complications |
| C108D00 | Insulin dependent diabetes mellitus with nephropathy |
| C109611 | Type II diabetes mellitus with retinopathy |
| C10FG00 | Type 2 diabetes mellitus with arthropathy |
| C103y00 | Other specified diabetes mellitus with coma |
| C109C00 | Non-insulin dependent diabetes mellitus with nephropathy |
| C109111 | Type II diabetes mellitus with ophthalmic complications |
| C106.11 | Diabetic amyotrophy |
| C10D.11 | Maturity onset diabetes in youth type 2 |
| C108411 | Unstable type I diabetes mellitus |
| C108J11 | Type I diabetes mellitus with neuropathic arthropathy |
| C108600 | Insulin dependent diabetes mellitus with gangrene |
| C109F12 | Type 2 diabetes mellitus with peripheral angiopathy |
| C10FL11 | Type II diabetes mellitus with persistent proteinuria |
| C109D12 | Type 2 diabetes mellitus with hypoglycaemic coma |
| C10H.00 | Diabetes mellitus induced by non-steroid drugs |
| C108011 | Type I diabetes mellitus with renal complications |
| C106y00 | Other specified diabetes mellitus with neurological comps |
| C108212 | Type 1 diabetes mellitus with neurological complications |
| C109511 | Type II diabetes mellitus with gangrene |
| C109300 | Non-insulin-dependent diabetes mellitus with multiple comps |
| C10EM11 | Type I diabetes mellitus with ketoacidosis |
| C108H11 | Type I diabetes mellitus with arthropathy |
| C10EA11 | Type I diabetes mellitus without complication |
| C10FA00 | Type 2 diabetes mellitus with mononeuropathy |
| C108911 | Type I diabetes mellitus maturity onset |
| C107100 | Diabetes mellitus, adult, + peripheral circulatory disorder |
| C10y100 | Diabetes mellitus, adult, + other specified manifestation |
| 8CR2.00 | Diabetes clinical management plan |
| C10FR00 | Type 2 diabetes mellitus with gastroparesis |
| C10z100 | Diabetes mellitus, adult onset, + unspecified complication |
| C10zy00 | Other specified diabetes mellitus with unspecified comps |
| C10zz00 | Diabetes mellitus not otherwise specified with unspecified complication |
| C108G00 | Insulin dependent diabetes mellitus with peripheral angiopathy |
| C108z00 | Unspecified diabetes mellitus with multiple complications |
| C109C11 | Type II diabetes mellitus with nephropathy |
| C10FJ11 | Insulin treated Type II diabetes mellitus |
| C107z00 | Diabetes mellitus not otherwise specified with peripheral circulatory disorder |
| C103z00 | Diabetes mellitus not otherwise specified with ketoacidotic coma |
| C10F300 | Type 2 diabetes mellitus with multiple complications |
| C108H00 | Insulin dependent diabetes mellitus with arthropathy |
| C109412 | Type 2 diabetes mellitus with ulcer |
| C10EN11 | Type I diabetes mellitus with ketoacidotic coma |
| C10A000 | Malnutrition-related diabetes mellitus with coma |
| C108D11 | Type I diabetes mellitus with nephropathy |
| C109H12 | Type 2 diabetes mellitus with neuropathic arthropathy |
| C10H000 | DM induced by non-steroid drugs without complication |
| C106000 | Diabetes mellitus, juvenile, + neurological manifestation |
| C109211 | Type II diabetes mellitus with neurological complications |
| C10EB00 | Type 1 diabetes mellitus with mononeuropathy |
| C108512 | Type 1 diabetes mellitus with ulcer |
| C10z000 | Diabetes mellitus, juvenile type, + unspecified complication |
| C103100 | Diabetes mellitus, adult onset, with ketoacidotic coma |
| C107300 | IDDM with peripheral circulatory disorder |
| C109E00 | Non-insulin dependent diabetes mellitus with diabetic cataract |
| C10EA00 | Type 1 diabetes mellitus without complication |
| C105000 | Diabetes mellitus, juvenile type, + ophthalmic manifestation |
| C10E600 | Type 1 diabetes mellitus with gangrene |
| C109112 | Type 2 diabetes mellitus with ophthalmic complications |
| C107000 | Diabetes mellitus, juvenile +peripheral circulatory disorder |
| C108E12 | Type 1 diabetes mellitus with hypoglycaemic coma |
| C10yz00 | Diabetes mellitus not otherwise specified with other specified manifestation |
| C109A00 | Non-insulin dependent diabetes mellitus with mononeuropathy |
| C102z00 | Diabetes mellitus not otherwise specified with hyperosmolar coma |
| C10E812 | Insulin dependent diabetes mellitus - poor control |
| 66Ao.00 | Diabetes type 2 review |
| 66An.00 | Diabetes type 1 review |
| C10FM11 | Type II diabetes mellitus with persistent microalbuminuria |
| C10F411 | Type II diabetes mellitus with ulcer |
| C10E311 | Type I diabetes mellitus with multiple complications |
| C10EC11 | Type I diabetes mellitus with polyneuropathy |
| C10N100 | Cystic fibrosis related diabetes mellitus |
| C10EG00 | Type 1 diabetes mellitus with peripheral angiopathy |
| C10FE11 | Type II diabetes mellitus with diabetic cataract |
| C10E712 | Insulin dependent diabetes mellitus with retinopathy |
| C10E511 | Type I diabetes mellitus with ulcer |
| C104000 | Diabetes mellitus, juvenile type, with renal manifestation |
| C10N000 | Secondary diabetes mellitus without complication |
| C10E711 | Type I diabetes mellitus with retinopathy |
| C10FA11 | Type II diabetes mellitus with mononeuropathy |
| C10FS00 | Maternally inherited diabetes mellitus |
| C10ER00 | Latent autoimmune diabetes mellitus in adult |
| C108A11 | Type I diabetes mellitus without complication |
| C10E911 | Type I diabetes mellitus maturity onset |
| C10G000 | Secondary pancreatic diabetes mellitus without complication |
| C108912 | Type 1 diabetes mellitus maturity onset |
| C108412 | Unstable type 1 diabetes mellitus |
| C10E912 | Insulin dependent diabetes maturity onset |
| C10EP11 | Type I diabetes mellitus with exudative maculopathy |
| C10E112 | Insulin-dependent diabetes mellitus with ophthalmic comps |
| C10C.12 | Maturity onset diabetes in youth type 1 |
| C10F211 | Type II diabetes mellitus with neurological complications |
| C10E512 | Insulin dependent diabetes mellitus with ulcer |
| C10FD11 | Type II diabetes mellitus with hypoglycaemic coma |
| C108B11 | Type I diabetes mellitus with mononeuropathy |
| C10E111 | Type I diabetes mellitus with ophthalmic complications |
| C10EE12 | Insulin dependent diabetes mellitus with hypoglycaemic coma |
| C10EA12 | Insulin-dependent diabetes without complication |
| C10A500 | Malnutrition-related diabetes mellitus with peripheral circulatory complications |
| C10EF12 | Insulin dependent diabetes mellitus with diabetic cataract |
| C10F111 | Type II diabetes mellitus with ophthalmic complications |
| 1434 | H/O: diabetes mellitus |
| 66AJ. | Diabetic - poor control |
| 66AJ0 | Chronic hyperglycaemia |
| 66AJ1 | Brittle diabetes |
| 66AJz | Diabetic - poor control not otherwise specified |
| C1001 | Diab.mell.no comp. - adult |
| C100z | Diab.mell.no comp. - onset not otherwise specified |
| C107y | Other specified diabetes mellitus+ peripheral circulatory complications |
| C1085 | Insulin dependent diabetes mellitus +ulcer |
| C108B | Insulin dependent diabetes mellitus with mononeuropathy |
| C10A2 | Malnutrition-related diabetes mellitus + renal complications |
| C10A3 | Malnutrition-related diabetes mellitus + ophthalmic complications |
| C10A4 | Malnutrition-related diabetes mellitus + neurologic complications |
| C10A5 | Malnutrition-related diabetes mellitus + peripheral circulatory complications |
| C10A6 | Malnutrition-related diabetes mellitus + multiple complications |
| C10A7 | Malnutrition-related diabetes mellitus without complications |
| C10AW | Malnutrition-related diabetes mellitus + unspecified complications |
| C10AX | Malnutrition-related diabetes mellitus + other specified complications |
| C10L. | Fibrocalculous pancreatopathy |
| C10L0 | Fibrocalculous pancreatopathy without complications |
| C10M0 | Lipoatrophic diabetes mellitus without complications |
| F372. | Polyneuropathy in diabetes |
| F3720 | Acute painful diabetic neuropathy |
| F3721 | Chron painful diabetic neuropathy |
| F3722 | Asymptomatic diabetic neuropathy |
| **Heart failure** | |
| G58.. | Heart failure |
| G580. | Congestive heart failure |
| G5800 | Acute congestive heart failure |
| G5801 | Chroncongestive heart failure |
| G5802 | Decompensated cardiac failure |
| G5803 | Compensated cardiac failure |
| G5804 | Cong heart failure due to valve disease |
| G581. | Left ventricular failure |
| G5810 | Acute left ventricular failure |
| G582. | Acute heart failure |
| G583. | Heart failure norm eject frac |
| G584. | Right ventricular failure |
| G58z. | Heart failure not otherwise specified |
| **Myocardial infarction** | |
| G32.. | Old myocardial infarction |
| **Peripheral vascular disease** | |
| G73.. | Other peripheral vascular disease |
| G734. | Peripheral arterial disease |
| G73y. | Other specified peripheral vascular disease |
| G73z. | Peripheral vascular disease not otherwise specified |
| G73z0 | Intermittent claudication |
| G73zz | Peripheral vascular disease not otherwise specified |
| Gyu74 | Other specified peripheral vascular disease |
| **Renal disease** | |
| K05..00 | Chronic renal failure |

| K050.00 | End stage renal failure |
| --- | --- |
| K060.11 | Impaired renal function |
| K0D..00 | End-stage renal disease |
| K060.00 | Renal impairment |
| K03..12 | Nephropathy, unspecified |
| K032.00 | Membranoproliferative nephritis unspecified |
| K08z.00 | Impaired renal function disorder not otherwise specified |
| K05..12 | End stage renal failure |
| 1Z13. | Chronic kidney disease stage 4 |
| 1Z14. | Chronic kidney disease stage 5 |
| 1Z1H. | CKD stage 4 with proteinuria |
| 1Z1J. | CKD stage 4 without proteinuria |
| 1Z1K. | CKD stage 5 with proteinuria |
| 1Z1L. | CKD stage 5 without proteinuria |
| K054. | Chronic kidney disease stage 4 |
| K055. | Chronic kidney disease stage 5 |
| **Venous thromboembolism** | |
| 14AC. | H/O: pulmonary embolus |
| G401. | Pulmonary embolism |
| G4010 | Post-operative pulmonary embolism |
| G4011 | Recurrent pulmonary embolism |
| 14A81 | H/O: Deep Vein Thrombosis |
| G801C | DVT of leg related air travel |
| G801D | Deep vein thromb lower limb |
| G801E | DVT leg related intra drug use |
| G801F | Deep vein thromb peroneal vein |
| G801G | Recurrent deep vein thrombosis |
| G82z0 | Embolus of vein NOS |
| **Asthma** | |
| H33.. | Asthma |
| H330. | Extrinsic (atopic) asthma |
| H3300 | Extrinsic asthma - no status |
| H3301 | Extrinsic asthma + status |
| H330z | Extrinsic asthma NOS |
| H331. | Intrinsic asthma |
| H3310 | Intrinsic asthma - no status |
| H3311 | Intrinsic asthma + status |
| H331z | Intrinsic asthma NOS |
| H332. | Mixed asthma |
| H333. | Acute exacerbation of asthma |
| H334. | Brittle asthma |
| H335. | Chron asthm w fix airflw obstr |
| H33z. | Asthma unspecified |
| H33z0 | Status asthmaticus NOS |
| H33z1 | Asthma attack |
| H33z2 | Late-onset asthma |
| H33zz | Asthma NOS |
| **Chronic obstructive pulmonary disease** | |
| H3... | Chronic obstructive pulm.dis. |
| H300. | Tracheobronchitis NOS |
| H3121 | Emphysematous bronchitis |
| H3122 | Acute exacerbation of COAD |
| H32.. | Emphysema |
| H320. | Chronic bullous emphysema |
| H3200 | Segmental bullous emphysema |
| H3201 | Zonal bullous emphysema |
| H3202 | Giant bullous emphysema |
| H3203 | Bullous emphysema + collapse |
| H320z | Chronic bullous emphysema NOS |
| H321. | Panlobular emphysema |
| H322. | Centrilobular emphysema |
| H32y. | Other emphysema |
| H32y0 | Acute vesicular emphysema |
| H32y1 | Atrophic (senile) emphysema |
| H32y2 | MacLeod's unilateral emphysema |
| H32yz | Other emphysema NOS |
| H32z. | Emphysema NOS |
| H36.. | Mild chron obstr pulm disease |
| H37.. | Mod chron obstr pulm disease |
| H38.. | Sev chron obstr pulm disease |
| H39.. | Very severe COPD |
| H3A.. | End stag chron obst airway dis |
| H3B.. | Asthma-COPD overlap syndrome |
| H3y.. | Chronic obstr.airway dis.OS |
| H3y0. | Chr obs pulm dis+ac l resp inf |
| H3y1. | Chr obs pulm dis+ac exac,unspc |
| H3z.. | Chronic obstr.airway dis.NOS |
| **Dyslipidaemia** | |
| C320. | Pure hypercholesterolaemia |
| C3200 | Familial hypercholesterolaemia |
| C3201 | Hyperbetalipoproteinaemia |
| C3202 | Hyperlipidaemia, group A |
| C3203 | LDL hyperlipoproteinaemia |
| C3204 | Fredrickson type IIa lipidaem |
| C3205 | Fam defect apolipoprot B-100 |
| C3206 | Polygenic hypercholesterolemia |
| C320y | Pure hypercholesterolaemia OS |
| C320z | Pure hypercholesterolaemia NOS |
| C321. | Pure hyperglyceridaemia |
| C3210 | Hypertriglyceridaemia |
| C322. | Mixed hyperlipidaemia |
| C3220 | Familial comb hyperlipidaemia |
| C324. | Hyperlipidaemia NOS |
| C328. | Dyslipidaemia |
| C329. | Hypercholesterolaemia |
| **Transient ischaemic attack** | |
| G65..00 | Transient cerebral ischaemia |
| G65z.00 | Transient cerebral ischaemia NOS |
| G65z100 | Intermittent cerebral ischaemia |
| G65..12 | Transient ischaemic attack |
| G65zz00 | Transient cerebral ischaemia NOS |
| G65y.00 | Other transient cerebral ischaemia |
| **Alzheimer’s disease** | |
| F110.00 | Alzheimer's disease |
| F110000 | Alzheimer's disease with early onset |
| F110100 | Alzheimer's disease with late onset |
| Eu00z00 | Dementia in Alzheimer's disease, unspecified |
| Eu00.00 | Dementia in Alzheimer's disease |
| Eu00000 | Dementia in Alzheimer's disease with early onset |
| Eu00100 | Dementia in Alzheimer's disease with late onset |
| Eu00200 | Dementia in Alzheimer's dis, atypical or mixed type |
| Eu00z00 | Dementia in Alzheimer's disease, unspecified |
| Fyu3000 | Other Alzheimer's disease |
| Eu00013 | Alzheimer's disease type 2 |
| Eu00111 | Alzheimer's disease type 1 |
| Eu00112 | Senile dementia,Alzheimer's type |
| Eu00113 | Primary degen dementia of Alzheimer's type, senile onset |
| Eu00z11 | Alzheimer's dementia unspec |
| **Other dementia or unspecified** | |
| Eu02y00 | Dementia in other specified diseases classif elsewhere |
| Eu02.00 | Dementia in other diseases classified elsewhere |
| Eu02z00 | Unspecified dementia |
| Eu04100 | Delirium superimposed on dementia |
| Eu02100 | Dementia in Creutzfeldt-Jakob disease |
| Eu02400 | Dementia in human immunodef virus [HIV] disease |
| Eu02200 | Dementia in Huntington's disease |
| Eu02300 | Dementia in Parkinson's disease |
| Eu02000 | Dementia in Pick's disease |
| Eu01111 | Predominantly cortical dementia |
| 1461 | H/O: dementia |
| 66h..00 | Dementia monitoring |
| 6AB..00 | Dementia annual review |
| 8CMZ.00 | Dementia care plan |
| 9hD0.00 | Excepted from dementia quality indicators: Patient unsuitabl |
| 9hD1.00 | Excepted from dementia quality indicators: Informed dissent |
| 9hD..00 | Exception reporting: dementia quality indicators |
| 9Ou1.00 | Dementia monitoring first letter |
| 9Ou2.00 | Dementia monitoring second letter |
| 9Ou3.00 | Dementia monitoring third letter |
| 9Ou4.00 | Dementia monitoring verbal invite |
| 9Ou5.00 | Dementia monitoring telephone invite |
| 9Ou..00 | Dementia monitoring administration |
| E000.00 | Uncomplicated senile dementia |
| E002000 | Senile dementia with paranoia |
| E002100 | Senile dementia with depression |
| E002.00 | Senile dementia with depressive or paranoid features |
| E002z00 | Senile dementia with depressive or paranoid features NOS |
| E003.00 | Senile dementia with delirium |
| E00..11 | Senile dementia |
| E00..12 | Senile/presenile dementia |
| E041.00 | Dementia in conditions EC |
| ZS7C500 | Language disorder of dementia |
| Eu02z16 | Senile dementia, depressed or paranoid type |
| Eu02z13 | Primary degenerative dementia NOS |
| Eu02z14 | Senile dementia NOS |
| Eu025 | Lewy body dementia |
| **Vascular dementia** | |
| Eu01.00 | Vascular dementia |
| Eu01000 | Vascular dementia of acute onset |
| Eu01100 | Multi-infarct dementia |
| Eu01200 | Subcortical vascular dementia |
| Eu01300 | Mixed cortical and subcortical vascular dementia |
| Eu01y00 | Other vascular dementia |
| Eu01z00 | Vascular dementia, unspecified |
| E004000 | Uncomplicated arteriosclerotic dementia |
| E004100 | Arteriosclerotic dementia with delirium |
| E004200 | Arteriosclerotic dementia with paranoia |
| E004300 | Arteriosclerotic dementia with depression |
| E004.00 | Arteriosclerotic dementia |
| E004.11 | Multi infarct dementia |
| E004z00 | Arteriosclerotic dementia NOS |
| Eu01.11 | [X]Arteriosclerotic dementia |
| **Younger onset dementia** | |
| E0010 | Presenile dementia – uncomplic |
| E001100 | Presenile dementia with delirium |
| E001200 | Presenile dementia with paranoia |
| E001300 | Presenile dementia with depression |
| E001.00 | Presenile dementia |
| E001z00 | Presenile dementia NOS |
| Eu00011 | Presenile dementia,Alzheimer's type |
| Eu00012 | Primary degen dementia, Alzheimer's type, presenile onset |
| **Liver disease** | |
| J615z13 | Cirrhosis of liver NOS |
| J61..00 | Cirrhosis and chronic liver disease |
| J62y.12 | Liver failure NOS |
| J62..00 | Liver abscess and sequelae of chronic liver disease |
| J612.00 | Alcoholic cirrhosis of liver |
| J613.00 | Alcoholic liver damage unspecified |
| J617.00 | Alcoholic hepatitis |
| J613000 | Alcoholic hepatic failure |
| J612.11 | Florid cirrhosis |
| J615.00 | Cirrhosis - non alcoholic |
| J615.11 | Portal cirrhosis |
| J615100 | Multilobular portal cirrhosis |
| J615300 | Diffuse nodular cirrhosis |
| J615400 | Fatty portal cirrhosis |
| J615500 | Hypertrophic portal cirrhosis |
| J615600 | Capsular portal cirrhosis |
| J615700 | Cardiac portal cirrhosis |
| J615D00 | Bacterial portal cirrhosis |
| J615H00 | Infectious cirrhosis NOS |
| J615y00 | Portal cirrhosis unspecified |
| J615z00 | Non-alcoholic cirrhosis NOS |
| J615z11 | Macronodular cirrhosis of liver |
| J615z12 | Cryptogenic cirrhosis of liver |
| J615z15 | Hepatic fibrosis |
| J616.00 | Biliary cirrhosis |
| J616000 | Primary biliary cirrhosis |
| J616100 | Secondary biliary cirrhosis |
| J616z00 | Biliary cirrhosis NOS |
| J61y400 | Hepatic fibrosis |
| J61y500 | Hepatic sclerosis |
| J635600 | Toxic liver disease with fibrosis and cirrhosis of liver |
| Jyu7100 | [X]Other and unspecified cirrhosis of liver |
| J616000 | Primary biliary cirrhosis |
| J616.00 | Biliary cirrhosis |
| J661700 | Primary sclerosing cholangitis |
| J616z00 | Biliary cirrhosis NOS |
| J614.00 | Chronic hepatitis |
| J614000 | Chronic persistent hepatitis |
| J614100 | Chronic active hepatitis |
| J614200 | Chronic aggressive hepatitis |
| J614400 | Chronic lobular hepatitis |
| J614y00 | Chronic hepatitis unspecified |
| J614z00 | Chronic hepatitis NOS |
| J615.00 | Cirrhosis - non alcoholic |
| J615D00 | Bacterial portal cirrhosis |
| J615H00 | Infectious cirrhosis NOS |
| J617000 | Chronic alcoholic hepatitis |
| J61y300 | Portal fibrosis without cirrhosis |
| G852200 | Oesophageal varices in cirrhosis of the liver |
| G852300 | Oesophageal varices in alcoholic cirrhosis of the liver |
| J622.00 | Hepatic coma |
| J622.11 | Encephalopathy - hepatic |
| J623.00 | Portal hypertension |
| J624.00 | Hepatorenal syndrome |
| J625.00 | [X] Hepatic failure |
| J625.11 | [X] Liver failure |
| J62y.11 | Hepatic failure NOS |
| J62y.13 | Hepatic failure |
| J63B.00 | Autoimmune hepatitis |
| J614111 | Autoimmune chronic active hepatitis |
| C3104 | Glycogenosis+hepatic cirrhosis |
| J611. | Acute alcoholic hepatitis |
| J6120 | Alcoholic fibrosis and scleros |
| J6143 | Recurrent hepatitis |
| J6150 | Unilobular portal cirrhosis |
| J6152 | Mixed portal cirrhosis |
| J6158 | Juvenile portal cirrhosis |
| J6159 | Pigmentary portal cirrhosis |
| J615A | Pipe-stem portal cirrhosis |
| J615B | Toxic portal cirrhosis |
| J615C | Xanthomatous portal cirrhosis |
| J615E | Cardituberculous cirrhosis |
| J615F | Syphilitic portal cirrhosis |
| J615G | Zooparasitic portal cirrhosis |
| J61y. | Other nonalc.chronic liver dis |
| J61y0 | Chronic yellow liver atrophy |
| J61y6 | Hepatic fibrosis + hepat scler |
| J61y8 | Nonalcoholic steatohepatitis |
| J61yz | Other nonalc.chronic liver NOS |
| J61z. | Chronic liver disease NOS |
| **Heavy drinker** | |
| 1365 | Heavy drinker - 7-9u/day |
| 1366 | Very heavy drinker - >9u/day |
| 136P. | Heavy drinker |
| 136R. | Binge drinker |
| 136W. | Alcohol misuse |
| E23.. | Alcohol dependence syndrome |
| 136Q. | Very heavy drinker |
| 136K. | Alcohol intake above rec limit |
| 388u. | Fast alcohol screening test |
| E250. | Alcohol abuse - nondependent |
| 136S. | Hazardous alcohol use |
| 136T. | Harmful alcohol use |
| E2500 | Alcohol abuse - unspecified |
| 8IAF. | Brief intervention for excessive alcohol consumption declined |
| 8IAt. | Extended intervention for excessive alcohol consumption declined |
| 9NzA. | Hospital attendance related to personal alcohol consumption |
| E250. | Nondependent alcohol abuse |
| E2500 | Nondependent alcohol abuse, unspecified |
| E2501 | Nondependent alcohol abuse, continuous |
| E2502 | Nondependent alcohol abuse, episodic |
| E250z | Nondependent alcohol abuse NOS |
| Eu101 | [X]Mental and behavioural disorders due to use of alcohol: harmful use |
| **Social drinker** | |
| 136O. | Moderate drinker |
| 1363 | Light drinker - 1-2u/day |
| 136F. | Spirit drinker |
| 136H. | Drinks beer and spirits |
| 136L. | Alcohol intake within rec limt |
| 136N. | Light drinker |
| 136J. | Social drinker |
| 1362 | Trivial drinker - <1u/day |
| 1364 | Moderate drinker - 3-6u/day |
| 136I. | Drinks wine |
| 136G. | Beer drinker |
| **Ex-heavy drinker** | |
| 136D. | Ex-heavy drinker - (7-9u/day) |
| 136E. | Ex-very heavy drinker-(>9u/d) |
| **Ex-social drinker** | |
| 1367 | Stopped drinking alcohol M 136C. Ex-moderate drinker - (3-6u/d) |
| 136A. | Ex-trivial drinker (<1u/day) |
| 136B. | Ex-light drinker - (1-2u/day) |
| **Never drinks** | |
| 1361 | Teetotaller |
| **Stopped drinking alcohol** | |
| 136M. | Current non drinker |
| E2503 | Nondependent alcohol abuse in remission |
| E2313 | Chronic alcoholism in remission |
| **Alcoholism** | |
| E230. | Acute alcoholic intoxication in alcoholism |
| E2300 | Acute alcoholic intoxication, unspecified, in alcoholism |
| E2301 | Continuous acute alcoholic intoxication in alcoholism |
| E2302 | Episodic acute alcoholic intoxication in alcoholism |
| E2303 | Acute alcoholic intoxication in remission, in alcoholism |
| E230z | Acute alcoholic intoxication in alcoholism NOS |
| ZV113 | [V]Personal history of alcoholism |
| 1462. | H/O: alcoholism |
| E23.. | Alcohol dependence syndrome |
| E231. | Chronic alcoholism |
| E2310 | Unspecified chronic alcoholism |
| E2311 | Continuous chronic alcoholism |
| E2312 | Episodic chronic alcoholism |
| E231z | Chronic alcoholism NOS |
| E23z. | Alcohol dependence syndrome NOS |
| Eu102 | [X]Mental and behavioural disorders due to use of alcohol: dependence syndrome |
| E012. | Other alcoholic dementia |
| E0120 | Chronic alcoholic brain syndrome |
| F11x0 | Cerebral degeneration due to alcoholism |
| F1440 | Cerebellar ataxia due to alcoholism |
| E0111 | Korsakov's alcoholic psychosis with peripheral neuritis |
| F375. | Alcoholic polyneuropathy |
| F3941 | Alcoholic myopathy |
| G555. | Alcoholic cardiomyopathy |
| J153. | Alcoholic gastritis |
| J610. | Alcoholic fatty liver |
| J617. | Alcoholic hepatitis |
| J6170 | Chronic alcoholic hepatitis |
| J611. | Acute alcoholic hepatitis |
| J6120 | Alcoholic fibrosis and sclerosis of liver |
| G8523 | Oesophageal varices in alcoholic cirrhosis of the liver |
| J612. | Alcoholic cirrhosis of liver |
| J6130 | Alcoholic hepatic failure |
| J613. | Alcoholic liver damage unspecified |
| 66e.. | Alcohol disorder monitoring |
| 66e0. | Alcohol abuse monitoring |
| 7P221 | Delivery of rehabilitation for alcohol addiction |
| 9k1A. | Brief intervention for excessive alcohol consumption completed |
| 9k1B. | Extended intervention for excessive alcohol consumption completed |
| 8G32. | Aversion therapy - alcoholism |
| **Prescription of beta blockers** | |
| bd… | BETA-ADRENOCEPTOR BLOCKERS |
| bd1.. | PROPRANOLOL HYDROCHLORIDE |
| bd11. | PROPRANOLOL 10mg tablets |
| bd12. | PROPRANOLOL 40mg tablets |
| bd13. | PROPRANOLOL 80mg tablets |
| bd14. | PROPRANOLOL 160mg tablets |
| bd15. | *ANGILOL 10mg tablets |
| bd16. | *ANGILOL 40mg tablets |
| bd17. | *ANGILOL 80mg tablets |
| bd18. | *ANGILOL 160mg tablets |
| bd19. | *APSOLOL 10mg tablets |
| bd1A. | *PROPANIX 10mg tablets |
| bd1B. | *PROPANIX 40mg tablets |
| bd1C. | *PROPANIX 80mg tablets |
| bd1D. | *PROPANIX 160mg tablets |
| bd1E. | *PROPANIX SR 160mg m/r caps |
| bd1F. | *BETADUR CR 160mg m/r tablets |
| bd1G. | BETA-PROGRANE 160mg m/r caps |
| bd1I. | PROPRANOLOL 5mg/5mL syrup |
| bd1J. | PROPRANOLOL 50mg/5mL syrup |
| bd1K. | *HALF-BETADUR CR 80mg m/r caps |
| bd1L. | HALF BETA-PROGRANE 80mg caps |
| bd1M. | *SLOPROLOL 80mg m/r capsules |
| bd1N. | *PROBETA LA 160mg m/r capsules |
| bd1O. | *LOPRANOL LA 160mg m/r caps |
| bd1P. | PROPRANOLOL HCL 10mg/5mL syrup |
| bd1Q. | PROPRANOLOL HCL 40mg/5mL syrup |
| bd1R. | PROPRANOLOL HCL 80mg/5mL syrup |
| bd1S. | *HALF PROPANIX LA 80mg m/r cap |
| bd1T. | *PROPANIX LA 160mg m/r caps |
| bd1U. | *HALF PROPATARD LA 80 m/r caps |
| bd1V. | *PROPATARD LA 160mg m/r caps |
| bd1W. | PROPRANOL HCL 5mg/5mL s/f soln |
| bd1X. | PROPRAN HCL 10mg/5mL s/f soln |
| bd1Y. | PROPRAN HCL 50mg/5mL s/f soln |
| bd1Z. | SYPROL 5mg/5mL oral solution |
| bd1a. | *APSOLOL 40mg tablets |
| bd1b. | *APSOLOL 80mg tablets |
| bd1c. | *APSOLOL 160mg tablets |
| bd1d. | *BEDRANOL 10mg tablets |
| bd1e. | *BEDRANOL 40mg tablets |
| bd1f. | *BEDRANOL 80mg tablets |
| bd1g. | BEDRANOL SR 160mg m/r capsules |
| bd1h. | *BERKOLOL 10mg tablets |
| bd1i. | *BERKOLOL 40mg tablets |
| bd1j. | *BERKOLOL 80mg tablets |
| bd1k. | *BERKOLOL 160mg tablets |
| bd1l. | HALF-INDERAL LA 80mg m/r caps |
| bd1l. | *HALF-INDERAL LA 80mg m/r caps |
| bd1m. | *INDERAL 10mg tablets |
| bd1n. | *INDERAL 40mg tablets |
| bd1o. | *INDERAL 80mg tablets |
| bd1p. | *INDERAL 160mg tablets |
| bd1r. | *INDERAL-LA 160mg m/r capsules |
| bd1r. | INDERAL-LA 160mg m/r capsules |
| bd1s. | *SLOPROLOL 160mg m/r capsules |
| bd1t. | *CARDINOL 10mg tablets |
| bd1u. | *CARDINOL 40mg tablets |
| bd1v. | *CARDINOL 80mg tablets |
| bd1w. | *CARDINOL 160mg tablets |
| bd1x. | PROPRANOLOL 160mg m/r capsules |
| bd1y. | PROPRANOLOL 80mg m/r capsules |
| bd1z. | SYPROL 10mg/5mL oral solution |
| bd2.. | ACEBUTOLOL |
| bd21. | SECTRAL 100mg capsules |
| bd22. | SECTRAL 200mg capsules |
| bd23. | SECTRAL 400mg tablets |
| bd2w. | ACEBUTOLOL 100mg capsules |
| bd2x. | ACEBUTOLOL 200mg capsules |
| bd2y. | ACEBUTOLOL 400mg tablets |
| bd3.. | ATENOLOL |
| bd31. | TENORMIN 100mg tablets |
| bd32. | TENORMIN 25mg/5mL syrup |
| bd34. | TENORMIN LS 50mg tablets |
| bd35. | ATENOLOL 50mg tablets |
| bd36. | ATENOLOL 100mg tablets |
| bd37. | *TENORMIN CCU PACK |
| bd3a. | *ANTIPRESSAN 50mg tablets |
| bd3b. | *ANTIPRESSAN 100mg tablets |
| bd3c. | *TENORMIN 25 tablets |
| bd3c. | TENORMIN 25 tablets |
| bd3d. | *VASATEN 50mg tablets |
| bd3e. | *VASATEN 100mg tablets |
| bd3f. | ATENIX 50mg tablets |
| bd3g. | ATENIX 100mg tablets |
| bd3h. | *TOTAMOL 50mg tablets |
| bd3i. | *TOTAMOL 100mg tablets |
| bd3j. | ATENOLOL 25mg tablets |
| bd3k. | *TOTAMOL 25mg tablets |
| bd3l. | *ANTIPRESSAN 25mg tablets |
| bd3x. | ATENOLOL 25mg/5mL syrup |
| bd3z. | ATENIX 25mg tablets |
| bd4.. | BETAXOLOL HCL [B-BLOCKER] |
| bd41. | *KERLONE 20mg tablets |
| bd4z. | *BETAXOLOL HCL 20mg tablets |
| bd5.. | LABETALOL HYDROCHLORIDE |
| bd51. | LABETALOL 100mg tablets |
| bd52. | LABETALOL 200mg tablets |
| bd53. | LABETALOL 400mg tablets |
| bd54. | *LABROCOL 100mg tablets |
| bd55. | *LABROCOL 200mg tablets |
| bd56. | *LABROCOL 400mg tablets |
| bd57. | TRANDATE 50mg tablets |
| bd58. | TRANDATE 100mg tablets |
| bd59. | TRANDATE 200mg tablets |
| bd5a. | TRANDATE 400mg tablets |
| bd5t. | *LABETALOL 100mg tablets |
| bd5u. | *LABETALOL 200mg tablets |
| bd5v. | LABETALOL 50mg tablets |
| bd5w. | *LABETALOL 100mg tablets |
| bd5x. | *LABETALOL 200mg tablets |
| bd5y. | *LABETALOL 400mg tablets |
| bd6.. | METOPROLOL TARTRATE |
| bd61. | *BETALOC 50mg tablets |
| bd62. | *BETALOC 100mg tablets |
| bd64. | *BETALOC-SA DURULES 200mg |
| bd65. | LOPRESOR 50mg tablets |
| bd66. | LOPRESOR 100mg tablets |
| bd67. | *MEPRANIX 50mg tablets |
| bd68. | *MEPRANIX 100mg tablets |
| bd6b. | LOPRESOR SR 200mg m/r tablets |
| bd6c. | *ARBRALENE 50mg tablets |
| bd6d. | *ARBRALENE 100mg tablets |
| bd6e. | *TENSOMEX 100mg tablets |
| bd6w. | METOPROLOL 100mg tablets |
| bd6x. | METOPROLOL 50mg tablets |
| bd6z. | METOPROLOL 200mg m/r tablets |
| bd7.. | NADOLOL |
| bd71. | *CORGARD 40mg tablets |
| bd72. | CORGARD 80mg tablets |
| bd7y. | *NADOLOL 40mg tablets |
| bd7z. | NADOLOL 80mg tablets |
| bd8.. | OXPRENOLOL HYDROCHLORIDE |
| bd81. | OXPRENOLOL 20mg tablets |
| bd82. | OXPRENOLOL 40mg tablets |
| bd83. | OXPRENOLOL 80mg tablets |
| bd84. | OXPRENOLOL 160mg tablets |
| bd85. | *APSOLOX 20mg tablets |
| bd86. | *APSOLOX 40mg tablets |
| bd87. | *APSOLOX 80mg tablets |
| bd88. | *APSOLOX 160mg tablets |
| bd89. | *LARACOR 20mg tablets |
| bd8a. | *LARACOR 40mg tablets |
| bd8b. | *LARACOR 80mg tablets |
| bd8c. | *LARACOR 160mg tablets |
| bd8d. | *SLOW-PREN 160mg m/r tablets |
| bd8e. | SLOW-TRASICOR 160mg m/r tabs |
| bd8f. | *TRASICOR 20mg tablets |
| bd8g. | *TRASICOR 40mg tablets |
| bd8h. | *TRASICOR 80mg tablets |
| bd8i. | *TRASICOR 160mg tablets |
| bd8k. | *PARITANE 20mg tablets |
| bd8l. | *PARITANE 40mg tablets |
| bd8m. | *PARITANE 80mg tablets |
| bd8n. | *PARITANE 160mg tablets |
| bd8o. | *OXYPRENIX 160mg m/r tablets |
| bd8u. | OXPRENOLOL 160mg m/r tablets |
| bd9.. | *PENBUTOLOL SULPH[INGREDIENT] |
| bda.. | PINDOLOL |
| bda1. | VISKEN 5mg tablets |
| bda2. | VISKEN 15mg tablets |
| bda3. | *BETADREN 5mg tablets |
| bda4. | *BETADREN 15mg tablets |
| bday. | PINDOLOL 5mg tablets |
| bdaz. | PINDOLOL 15mg tablets |
| bdb.. | *PRACTOLOL [B-BLOCKER] |
| bdc.. | SOTALOL HYDROCHLORIDE |
| bdc1. | *BETA-CARDONE 40mg tablets |
| bdc1. | BETA-CARDONE 40mg tablets |
| bdc2. | BETA-CARDONE 80mg tablets |
| bdc3. | BETA-CARDONE 200mg tablets |
| bdc4. | SOTACOR 80mg tablets |
| bdc5. | *SOTACOR 160mg tablets |
| bdcu. | SOTALOL 40mg tablets |
| bdcv. | SOTALOL 80mg tablets |
| bdcw. | SOTALOL 200mg tablets |
| bdcx. | SOTALOL 160mg tablets |
| bdd.. | TIMOLOL MALEATE [B-BLOCKER] |
| bdd1. | *BETIM 10mg tablets |
| bdd1. | BETIM 10mg tablets |
| bdd2. | *BLOCADREN 10mg tablets |
| bddz. | TIMOLOL 10mg tablets |
| bde.. | COMPOUND BETA-BLOCKERS |
| bde1. | *CO-BETALOC tablets |
| bde2. | *CO-BETALOC SA m/r tablets |
| bde3. | *CORGARETIC-40 tablets |
| bde4. | *CORGARETIC-80 tablets |
| bde5. | *INDERETIC capsules |
| bde6. | *INDEREX m/r capsules |
| bde7. | KALTEN capsules |
| bde8. | *LASIPRESSIN tablets |
| bde9. | *LOPRESORETIC tablets |
| bdeQ. | *TENBEN capsules |
| bdeR. | COMBITENS m/r capsules |
| bdea. | *MODUCREN tablets |
| bdeb. | PRESTIM tablets |
| bdec. | *PRESTIM FORTE tablets |
| bded. | *SECADREX tablets |
| bdee. | *SOTAZIDE tablets |
| bdef. | *SPIROPROP tablets |
| bdeg. | TENORET-50 tablets |
| bdeh. | TENORETIC tablets |
| bdei. | *TOLERZIDE tablets |
| bdej. | *TRASIDREX tablets |
| bdek. | VISKALDIX tablets |
| bdel. | *CO-PRENOZIDE tablets |
| bdf.. | BISOPROLOL FUMARATE |
| bdf1. | BISOPROLOL FUMARATE 5mg tabs |
| bdf2. | BISOPROLOL FUMARATE 10mg tabs |
| bdf3. | *MONOCOR 5mg tablets |
| bdf4. | *MONOCOR 10mg tablets |
| bdf5. | EMCOR LS 5mg tablets |
| bdf5. | *EMCOR LS 5mg tablets |
| bdf6. | EMCOR 10mg tablets |
| bdf6. | *EMCOR 10mg tablets |
| bdf7. | *MONOZIDE-10 tablets |
| bdf8. | *BISOPROLOL+HYDROCHLOROTH tabs |
| bdf9. | CARDICOR 1.25mg tablets |
| bdfA. | CARDICOR 2.5mg tablets |
| bdfB. | CARDICOR 3.75mg tablets |
| bdfC. | CARDICOR 5mg tablets |
| bdfD. | CARDICOR 7.5mg tablets |
| bdfE. | CARDICOR 10mg tablets |
| bdfF. | *BIPRANIX 5mg tablets |
| bdfG. | *BIPRANIX 10mg tablets |
| bdfH. | *SOLOC 5mg tablets |
| bdfI. | *SOLOC 10mg tablets |
| bdfJ. | VIVACOR 10mg tablets |
| bdfK. | VIVACOR 5mg tablets |
| bdfL. | CONGESCOR 1.25mg tablets |
| bdfM. | CONGESCOR 2.5mg tablets |
| bdfw. | BISOPROLOL FUMARAT 1.25mg tabs |
| bdfx. | BISOPROLOL FUMARATE 2.5mg tabs |
| bdfy. | BISOPROLOL FUMARAT 3.75mg tabs |
| bdfz. | BISOPROLOL FUMARATE 7.5mg tabs |
| bdg.. | *XAMOTEROL FUMARATE |
| bdg1. | *XAMOTEROL 200mg tablets |
| bdg2. | *CORWIN 200mg tablets |
| bdh.. | *METOPROLOL FUMARATE |
| bdh1. | *METOPROLOL FUMAR 190mg tabs |
| bdh2. | *METOPROLOL FUMARATE 95mg tabs |
| bdh3. | *METOROS 190mg tablets |
| bdh4. | *METOROS 95mg tablets |
| bdi.. | *CARTEOLOL HYDROCHLORIDE |
| bdi1. | *CARTEOLOL 10mg tablets |
| bdi2. | *CARTROL 10mg tablets |
| bdj.. | CELIPROLOL HYDROCHLORIDE |
| bdj1. | CELIPROLOL 200mg tablets |
| bdj2. | CELECTOL 200mg tablets 28-CP |
| bdj3. | CELECTOL 200mg tablets |
| bdj4. | CELIPROLOL 400mg tablets |
| bdj5. | CELECTOL 400mg tablets |
| bdl.. | CARVEDILOL |
| bdl1. | EUCARDIC 12.5 tablets |
| bdl1. | *EUCARDIC 12.5 tablets |
| bdl2. | *EUCARDIC 25 tablets |
| bdl3. | CARVEDILOL 12.5mg tablets |
| bdl4. | CARVEDILOL 25mg tablets |
| bdl5. | CARVEDILOL 3.125mg tablets |
| bdl6. | CARVEDILOL 6.25mg tablets |
| bdl7. | *EUCARDIC 3.125 tablets |
| bdl8. | EUCARDIC 6.25 tablets |
| bdl8. | *EUCARDIC 6.25 tablets |
| bdm.. | NEBIVOLOL |
| bdm1. | NEBILET 5mg tablets |
| bdm2. | HYPOLOC 5mg tablets |
| bdmy. | NEBIVOLOL 2.5mg tablets |
| bdmz. | NEBIVOLOL 5mg tablets |
| bdn.. | PROPRANOLOL HYDROCHLORIDE [2] |
| bdn1. | SYPROL 50mg/5mL oral solution |
| bdn2. | BEDRANOL SR 80mg m/r capsules |
| bdn3. | *RAPRANOL SR 80mg m/r capsules |
| bdn4. | *RAPRANOL SR 160mg m/r caps |
| bdn5. | SYPROL 40mg/5mL oral solution |
| bdn6. | PROPRAN HCL 40mg/5mL s/f soln |
| bd38. | *BETA-ADALAT 50/20mg capsules |
| bd39. | *TENIF 50/20mg capsules |
| bdeA. | *METPROLOL+HYDROCHL 100/12.5mg |
| bdeB. | *METOPROL+HYDROCH 200/25mg m/r |
| bdeC. | *METOPROL+CHLORTHAL 100/12.5mg |
| bdeD. | *NADOLOL+BENDROFLU 40/5mg tabs |
| bdeE. | *NADOLOL+BENDROFLU 80/5mg tabs |
| bdeF. | *PENBUTOL+FRUSEM 40/20mg tabs |
| bdeG. | PINDOLOL+CLOPAMIDE 10/5mg tabs |
| bdeH. | *SOTAL+HYDROCHLOR 160/25mg tab |
| bdeJ. | *SOTAL+HYDROCHLO 80/12.5mg tab |
| bdeK. | *TIMOLOL+CO-AMILOZ 10/2.5/25mg |
| bdeL. | TIMOLOL+BENDROFL 10/2.5mg tabs |
| bdeM. | *TIMOLOL+BENDROFL 20/5mg tabs |
| bdeN. | TOTARETIC 50mg/12.5mg tablets |
| bdeO. | TOTARETIC 100mg/25mg tablets |
| bdeP. | *ATENOLOL+BENDRO 25/1.25mg cap |
| bdem. | CO-TENIDONE 50/12.5mg tablets |
| bden. | CO-TENIDONE 100/25mg tablets |
| bdeo. | ATENIXCO 50/12.5mg tablets |
| bdep. | *ATENIXCO 100/25mg tablets |
| bdeq. | *TENCHLOR 50/12.5mg tablets |
| bder. | *TENCHLOR 100/25mg tablets |
| bdes. | *BETA-ADALAT 50/20mg capsules |
| bdes. | BETA-ADALAT 50/20mg capsules |
| bdet. | TENIF 50/20mg capsules |
| bdeu. | *CO-PRENOZIDE 160/0.25mg tabs |
| bdev. | *PROPRANOL+BENDR 80/2.5mg caps |
| bdew. | *PROPRANOL+BENDR 160/5mg m/r |
| bdex. | *ACEBU+HYDROCH 200/12.5mg tabs |
| bdey. | ATENOL+CO-AMILOZ 50/2.5/25mg |
| bdez. | ATENOLOL+NIFEDIP 50/20mg m/r |
| **Prescription of angiotensin converting enzyme inhibitors** | |
| bi1.. | CAPTOPRIL |
| bi11. | ACEPRIL 12.5mg tablets |
| bi12. | ACEPRIL 25mg tablets |
| bi13. | ACEPRIL 25mg tablets x56 |
| bi14. | ACEPRIL 50mg tablets |
| bi15. | ACEPRIL 50mg tablets x56 |
| bi16. | *CAPOTEN 12.5mg tablets |
| bi17. | CAPOTEN 25mg tablets |
| bi18. | CAPOTEN 25mg tablets x56 |
| bi19. | CAPOTEN 50mg tablets |
| bi1A. | *HYPAPRIL 12.5mg tablets |
| bi1B. | *HYPAPRIL 25mg tablets |
| bi1C. | *HYPAPRIL 50mg tablets |
| bi1H. | NOYADA 5mg/5mL oral solution |
| bi1I. | CAPTOPRIL 5mg/5mL oral soln |
| bi1J. | NOYADA 25mg/5mL oral solution |
| bi1K. | CAPTOPRIL 25mg/5mL oral soln |
| bi1a. | CAPOTEN 50mg tablets x56 |
| bi1b. | ACEZIDE 50mg tablets x56 |
| bi1c. | *CAPOZIDE 50mg tablets x28 |
| bi1d. | *CAPOZIDE LS 25mg tabletsx28CP |
| bi1g. | ECOPACE 12.5mg tablets |
| bi1h. | ECOPACE 25mg tablets |
| bi1i. | ECOPACE 50mg tablets |
| bi1j. | *KAPLON 12.5mg tablets |
| bi1k. | *KAPLON 25mg tablets |
| bi1l. | *KAPLON 50mg tablets |
| bi1m. | *HYTENEZE 12.5 tablets |
| bi1n. | *HYTENEZE 25 tablets |
| bi1o. | *HYTENEZE 50 tablets |
| bi1p. | *TENSOPRIL 12.5mg tablets |
| bi1q. | *TENSOPRIL 25mg tablets |
| bi1r. | *TENSOPRIL 50mg tablets |
| bi1v. | CAPTOPRIL 12.5mg tablets |
| bi1w. | CAPTOPRIL 25mg tablets |
| bi1x. | CAPTOPRIL 25mg tablets x56 |
| bi1y. | CAPTOPRIL 50mg tablets x56 |
| bi1z. | CAPTOPRIL 50mg tablets |
| bi2.. | ENALAPRIL MALEATE |
| bi21. | INNOVACE 2.5mg tablets |
| bi22. | INNOVACE 5mg tablets |
| bi23. | INNOVACE 5mg tablets x28 |
| bi24. | INNOVACE 10mg tablets |
| bi25. | INNOVACE 10mg tablets x28 |
| bi26. | INNOVACE 20mg tablets |
| bi27. | INNOVACE 20mg tablets x28 |
| bi29. | *INNOVACE tabs titration pack |
| bi2A. | *ENALAPRIL MALEATE 2.5mg wafer |
| bi2B. | *ENALAPRIL MALEATE 5mg wafer |
| bi2C. | *ENALAPRIL MALEATE 10mg wafer |
| bi2D. | *ENALAPRIL MALEATE 20mg wafer |
| bi2E. | *INNOVACE MELT 2.5mg wafer |
| bi2F. | *INNOVACE MELT 5mg wafer |
| bi2G. | *INNOVACE MELT 10mg wafer |
| bi2H. | *INNOVACE MELT 20mg wafer |
| bi2J. | *PRALENAL 2.5mg tablets |
| bi2K. | *PRALENAL 5mg tablets |
| bi2L. | *PRALENAL 10mg tablets |
| bi2M. | *PRALENAL 20mg tablets |
| bi2a. | *ENALAPRIL MAL tabs titre pack |
| bi2t. | ENALAPRIL MALEATE 2.5mg tabs |
| bi2u. | ENALAPRIL MALEATE 5mg tablets |
| bi2v. | ENALAPRIL MALEATE 5mg tabs x28 |
| bi2w. | ENALAPRIL MALEATE 10mg tablets |
| bi2x. | ENALAPRIL MAL 10mg tabs x28 |
| bi2y. | ENALAPRIL MALEATE 20mg tablets |
| bi2z. | ENALAPRIL MAL 20mg tabs x28 |
| bi3.. | LISINOPRIL |
| bi31. | LISINOPRIL 2.5mg tablets |
| bi32. | LISINOPRIL 5mg tablets |
| bi33. | LISINOPRIL 10mg tablets |
| bi34. | LISINOPRIL 20mg tablets |
| bi35. | *CARACE 2.5mg tablets |
| bi36. | *CARACE 5mg tablets 28CP |
| bi37. | *CARACE 5mg tablets |
| bi38. | *CARACE 10mg tablets 28CP |
| bi39. | *CARACE 10mg tablets |
| bi3a. | *CARACE 20mg tablets 28CP |
| bi3b. | *CARACE 20mg tablets |
| bi3c. | ZESTRIL 2.5mg tablets 28CP |
| bi3c. | *ZESTRIL 2.5mg tablets 28CP |
| bi3d. | ZESTRIL 2.5mg tablets |
| bi3d. | *ZESTRIL 2.5mg tablets |
| bi3e. | ZESTRIL 5mg tablets 28CP |
| bi3f. | ZESTRIL 5mg tablets |
| bi3g. | ZESTRIL 10mg tablets 28CP |
| bi3h. | ZESTRIL 10mg tablets |
| bi3i. | ZESTRIL 20mg tablets 28CP |
| bi3j. | ZESTRIL 20mg tablets |
| bi3k. | CARACE 20 PLUS tablets |
| bi3l. | *CARACE 10 PLUS tablets |
| bi3m. | *ZESTORETIC tablets 28CP |
| bi3q. | *ZESTRIL 2.5mg starter pack |
| bi3r. | *LISINOPRIL 2.5mg tabs starter |
| bi3y. | LISINOPRIL 1mg/mL oral soln |
| bi4.. | QUINAPRIL |
| bi41. | QUINAPRIL 5mg tablets |
| bi42. | QUINAPRIL 10mg tablets |
| bi43. | QUINAPRIL 20mg tablets |
| bi44. | ACCUPRO 5mg tablets 28CP |
| bi45. | ACCUPRO 10mg tablets 28CP |
| bi46. | ACCUPRO 20mg tablets 28CP |
| bi47. | ACCURETIC tablets |
| bi49. | ACCUPRO 40mg tablets |
| bi4A. | QUINAPRIL 40mg tablets |
| bi4B. | QUINIL 5mg tablets |
| bi4C. | QUINIL 10mg tablets |
| bi4D. | QUINIL 20mg tablets |
| bi4E. | QUINIL 40mg tablets |
| bi5.. | PERINDOPRIL ERBUMINE |
| bi51. | PERINDOPRIL ERBUMINE 2mg tabs |
| bi52. | PERINDOPRIL ERBUMINE 4mg tabs |
| bi53. | *COVERSYL 2mg tablets |
| bi54. | *COVERSYL 4mg tablets |
| bi57. | PERINDOPRIL ERBUMINE 8mg tabs |
| bi58. | *COVERSYL 8mg tablets |
| bi6.. | RAMIPRIL |
| bi61. | RAMIPRIL 1.25mg capsules |
| bi62. | RAMIPRIL 2.5mg capsules |
| bi63. | RAMIPRIL 5mg capsules |
| bi64. | *TRITACE 1.25mg capsules |
| bi65. | *TRITACE 2.5mg capsules |
| bi66. | *TRITACE 5mg capsules |
| bi67. | RAMIPRIL 10mg capsules |
| bi68. | *TRITACE 10mg capsules |
| bi69. | *RAMIPRIL 2.5mg+5mg+10mg caps |
| bi6A. | *TRITACE Titration Pack caps |
| bi6B. | RAMIPRIL 1.25mg tablets |
| bi6C. | RAMIPRIL 2.5mg tablets |
| bi6D. | RAMIPRIL 5mg tablets |
| bi6E. | RAMIPRIL 10mg tablets |
| bi6F. | RAMIPRIL 2.5+5+10mg tabs pack |
| bi6G. | RAMIPRIL 2.5mg/5mL solution |
| bi6o. | TRITACE Titration Pack tablets |
| bi6p. | *RANACE 10mg capsules |
| bi6q. | *RANACE 5mg capsules |
| bi6r. | *RANACE 2.5mg capsules |
| bi6s. | *RANACE 1.25mg capsules |
| bi6t. | *LOPACE 2.5mg capsules |
| bi6u. | *LOPACE 5mg capsules |
| bi6v. | *LOPACE 10mg capsules |
| bi6w. | TRITACE 10mg tablets |
| bi6x. | TRITACE 5mg tablets |
| bi6y. | TRITACE 2.5mg tablets |
| bi6z. | TRITACE 1.25mg tablets |
| bi7.. | SODIUM FOSINOPRIL |
| bi71. | FOSINOPRIL 10mg tablets |
| bi72. | FOSINOPRIL 20mg tablets |
| bi73. | *STARIL 10mg tablets |
| bi74. | *STARIL 20mg tablets |
| bi8.. | CILAZAPRIL |
| bi81. | *CILAZAPRIL 250mcg tablets |
| bi82. | CILAZAPRIL 500mcg tablets |
| bi82. | *CILAZAPRIL 500mcg tablets |
| bi83. | *CILAZAPRIL 1mg tablets |
| bi83. | CILAZAPRIL 1mg tablets |
| bi84. | CILAZAPRIL 2.5mg tablets |
| bi84. | *CILAZAPRIL 2.5mg tablets |
| bi85. | *VASCACE 250micrograms tablets |
| bi86. | *VASCACE 500micrograms tablets |
| bi86. | VASCACE 500micrograms tablets |
| bi87. | *VASCACE 1mg tablets |
| bi87. | VASCACE 1mg tablets |
| bi88. | VASCACE 2.5mg tablets |
| bi88. | *VASCACE 2.5mg tablets |
| bi89. | VASCACE 5mg tablets |
| bi89. | *VASCACE 5mg tablets |
| bi8a. | CILAZAPRIL 5mg tablets |
| bi9.. | TRANDOLAPRIL |
| bi91. | TRANDOLAPRIL 500mcg capsules |
| bi92. | TRANDOLAPRIL 1mg capsules |
| bi93. | TRANDOLAPRIL 2mg capsules |
| bi94. | *GOPTEN 500micrograms capsules |
| bi94. | GOPTEN 500micrograms capsules |
| bi95. | GOPTEN 1mg capsules |
| bi95. | *GOPTEN 1mg capsules |
| bi96. | GOPTEN 2mg capsules |
| bi96. | *GOPTEN 2mg capsules |
| bi97. | *ODRIK 500micrograms capsules |
| bi98. | *ODRIK 1mg capsules |
| bi99. | *ODRIK 2mg capsules |
| bi9A. | *GOPTEN 4mg capsules |
| bi9A. | GOPTEN 4mg capsules |
| bi9z. | TRANDOLAPRIL 4mg capsules |
| biA.. | MOEXIPRIL |
| biA1. | MOEXIPRIL HCL 7.5mg tablets |
| biA2. | MOEXIPRIL HCL 15mg tablets |
| biA3. | PERDIX 7.5mg tablets |
| biA4. | PERDIX 15mg tablets |
| biB.. | IMIDAPRIL HYDROCHLORIDE |
| biB1. | TANATRIL 5mg tablets |
| biB2. | TANATRIL 10mg tablets |
| biB3. | TANATRIL 20mg tablets |
| biBx. | IMIDAPRIL HCL 20mg tablets |
| biBy. | IMIDAPRIL HCL 5mg tablets |
| biBz. | IMIDAPRIL HCL 10mg tablets |
| biC.. | PERINDOPRIL ARGININE |
| biC1. | COVERSYL ARGININE 2.5mg tabs |
| biC2. | PERINDOPRIL ARGININE 2.5mg tab |
| biC3. | COVERSYL ARGININE 5mg tablets |
| biC4. | PERINDOPRIL ARGININE 5mg tabs |
| biC5. | COVERSYL ARGININE 10mg tablets |
| biC6. | PERINDOPRIL ARGININE 10mg tabs |
| bA1.. | FELODIPINE+RAMIPRIL |
| bA11. | TRIAPIN MITE 2.5mg/2.5mg tabs |
| bA12. | TRIAPIN 5mg/5mg tablets |
| bA1y. | FELODIP+RAMIPRL 2.5/2.5mg tabs |
| bA1z. | FELODIPINE+RAMIPRIL 5/5mg tabs |
| bi1D. | *CAPTO-CO 25mg/12.5mg tablets |
| bi1E. | *CAPTO-CO 50mg/25mg tablets |
| bi1F. | *CO-ZIDOCAPT 25/12.5mg tablets |
| bi1G. | *CO-ZIDOCAPT 50mg/25mg tablets |
| bi1e. | CAPTOP+HYDROCHL 25/12.5mg tabs |
| bi1f. | CAPTOP+HYDROCHL 50/25mg tabs |
| bi1s. | CAPOZIDE 50mg/25mg tablets |
| bi28. | INNOZIDE 20/12.5mg tablets |
| bi2b. | ENALAP+HYDROCHL 20/12.5mg tabs |
| bi3n. | ZESTORETIC 20/12.5mg tablets |
| bi3p. | LISINO+HYDROCHL 20/12.5mg tabs |
| bi3s. | ZESTORETIC 10/12.5mg tablets |
| bi3t. | LISINO+HYDROCHL 10/12.5mg tabs |
| bi3u. | *CARALPHA 10/12.5mg tablets |
| bi3v. | *CARALPHA 20/12.5mg tablets |
| bi3w. | *LISICOSTAD HCT 20/12.5mg tabs |
| bi3x. | *LISICOSTAD HCT 10/12.5mg tabs |
| bi48. | QUINAPRIL+HYDROCHLOROTHIAZIDE |
| bi4F. | QUINAP+HYDROCHL 20/12.5mg tabs |
| bi55. | *PERIND ERB+INDAP 4/1.25mg tab |
| bi56. | *COVERSYL PLUS 4mg/1.25mg tabs |
| biC7. | COVRSYL ARGIN PLS 5/1.25mg tab |
| biC8. | PERIND ARG+INDAP 5/1.25mg tabs |
| **Prescription of angiotensin receptor blockers** | |
| bk3.. | LOSARTAN |
| bk31. | LOSARTAN POTASSIUM 25mg tabs |
| bk32. | LOSARTAN POTASSIUM 50mg tabs |
| bk33. | COZAAR HALF-STRENGTH 25mg tabs |
| bk34. | COZAAR 50mg tablets |
| bk37. | LOSARTAN POTASSIUM 100mg tabs |
| bk38. | COZAAR 100mg tablets |
| bk3B. | COZAAR 12.5mg tablets |
| bk3C. | LOSARTAN POTASSIUM 12.5mg tabs |
| bk3D. | COZAAR 2.5mg/mL oral susp |
| bk3E. | LOSARTAN POTASS 2.5mg/mL susp |
| bk3F. | ZOVENCAL 25mg tablets |
| bk3G. | ZOVENCAL 50mg tablets |
| bk3H. | ZOVENCAL 100mg tablets |
| bk4.. | VALSARTAN |
| bk41. | VALSARTAN 40mg capsules |
| bk42. | VALSARTAN 80mg capsules |
| bk43. | VALSARTAN 160mg capsules |
| bk44. | DIOVAN 40mg capsules |
| bk45. | DIOVAN 80mg capsules |
| bk46. | DIOVAN 160mg capsules |
| bk4A. | DIOVAN 40mg tablets |
| bk4B. | DIOVAN 320mg tablets |
| bk4C. | DIOVAN 3mg/mL oral solution |
| bk4s. | VALSARTAN 80mg tablets |
| bk4t. | VALSARTAN 160mg tablets |
| bk4u. | VALSARTAN 3mg/mL oral solution |
| bk4v. | VALSARTAN 320mg tablets |
| bk4w. | VALSARTAN 40mg tablets |
| bk5.. | IRBESARTAN |
| bk51. | IRBESARTAN 75mg tablets |
| bk52. | IRBESARTAN 150mg tablets |
| bk53. | IRBESARTAN 300mg tablets |
| bk54. | APROVEL 75mg tablets |
| bk55. | APROVEL 150mg tablets |
| bk56. | APROVEL 300mg tablets |
| bk7.. | CANDESARTAN CILEXETIL |
| bk71. | CANDESARTAN CILEXETIL 2mg tabs |
| bk72. | CANDESARTAN CILEXETIL 4mg tabs |
| bk73. | CANDESARTAN CILEXETIL 8mg tabs |
| bk74. | CANDESARTAN CILEXET 16mg tabs |
| bk75. | AMIAS 2mg tablets |
| bk76. | AMIAS 4mg tablets |
| bk77. | AMIAS 8mg tablets |
| bk78. | AMIAS 16mg tablets |
| bk79. | AMIAS 32mg tablets |
| bk7z. | CANDESARTAN CILEXETL 32mg tabs |
| bk8.. | TELMISARTAN |
| bk81. | TELMISARTAN 40mg tablets |
| bk82. | TELMISARTAN 80mg tablets |
| bk83. | MICARDIS 40mg tablets |
| bk84. | MICARDIS 80mg tablets |
| bk85. | MICARDIS 20mg tablets |
| bk8z. | TELMISARTAN 20mg tablets |
| bk9.. | EPROSARTAN |
| bk91. | TEVETEN 300mg tablets |
| bk92. | TEVETEN 400mg tablets |
| bk92. | *TEVETEN 400mg tablets |
| bk93. | TEVETEN 600mg tablets |
| bk9x. | EPROSARTAN 300mg tablets |
| bk9y. | EPROSARTAN 400mg tablets |
| bk9z. | EPROSARTAN 600mg tablets |
| bkB.. | OLMESARTAN |
| bkB1. | OLMESARTAN MEDOXOMIL 10mg tabs |
| bkB2. | OLMESARTAN MEDOXOMIL 20mg tabs |
| bkB3. | OLMESARTAN MEDOXOMIL 40mg tabs |
| bkB4. | OLMETEC 10mg tablets |
| bkB5. | OLMETEC 20mg tablets |
| bkB6. | OLMETEC 40mg tablets |
| bkJ.. | AZILSARTAN |
| bkJ1. | EDARBI 20mg tablets |
| bkJ2. | AZILSARTAN MEDOXOMIL 20mg tabs |
| bkJ3. | EDARBI 40mg tablets |
| bkJ4. | AZILSARTAN MEDOXOMIL 40mg tabs |
| bkJ5. | EDARBI 80mg tablets |
| bkJ6. | AZILSARTAN MEDOXOMIL 80mg tabs |
| bk35. | LOSART+HYDROCHLTHZ 50/12.5 tab |
| bk36. | COZAAR-COMP 50mg/12.5mg tabs |
| bk39. | COZAAR-COMP 100mg/25mg tablets |
| bk3A. | COZAAR-COMP 100mg/12.5mg tabs |
| bk3y. | LOSART+HYDRCHL 100/12.5mg tabs |
| bk3z. | LOSART+HYDROCHLTHZ 100/25 tabs |
| bk47. | CO-DIOVAN 160mg/12.5mg tablets |
| bk48. | CO-DIOVAN 160mg/25mg tablets |
| bk49. | CO-DIOVAN 80mg/12.5mg tablets |
| bk4x. | VALSART+HYDROCHL 80/12.5mg tab |
| bk4y. | VALSART+HYDROCHL 160/25mg tabs |
| bk4z. | VALSRT+HYDROCHL 160/12.5mg tab |
| bk57. | COAPROVEL 150mg/12.5mg tablets |
| bk58. | COAPROVEL 300mg/12.5mg tablets |
| bk59. | COAPROVEL 300mg/25mg tablets |
| bk5x. | IRBES+HYDROCHL 300mg/25mg tabs |
| bk5y. | IRBES+HYDROCHL 300/12.5mg tabs |
| bk5z. | IRBES+HYDROCHL 150/12.5mg tabs |
| bk86. | MICARDISPLUS 40mg/12.5mg tabs |
| bk87. | MICARDISPLUS 80mg/12.5mg tabs |
| bk88. | MICARDISPLUS 80mg/25mg tablets |
| bk8w. | TELMIS+HYDROCHL 80mg/25mg tabs |
| bk8x. | TELMIS+HYDROCHL 40/12.5mg tabs |
| bk8y. | TELMIS+HYDROCHL 80/12.5mg tabs |
| bkC.. | HYDROCHLOROTHIAZIDE+OLMESARTAN |
| bkC1. | OLMETEC PLUS 20mg/12.5mg tabs |
| bkC2. | OLMETEC PLUS 20mg/25mg tablets |
| bkC3. | OLMETEC PLUS 40mg/12.5mg tabs |
| bkCx. | OLMESAR+HYDROCH 40/12.5mg tabs |
| bkCy. | OLMESART+HYDROCHL 20/25mg tabs |
| bkCz. | OLMESAR+HYDROCH 20/12.5mg tabs |
| bkH.. | OLMESARTAN+AMLODIPINE |
| bkH1. | SEVIKAR 20mg/5mg tablets |
| bkH2. | SEVIKAR 40mg/5mg tablets |
| bkH3. | SEVIKAR 40mg/10mg tablets |
| bkHx. | OLMESART+AMLODIPN 40/10mg tabs |
| bkHy. | OLMESART+AMLODIPNE 40/5mg tabs |
| bkHz. | OLMESART+AMLODIPNE 20/5mg tabs |
| bkI.. | OLMESART+AMLODIP+HYDRCHLRTHIAZ |
| bkI1. | SEVIKAR HCT 20/5/12.5mg tabs |
| bkI2. | SEVIKAR HCT 40/5/12.5mg tabs |
| bkI3. | SEVIKAR HCT 40/10/12.5mg tabs |
| bkI4. | SEVIKAR HCT 40mg/5mg/25mg tabs |
| bkI5. | SEVIKAR HCT 40/10/25mg tabs |
| bkL.. | SACUBITRIL+VALSARTAN |
| bkL1. | ENTRESTO 24mg/26mg tablets |
| bkL2. | SACUBITRL+VALSRTN 24/26mg tabs |
| bkL3. | ENTRESTO 49mg/51mg tablets |
| bkL4. | SACUBITRL+VALSRTN 49/51mg tabs |
| bkL5. | ENTRESTO 97mg/103mg tablets |
| bkL6. | SACUBTRL+VALSRTN 97/103mg tabs |
| **Prescription of calcium channel blockers** | |
| bb3.. | VERAPAMIL HYDROCHLORIDE |
| bb31. | VERAPAMIL 40mg tablets |
| bb32. | VERAPAMIL 80mg tablets |
| bb33. | VERAPAMIL 120mg tablets |
| bb34. | *BERKATENS 40mg tablets |
| bb35. | *BERKATENS 80mg tablets |
| bb36. | *BERKATENS 120mg tablets |
| bb37. | *BERKATENS 160mg tablets |
| bb38. | *CORDILOX 40mg tablets |
| bb39. | *CORDILOX 80mg tablets |
| bb3A. | VERAPAMIL 240mg m/r tablets |
| bb3B. | HALF SECURON SR 120mg m/r tabs |
| bb3C. | VERAPAMIL 120mg m/r tablets |
| bb3D. | VERAPAMIL 40mg/5mL s/f soln |
| bb3F. | HALF-SECURON SR 120mg 28CP |
| bb3G. | *HYPANEZE 40 tablets |
| bb3H. | *HYPANEZE 80 tablets |
| bb3J. | *HYPANEZE 120 tablets |
| bb3K. | *VERAPRESS MR 240 m/r tablets |
| bb3L. | *ETHIMIL MR 240 m/r tablets |
| bb3M. | CORDILOX MR 240 m/r tablets |
| bb3N. | ZOLVERA 40mg/5mL oral solution |
| bb3O. | *RANVERA MR 240mg m/r tablets |
| bb3P. | VERA-TIL SR 240mg m/r tablets |
| bb3Q. | VERA-TIL SR 120mg m/r tablets |
| bb3a. | CORDILOX 120mg tablets |
| bb3b. | *CORDILOX 160mg tablets |
| bb3d. | *SECURON 40mg tablets |
| bb3e. | *SECURON 80mg tablets |
| bb3f. | *SECURON 120mg tablets |
| bb3g. | *SECURON 120mg tablets 56CP |
| bb3h. | *SECURON 160mg tablets 56CP |
| bb3i. | *SECURON 160mg tablets |
| bb3j. | SECURON SR 240mg m/r tablets |
| bb3k. | SECURON SR 240mg m/r tabs 28CP |
| bb3l. | UNIVER 120mg m/r capsules x28 |
| bb3m. | UNIVER 180mg m/r capsules x56 |
| bb3n. | UNIVER 240mg m/r capsules x28 |
| bb3p. | *GEANGIN 40mg tablets |
| bb3q. | *GEANGIN 80mg tablets |
| bb3r. | *GEANGIN 120mg tablets |
| bb3s. | VERTAB SR 240 m/r tablets |
| bb3v. | VERAPAMIL 120mg m/r capsules |
| bb3w. | VERAPAMIL 160mg tablets |
| bb3x. | *VERPAMIL HCL 120mg tabs x56 |
| bb3y. | VERAPAMIL 240mg m/r capsules |
| bb3z. | VERAPAMIL 180mg m/r capsules |
| bl5.. | DILTIAZEM HYDROCHLORIDE |
| bl51. | TILDIEM 60mg tablets |
| bl52. | *CALCICARD 60mg tablets |
| bl53. | *BRITIAZIM 60mg tablets |
| bl54. | ADIZEM-SR 120mg m/r tablets |
| bl55. | DILTIAZEM HCL 120mg m/r tabs |
| bl56. | *ANGIOZEM 60mg tablets |
| bl57. | *ADIZEM 60mg tablets |
| bl58. | TILDIEM RETARD 90mg m/r tabs |
| bl59. | TILDIEM RETARD 120mg m/r tabs |
| bl5A. | TILDIEM LA 300mg m/r capsules |
| bl5B. | ADIZEM-SR 90mg m/r capsules |
| bl5C. | ADIZEM-SR 120mg m/r capsules |
| bl5D. | ADIZEM-SR 180mg m/r capsules |
| bl5E. | ADIZEM-XL 300mg m/r capsules |
| bl5F. | DILZEM SR 60mg m/r capsules |
| bl5G. | DILZEM SR 90mg m/r capsules |
| bl5H. | DILZEM SR 120mg m/r capsules |
| bl5I. | ADIZEM-XL 240mg m/r capsules |
| bl5J. | ADIZEM-XL 180mg m/r capsules |
| bl5K. | ADIZEM-XL 120mg m/r capsules |
| bl5L. | DILZEM-XL 120mg m/r capsules |
| bl5M. | DILZEM-XL 180mg m/r capsules |
| bl5N. | DILZEM-XL 240mg m/r capsules |
| bl5O. | SLOZEM 120mg m/r capsules |
| bl5P. | SLOZEM 180mg m/r capsules |
| bl5Q. | SLOZEM 240mg m/r capsules |
| bl5R. | ANGITIL SR 90 m/r capsules |
| bl5S. | ANGITIL SR 120 m/r capsules |
| bl5T. | *METAZEM 60mg tablets |
| bl5U. | ANGITIL SR 180 m/r capsules |
| bl5V. | *CALCICARD CR 90mg m/r tablets |
| bl5V. | CALCICARD CR 90mg m/r tablets |
| bl5W. | CALCICARD CR 120mg m/r tablets |
| bl5W. | *CALCICARD CR 120mg m/r tabs |
| bl5X. | KENTIAZEM 60mg m/r capsules |
| bl5Y. | *OPTIL 60mg m/r tablets |
| bl5Z. | TILDIEM LA 200mg m/r capsules |
| bl5a. | DILTIAZEM HCL 90mg m/r tablets |
| bl5b. | DILTIAZEM HCL 300mg m/r caps |
| bl5c. | DILTIAZEM HCL 90mg m/r caps |
| bl5d. | DILTIAZEM HCL 120mg m/r caps |
| bl5e. | DILTIAZEM HCL 180mg m/r caps |
| bl5f. | DILTIAZEM HCL 60mg m/r caps |
| bl5g. | DILTIAZEM HCL 240mg m/r caps |
| bl5h. | DILTIAZEM HCL 200mg m/r caps |
| bl5j. | *ADIZEM-XL PLUS m/r capsules |
| bl5k. | *ANGIOZEM CR 90mg m/r tablets |
| bl5l. | DILCARDIA SR 60mg m/r capsules |
| bl5m. | *ANGIOZEM CR 120mg m/r tablets |
| bl5n. | ZEMTARD 300 XL m/r capsules |
| bl5o. | VIAZEM XL 120mg m/r capsules |
| bl5p. | VIAZEM XL 180mg m/r capsules |
| bl5q. | VIAZEM XL 240mg m/r capsules |
| bl5r. | VIAZEM XL 300mg m/r capsules |
| bl5s. | DILTIAZEM HCL 360mg m/r caps |
| bl5t. | VIAZEM XL 360mg m/r capsules |
| bl5u. | *CALAZEM 60mg m/r tablets |
| bl5v. | DILCARDIA SR 90mg m/r capsules |
| bl5w. | DILCARDIA SR 120mg m/r caps |
| bl5x. | ANGITIL XL 240 m/r capsules |
| bl5y. | ANGITIL XL 300 m/r capsules |
| bl5z. | DILTIAZEM HCL 60mg m/r tablets |
| bl7.. | NICARDIPINE HYDROCHLORIDE |
| bl71. | CARDENE 20mg capsules |
| bl72. | CARDENE 30mg capsules |
| bl73. | CARDENE SR 30mg m/r capsules |
| bl74. | CARDENE SR 45mg m/r capsules |
| bl7w. | NICARDIPINE 45mg m/r capsules |
| bl7x. | NICARDIPINE 30mg m/r capsules |
| bl7y. | NICARDIPINE 20mg capsules |
| bl7z. | NICARDIPINE 30mg capsules |
| bl8.. | NIFEDIPINE |
| bl81. | ADALAT 5mg capsules |
| bl82. | ADALAT 10mg capsules |
| bl83. | ADALAT RETARD 20mg m/r tablets |
| bl84. | ADALAT RETARD 10mg m/r tablets |
| bl85. | NIFEDIPINE 5mg capsules |
| bl86. | NIFEDIPINE 10mg capsules |
| bl89. | *VASAD 5mg capsules |
| bl8A. | ADIPINE MR 20 m/r tablets |
| bl8B. | ADIPINE MR 10 m/r tablets |
| bl8C. | *UNIPINE XL 30mg m/r tablets |
| bl8D. | *NIMODREL MR 10 m/r tablets |
| bl8E. | *NIMODREL MR 20 m/r tablets |
| bl8F. | NIFEDIPINE 40mg m/r tablets |
| bl8G. | *ANGIOPINE 40 LA m/r tablets |
| bl8H. | *CARDILATE MR 10mg m/r tablets |
| bl8J. | TENSIPINE MR 10 m/r tablets |
| bl8K. | TENSIPINE MR 20 m/r tablets |
| bl8L. | FORTIPINE LA40 m/r tablets |
| bl8M. | ADALAT LA 20mg m/r tablets |
| bl8O. | *SLOFEDIPINE 20mg m/r tablets |
| bl8P. | *ANGIOPINE MR 10mg m/r tablets |
| bl8Q. | *GENALAT RETARD 10mg m/r tabs |
| bl8R. | *GENALAT RETARD 20mg m/r tabs |
| bl8S. | NIFEDIPRESS MR 10 m/r tablets |
| bl8T. | NIVATEN RETARD 10mg m/r tabs |
| bl8U. | NIFEDIPRESS MR 20 m/r tablets |
| bl8V. | NIFEDIPINE 30mg m/r capsules |
| bl8W. | NIFEDIPINE 60mg m/r capsules |
| bl8X. | CORACTEN XL 30mg m/r capsules |
| bl8Y. | CORACTEN XL 60mg m/r capsules |
| bl8Z. | NIVATEN RETARD 20mg m/r tabs |
| bl8a. | *VASAD 10mg capsules |
| bl8b. | *CALCILAT 10mg capsules |
| bl8c. | *CALCIPINE 5mg capsules |
| bl8d. | *CALCIPINE 10mg capsules |
| bl8e. | CORACTEN SR 20mg m/r capsules |
| bl8f. | *ANGIOPINE 5mg capsules |
| bl8g. | *ANGIOPINE 10mg capsules |
| bl8h. | *NIFENSAR XL 20mg m/r tablets |
| bl8i. | ADALAT LA 30mg m/r tablets |
| bl8j. | ADALAT LA 60mg m/r tablets |
| bl8k. | CORACTEN SR 10mg m/r capsules |
| bl8l. | *CARDILATE MR 20mg m/r tablets |
| bl8m. | *ANGIOPINE MR 20mg tablets |
| bl8n. | *NIFELEASE 20mg m/r tablets |
| bl8o. | *CALANIF 10mg capsules |
| bl8p. | *CALANIF 5mg capsules |
| bl8q. | *HYPOLAR RETARD 20 m/r tablets |
| bl8r. | *NIFEDOTARD 20MR m/r tablets |
| bl8s. | *CORODAY MR 20mg m/r tablets |
| bl8t. | *NIFOPRESS RETRD 20mg m/r tabs |
| bl8u. | NIFEDIPINE 10mg m/r capsules |
| bl8v. | NIFEDIPINE 20mg m/r capsules |
| bl8w. | NIFEDIPINE 10mg m/r tablets |
| bl8x. | NIFEDIPINE 30mg m/r tablets |
| bl8y. | NIFEDIPINE 60mg m/r tablets |
| bl8z. | NIFEDIPINE 20mg m/r tablets |
| bla.. | ISRADIPINE |
| bla.. | *ISRADIPINE |
| bla1. | *ISRADIPINE 2.5mg tablets |
| bla1. | ISRADIPINE 2.5mg tablets |
| bla2. | PRESCAL 2.5mg tablets |
| bla2. | *PRESCAL 2.5mg tablets |
| blb.. | AMLODIPINE |
| blb1. | AMLODIPINE 5mg tablets |
| blb2. | AMLODIPINE 10mg tablets |
| blb3. | ISTIN 5mg tablets |
| blb4. | ISTIN 10mg tablets |
| blb5. | AMLOSTIN 5mg tablets |
| blb5. | *AMLOSTIN 5mg tablets |
| blb6. | *AMLOSTIN 10mg tablets |
| blb6. | AMLOSTIN 10mg tablets |
| blb7. | AMLODIPINE 1mg/mL oral soln |
| blb8. | AMLODIPINE 2mg/mL oral soln |
| blc.. | FELODIPINE |
| blc1. | FELODIPINE 5mg m/r tablets |
| blc2. | FELODIPINE 10mg m/r tablets |
| blc3. | PLENDIL 5mg m/r tablets |
| blc4. | PLENDIL 10mg m/r tablets |
| blc5. | FELODIPINE 2.5mg m/r tablets |
| blc6. | PLENDIL 2.5mg m/r tablets |
| blc7. | CABREN 2.5mg m/r tablets |
| blc8. | CABREN 5mg m/r tablets |
| blc9. | CABREN 10mg m/r tablets |
| blca. | FELOTENS XL 5mg m/r tablets |
| blcb. | FELOTENS XL 10mg m/r tablets |
| blcc. | FELOGEN XL 5mg m/r tablets |
| blcd. | FELENDIL XL 5mg m/r tablets |
| blce. | FELENDIL XL 10mg m/r tablets |
| blcf. | KELOC SR 5mg m/r tablets |
| blcg. | KELOC SR 10mg m/r tablets |
| blch. | FELOGEN XL 10mg m/r tablets |
| blci. | VASCALPHA 5mg m/r tablets |
| blcj. | VASCALPHA 10mg m/r tablets |
| blck. | CARDIOPLEN XL 5mg m/r tablets |
| blcl. | CARDIOPLEN XL 10mg m/r tablets |
| blcm. | NEOFEL XL 5mg m/r tablets |
| blcn. | NEOFEL XL 10mg m/r tablets |
| blco. | PARMID XL 5mg m/r tablets |
| blcp. | PARMID XL 10mg m/r tablets |
| blcq. | PINEFELD XL 10mg m/r tablets |
| blcr. | CARDIOPLEN XL 2.5mg m/r tabs |
| blcs. | NEOFEL XL 2.5mg m/r tablets |
| blct. | FELOTENS XL 2.5mg m/r tablets |
| ble.. | LACIDIPINE |
| ble1. | LACIDIPINE 2mg tablets |
| ble2. | LACIDIPINE 4mg tablets |
| ble3. | MOTENS 2mg tablets |
| ble4. | MOTENS 4mg tablets |
| ble5. | MOLAP 4mg tablets |
| blg.. | NISOLDIPINE |
| blg1. | *NISOLDIPINE 10mg m/r tablets |
| blg2. | *NISOLDIPINE 20mg m/r tablets |
| blg3. | *NISOLDIPINE 30mg m/r tablets |
| blg4. | *SYSCOR MR 10mg m/r tablets |
| blg5. | *SYSCOR MR 20mg m/r tablets |
| blg6. | *SYSCOR MR 30mg m/r tablets |
| blh.. | LERCANIDIPINE HYDROCHLORIDE |
| blh1. | LERCANIDIPINE HCL 10mg tablets |
| blh2. | ZANIDIP 10mg tablets |
| blh3. | LERCANIDIPINE HCl 20mg tablets |
| blh4. | ZANIDIP 20mg tablets |
| blj.. | DILTIAZEM HYDROCHLORIDE 2 |
| blj1. | ZEMTARD 120 XL m/r capsules |
| blj2. | ZEMTARD 180 XL m/r capsules |
| blj3. | ZEMTARD 240 XL m/r capsules |
| blj4. | *OPTIL SR 90 m/r capsules |
| blj5. | *OPTIL SR 120 m/r capsules |
| blj6. | *OPTIL SR 180 m/r capsules |
| blj7. | *OPTIL XL 240 m/r capsules |
| blj8. | *OPTIL XL 300 m/r capsules |
| blj9. | *DILCARDIA XL 120mg m/r caps |
| bljA. | *DILCARDIA XL 180mg m/r caps |
| bljB. | *DILCARDIA XL 240mg m/r caps |
| bljC. | BI-CARZEM SR 60mg m/r capsules |
| bljD. | BI-CARZEM SR 90mg m/r capsules |
| bljE. | BI-CARZEM SR 120mg m/r caps |
| bljF. | *ZILDIL SR 60mg m/r capsules |
| bljG. | *ZILDIL SR 90mg m/r capsules |
| bljH. | *ZILDIL SR 120mg m/r capsules |
| bljJ. | SLOZEM 300mg m/r capsules |
| bljK. | BI-CARZEM XL 300mg m/r caps |
| bljL. | BI-CARZEM XL 240mg m/r caps |
| bljM. | ZEMRET 180 XL m/r capsules |
| bljN. | ZEMRET 240 XL m/r capsules |
| bljO. | ZEMRET 300 XL m/r capsules |
| bljP. | ADIZEM-XL 200mg m/r capsules |
| bljQ. | *DISOGRAM SR 60mg m/r capsules |
| bljR. | *DISOGRAM SR 90mg m/r capsules |
| bljS. | *DISOGRAM SR 120mg m/r caps |
| bljT. | *DISOGRAM SR 180mg m/r caps |
| bljU. | *DISOGRAM SR 240mg m/r caps |
| bljV. | *DISOGRAM SR 300mg m/r caps |
| bljW. | *HORIZEM SR 90mg m/r capsules |
| bljX. | *HORIZEM SR 120mg m/r capsules |
| bljY. | DILTIAZEM HCL XL 180mg m/r cap |
| bljZ. | DILTIAZEM HCL XL 240mg m/r cap |
| blja. | DILTIAZEM HCL XL 300mg m/r cap |
| bljb. | RETALZEM MR 60mg m/r tablets |
| bljc. | UARD 120XL m/r capsules |
| bljd. | UARD 180XL m/r capsules |
| blje. | UARD 240XL m/r capsules |
| bljf. | UARD 300XL m/r capsules |
| bll.. | NIFEDIPINE [2] |
| bll1. | SLOFEDIPINE XL 30mg m/r tabs |
| bll2. | SLOFEDIPINE XL 60mg m/r tabs |
| bll3. | *ADIPINE LA 30 m/r tablets |
| bll4. | *ADIPINE LA 60 m/r tablets |
| bll5. | HYPOLAR XL 30 m/r tablets |
| bll6. | CALCHAN MR 20mg m/r tablets |
| bll7. | CALCHAN MR 10mg m/r tablets |
| bll8. | KENTIPINE MR 10mg m/r tablets |
| bll9. | KENTIPINE MR 20mg m/r tablets |
| blla. | HYPOLAR RETARD 10mg m/r tabs |
| bllb. | VALNI 20 RETARD 20mg m/r tabs |
| bllc. | ADIPINE XL 30mg m/r tablets |
| blld. | ADIPINE XL 60mg m/r tablets |
| blle. | NIMODREL XL 60mg tablets |
| bllf. | NIMODREL XL 30mg tablets |
| bllg. | VALNI XL 30mg m/r tablets |
| bllh. | VALNI XL 60mg m/r tablets |
| blli. | NIDEF 30mg m/r tablets |
| bllj. | NIDEF 60mg m/r tablets |
| bllk. | ADANIF XL 30mg m/r tablets |
| blll. | ADANIF XL 60mg m/r tablets |
| dt1.. | NIMODIPINE |
| dt13. | NIMODIPINE 30mg tablets |
| dt14. | NIMOTOP 30mg tablets |
| bA1.. | FELODIPINE+RAMIPRIL |
| bA11. | TRIAPIN MITE 2.5mg/2.5mg tabs |
| bA12. | TRIAPIN 5mg/5mg tablets |
| bA1y. | FELODIP+RAMIPRL 2.5/2.5mg tabs |
| bA1z. | FELODIPINE+RAMIPRIL 5/5mg tabs |
| bkH.. | OLMESARTAN+AMLODIPINE |
| bkH1. | SEVIKAR 20mg/5mg tablets |
| bkH2. | SEVIKAR 40mg/5mg tablets |
| bkH3. | SEVIKAR 40mg/10mg tablets |
| bkHx. | OLMESART+AMLODIPN 40/10mg tabs |
| bkHy. | OLMESART+AMLODIPNE 40/5mg tabs |
| bkHz. | OLMESART+AMLODIPNE 20/5mg tabs |
| bkI.. | OLMESART+AMLODIP+HYDRCHLRTHIAZ |
| bkI1. | SEVIKAR HCT 20/5/12.5mg tabs |
| bkI2. | SEVIKAR HCT 40/5/12.5mg tabs |
| bkI3. | SEVIKAR HCT 40/10/12.5mg tabs |
| bkI4. | SEVIKAR HCT 40mg/5mg/25mg tabs |
| bkI5. | SEVIKAR HCT 40/10/25mg tabs |
| bd38. | *BETA-ADALAT 50/20mg capsules |
| bd39. | *TENIF 50/20mg capsules |
| bdes. | *BETA-ADALAT 50/20mg capsules |
| bdes. | BETA-ADALAT 50/20mg capsules |
| bdet. | TENIF 50/20mg capsules |
| bl5i. | *DIL+HYDROCHLOR 150/12.5mg cap |
| **Prescription of thiazide or thiazide-like diuretics** | |
| b2... | THIAZIDE DIURETICS |
| b21.. | BENDROFLUMETHIAZIDE |
| b211. | BENDROFLUMETHIAZIDE 2.5mg tabs |
| b212. | BENDROFLUMETHIAZIDE 5mg tablet |
| b213. | APRINOX 2.5mg tablets |
| b214. | APRINOX 5mg tablets |
| b215. | *BERKOZIDE 2.5mg tablets |
| b216. | *BERKOZIDE 5mg tablets |
| b217. | *CENTYL 2.5mg tablets |
| b218. | *CENTYL 5mg tablets |
| b219. | NEO-NACLEX 5mg tablets |
| b21A. | *NEO-BENDROMAX 2.5mg tablets |
| b21B. | *NEO-BENDROMAX 5mg tablets |
| b21a. | *URIZIDE 5mg tablets |
| b21b. | NEO-NACLEX 2.5mg tablets |
| b22.. | CHLOROTHIAZIDE |
| b221. | *SALURIC 500mg tablets |
| b222. | DIURIL 250mg/5mL oral susp |
| b22y. | CHLOROTHIAZIDE 250mg/5mL susp |
| b22z. | *CHLOROTHIAZIDE 500mg tablets |
| b23.. | CHLORTALIDONE |
| b231. | HYGROTON 50mg tablets |
| b232. | *HYGROTON 100mg tablets |
| b23y. | CHLORTALIDONE 50mg tablets |
| b23z. | *CHLORTHALIDONE 100mg tablets |
| b24.. | *CLOPAMIDE [INGRED see bdek] |
| b25.. | CYCLOPENTHIAZIDE |
| b251. | NAVIDREX 500micrograms tablets |
| b25z. | CYCLOPENTHIAZIDE 500mcg tabs |
| b26.. | HYDROCHLOROTHIAZIDE |
| b261. | *ESIDREX 25mg tablets |
| b262. | *ESIDREX 50mg tablets |
| b263. | *HYDROSALURIC 25mg tablets |
| b264. | *HYDROSALURIC 50mg tablets |
| b26y. | *HYDROCHLOROTHIAZIDE 50mg tabs |
| b26z. | *HYDROCHLOROTHIAZIDE 25mg tabs |
| b27.. | HYDROFLUMETHIAZIDE |
| b271. | *HYDRENOX 50mg tablets |
| b27z. | *HYDROFLUMETHIAZIDE 50mg tabs |
| b28.. | INDAPAMIDE |
| b281. | NATRILIX 2.5mg tablets |
| b282. | *NINDAXA 2.5mg tablets |
| b283. | *NATRAMID 2.5mg tablets |
| b284. | *OPUMIDE 2.5mg tablets |
| b285. | INDAPAMIDE 1.5mg m/r tablets |
| b286. | NATRILIX SR 1.5mg m/r tablets |
| b287. | ETHIBIDE XL 1.5mg m/r tablets |
| b288. | INDIPAM XL 1.5mg m/r tablets |
| b289. | MAPEMID XL 1.5mg m/r tablets |
| b28z. | INDAPAMIDE 2.5mg tablets |
| b29.. | MEFRUSIDE |
| b291. | *BAYCARON 25mg tablets |
| b29z. | *MEFRUSIDE 25mg tablets |
| b2a.. | *METHYCLOTHIAZIDE |
| b2a1. | *ENDURON 5mg tablets |
| b2az. | *METHYCLOTHIAZIDE 5mg tablets |
| b2b.. | METOLAZONE |
| b2b1. | *METENIX-5 5mg tablets |
| b2b2. | *XURET 500micrograms tablets |
| b2b3. | *METOLAZONE 500mcg tablets |
| b2bz. | *METOLAZONE 5mg tablets |
| b2c.. | POLYTHIAZIDE |
| b2c1. | *NEPHRIL 1mg tablets |
| b2cz. | *POLYTHIAZIDE 1mg tablets |
| b2d.. | XIPAMIDE |
| b2d1. | DIUREXAN 20mg tablets |
| b2dz. | XIPAMIDE 20mg tablets |
| bi1D. | *CAPTO-CO 25mg/12.5mg tablets |
| bi1E. | *CAPTO-CO 50mg/25mg tablets |
| bi1F. | *CO-ZIDOCAPT 25/12.5mg tablets |
| bi1G. | *CO-ZIDOCAPT 50mg/25mg tablets |
| bi1e. | CAPTOP+HYDROCHL 25/12.5mg tabs |
| bi1f. | CAPTOP+HYDROCHL 50/25mg tabs |
| bi1s. | CAPOZIDE 50mg/25mg tablets |
| bi28. | INNOZIDE 20/12.5mg tablets |
| bi2b. | ENALAP+HYDROCHL 20/12.5mg tabs |
| bi3n. | ZESTORETIC 20/12.5mg tablets |
| bi3p. | LISINO+HYDROCHL 20/12.5mg tabs |
| bi3s. | ZESTORETIC 10/12.5mg tablets |
| bi3t. | LISINO+HYDROCHL 10/12.5mg tabs |
| bi3u. | *CARALPHA 10/12.5mg tablets |
| bi3v. | *CARALPHA 20/12.5mg tablets |
| bi3w. | *LISICOSTAD HCT 20/12.5mg tabs |
| bi3x. | *LISICOSTAD HCT 10/12.5mg tabs |
| bi48. | QUINAPRIL+HYDROCHLOROTHIAZIDE |
| bi4F. | QUINAP+HYDROCHL 20/12.5mg tabs |
| bi55. | *PERIND ERB+INDAP 4/1.25mg tab |
| bi56. | *COVERSYL PLUS 4mg/1.25mg tabs |
| biC7. | COVRSYL ARGIN PLS 5/1.25mg tab |
| biC8. | PERIND ARG+INDAP 5/1.25mg tabs |
| bk35. | LOSART+HYDROCHLTHZ 50/12.5 tab |
| bk36. | COZAAR-COMP 50mg/12.5mg tabs |
| bk39. | COZAAR-COMP 100mg/25mg tablets |
| bk3A. | COZAAR-COMP 100mg/12.5mg tabs |
| bk3y. | LOSART+HYDRCHL 100/12.5mg tabs |
| bk3z. | LOSART+HYDROCHLTHZ 100/25 tabs |
| bk47. | CO-DIOVAN 160mg/12.5mg tablets |
| bk48. | CO-DIOVAN 160mg/25mg tablets |
| bk49. | CO-DIOVAN 80mg/12.5mg tablets |
| bk4x. | VALSART+HYDROCHL 80/12.5mg tab |
| bk4y. | VALSART+HYDROCHL 160/25mg tabs |
| bk4z. | VALSRT+HYDROCHL 160/12.5mg tab |
| bk57. | COAPROVEL 150mg/12.5mg tablets |
| bk58. | COAPROVEL 300mg/12.5mg tablets |
| bk59. | COAPROVEL 300mg/25mg tablets |
| bk5x. | IRBES+HYDROCHL 300mg/25mg tabs |
| bk5y. | IRBES+HYDROCHL 300/12.5mg tabs |
| bk5z. | IRBES+HYDROCHL 150/12.5mg tabs |
| bk86. | MICARDISPLUS 40mg/12.5mg tabs |
| bk87. | MICARDISPLUS 80mg/12.5mg tabs |
| bk88. | MICARDISPLUS 80mg/25mg tablets |
| bk8w. | TELMIS+HYDROCHL 80mg/25mg tabs |
| bk8x. | TELMIS+HYDROCHL 40/12.5mg tabs |
| bk8y. | TELMIS+HYDROCHL 80/12.5mg tabs |
| bkC.. | HYDROCHLOROTHIAZIDE+OLMESARTAN |
| bkC1. | OLMETEC PLUS 20mg/12.5mg tabs |
| bkC2. | OLMETEC PLUS 20mg/25mg tablets |
| bkC3. | OLMETEC PLUS 40mg/12.5mg tabs |
| bkCx. | OLMESAR+HYDROCH 40/12.5mg tabs |
| bkCy. | OLMESART+HYDROCHL 20/25mg tabs |
| bkCz. | OLMESAR+HYDROCH 20/12.5mg tabs |
| bkI.. | OLMESART+AMLODIP+HYDRCHLRTHIAZ |
| bkI1. | SEVIKAR HCT 20/5/12.5mg tabs |
| bkI2. | SEVIKAR HCT 40/5/12.5mg tabs |
| bkI3. | SEVIKAR HCT 40/10/12.5mg tabs |
| bkI4. | SEVIKAR HCT 40mg/5mg/25mg tabs |
| bkI5. | SEVIKAR HCT 40/10/25mg tabs |
| bdeA. | *METPROLOL+HYDROCHL 100/12.5mg |
| bdeB. | *METOPROL+HYDROCH 200/25mg m/r |
| bdeC. | *METOPROL+CHLORTHAL 100/12.5mg |
| bdeD. | *NADOLOL+BENDROFLU 40/5mg tabs |
| bdeE. | *NADOLOL+BENDROFLU 80/5mg tabs |
| bdeG. | PINDOLOL+CLOPAMIDE 10/5mg tabs |
| bdeH. | *SOTAL+HYDROCHLOR 160/25mg tab |
| bdeJ. | *SOTAL+HYDROCHLO 80/12.5mg tab |
| bdeK. | *TIMOLOL+CO-AMILOZ 10/2.5/25mg |
| bdeL. | TIMOLOL+BENDROFL 10/2.5mg tabs |
| bdeM. | *TIMOLOL+BENDROFL 20/5mg tabs |
| bdeN. | TOTARETIC 50mg/12.5mg tablets |
| bdeO. | TOTARETIC 100mg/25mg tablets |
| bdeP. | *ATENOLOL+BENDRO 25/1.25mg cap |
| bdem. | CO-TENIDONE 50/12.5mg tablets |
| bden. | CO-TENIDONE 100/25mg tablets |
| bdeo. | ATENIXCO 50/12.5mg tablets |
| bdep. | *ATENIXCO 100/25mg tablets |
| bdeq. | *TENCHLOR 50/12.5mg tablets |
| bder. | *TENCHLOR 100/25mg tablets |
| bdeu. | *CO-PRENOZIDE 160/0.25mg tabs |
| bdev. | *PROPRANOL+BENDR 80/2.5mg caps |
| bdew. | *PROPRANOL+BENDR 160/5mg m/r |
| bdex. | *ACEBU+HYDROCH 200/12.5mg tabs |
| bdey. | ATENOL+CO-AMILOZ 50/2.5/25mg |
| bl5i. | *DIL+HYDROCHLOR 150/12.5mg cap |
| **Prescription of alpha blockers** | |
| bh4.. | PRAZOSIN HYDROCHLORIDE |
| bh5y. | TERAZOSIN STARTER PACK 1mg+2mg |
| bh56. | TERAZOSIN 10mg tablets |
| bh41. | HYPOVASE 500micrograms tablets |
| bh4x. | PRAZOSIN HCL 1mg tablets |
| bh5z. | TERAZOSIN 2mg tablets |
| bh63. | DOXAZOSIN 4mg tablets |
| bh55. | TERAZOSIN 5mg tablets |
| bh54. | HYTRIN STARTER PACK tablets |
| bh6B. | DOXADURA 1mg tablets |
| bh1y. | INDORAMIN 25mg tablets |
| bh65. | CARDURA 2mg tablets |
| bh4z. | PRAZOSIN HCL 5mg tablets |
| bh14. | INDORAMIN 20mg tablets |
| bh4D. | KENTOVACE 5mg tablets |
| bh6A. | *CASCOR 4mg tablets |
| bh68. | CARDURA XL 8mg m/r tablets |
| bh4v. | *PRAZOSIN HCL STARTER PACK |
| bh4B. | KENTOVACE 1mg tablets |
| bh6F. | DOXADURA XL 4mg m/r tablets |
| bh61. | DOXAZOSIN 1mg tablets |
| bh69. | *CASCOR 2mg tablets |
| bh46. | *ALPHAVASE 500mcg tablets |
| bh21. | DIBENYLINE [CVS] 10mg capsules |
| bh6E. | SLOCINX XL 4mg m/r tablets |
| bh4y. | PRAZOSIN HCL 2mg tablets |
| bh45. | *HYPOVASE BD STARTER PACK tabs |
| bh47. | *ALPHAVASE 1mg tablets |
| bh6H. | RAPORSIN XL 4mg m/r tablets |
| bh42. | HYPOVASE 1mg tablets |
| bh6y. | DOXAZOSIN 8mg m/r tablets |
| bh1z. | *INDORAMIN 50mg tablets |
| bh5.. | TERAZOSIN HYDROCHLORIDE |
| bh6C. | DOXADURA 2mg tablets |
| bh6D. | DOXADURA 4mg tablets |
| bh57. | HYTRIN 1mg tablets |
| bh4C. | KENTOVACE 2mg tablets |
| bh4A. | KENTOVACE 500micrograms tabs |
| bh6G. | OXANDOSIN XL 4mg m/r tablets |
| bh66. | *CARDURA 4mg tablets |
| bh1.. | INDORAMIN |
| bh4w. | PRAZOSIN HCL 500mcg tablets |
| bh53. | HYTRIN 10mg tablets |
| bh44. | *HYPOVASE 5mg tablets |
| bh52. | HYTRIN 5mg tablets |
| bh43. | *HYPOVASE 2mg tablets |
| bh5x. | TERAZOSIN 1mg tablets |
| bh6z. | DOXAZOSIN 4mg m/r tablets |
| bh13. | DORALESE TILTAB 20mg tablets |
| bh51. | HYTRIN 2mg tablets |
| bh64. | CARDURA 1mg tablets |
| bh67. | CARDURA XL 4mg m/r tablets |
| bh49. | *ALPHAVASE 5mg tablets |
| bh48. | *ALPHAVASE 2mg tablets |
| bh11. | *BARATOL 25mg tablets |
| bh2y. | PHENOXYBENZAMINE 10mg capsules |
| bh6.. | DOXAZOSIN |
| bh12. | *BARATOL 50mg tablets |
| bh62. | DOXAZOSIN 2mg tablets |
| **Prescription of centrally-acting antihypertensives** | |
| bf39. | *ABICOL tablets |
| bf26. | ALDOMET 500mg tablets |
| bf1w. | CLONIDINE 100microgram tablets |
| bf1x. | *CLONIDINE 300mcg tablets |
| bf35. | *HYPERCAL 2mg tablets |
| bf... | CENTRAL ANTIHYPERTENSIVES |
| bf3b. | *RAUTRAX tablets |
| bf42. | MOXONIDINE 400micrograms tabs |
| bf44. | PHYSIOTENS 400micrograms tabs |
| bf3a. | *DECASERPYL PLUS tablets |
| bf4.. | MOXONIDINE |
| bf2.. | METHYLDOPA |
| bf2d. | MEDOMET 250mg tablets |
| bf22. | METHYLDOPA 250mg tablets |
| bf2v. | *METHYLDOPA 250mg/5mL mixture |
| bf13. | *CATAPRES PERLONGETS 250mcg |
| bf2c. | MEDOMET 250mg capsules |
| bf27. | *ALDOMET 250mg/5mL mixture |
| bf2j. | *METHYLDO+HYDROCL 250/15mg tab |
| bf23. | METHYLDOPA 500mg tablets |
| bf24. | *ALDOMET 125mg tablets |
| bf12. | *CATAPRES 300mcg tablets |
| bf3d. | *RAUWOLFIA ALKALOIDS 2mg tabs |
| bf25. | ALDOMET 250mg tablets |
| bf3c. | *SERPASIL-ESIDREX tablets |
| bf31. | *RESERPINE 100mcg tablets |
| bf43. | PHYSIOTENS 200micrograms tabs |
| bf36. | *RAUWILOID 2mg tablets |
| bf2e. | MEDOMET 500mg tablets |
| bf33. | *DECASERPYL 5mg tablets |
| bf2b. | *DOPAMET 500mg tablets |
| bf2g. | *METALPHA 250mg tablets |
| bf21. | METHYLDOPA 125mg tablets |
| bf11. | CATAPRES 100micrograms tablets |
| bf2h. | *METALPHA 500mg tablets |
| bf2z. | METHYLDOPA 250mg capsules |
| bf32. | *RESERPINE 250mcg tablets |
| bf34. | *DECASERPYL 10mg tablets |
| bf41. | MOXONIDINE 200micrograms tabs |
| bf29. | *DOPAMET 125mg tablets |
| bf2a. | *DOPAMET 250mg tablets |
| bf2f. | *HYDROMET tablets |
| bf45. | MOXONIDINE 300micrograms tabs |
| bf3.. | RESERPINE/RAUWOLFIA ALKALOIDS |
| bf37. | *SERPASIL 100microgram tablets |
| bf38. | *SERPASIL 250microgram tablets |
| bf46. | PHYSIOTENS 300micrograms tabs |
| **Prescription of vasodilators** | |
| be3x. | MINOXIDIL 2.5mg tablets |
| be2y. | HYDRALAZINE HCL 50mg tabs |
| be3.. | MINOXIDIL |
| be3z. | MINOXIDIL 10mg tablets |
| be3y. | MINOXIDIL 5mg tablets |
| be1.. | DIAZOXIDE [CVS] |
| be2x. | HYDRALAZINE HCL 25mg tabs |
| be22. | *APRESOLINE 50mg tablets |
| be32. | LONITEN 5mg tablets |
| be21. | APRESOLINE 25mg tablets |
| be31. | LONITEN 2.5mg tablets |
| be2.. | HYDRALAZINE HYDROCHLORIDE |
| be33. | LONITEN 10mg tablets |
| **Prescription of neprilysin inhibitors** | |
| bkL.. | SACUBITRIL+VALSARTAN |
| bkL1. | ENTRESTO 24mg/26mg tablets |
| bkL2. | SACUBITRL+VALSRTN 24/26mg tabs |
| bkL3. | ENTRESTO 49mg/51mg tablets |
| bkL4. | SACUBITRL+VALSRTN 49/51mg tabs |
| bkL5. | ENTRESTO 97mg/103mg tablets |
| bkL6. | SACUBTRL+VALSRTN 97/103mg tabs |
| **Prescription of oral anticoagulant** | |
| bs1.. | WARFARIN SODIUM |
| bs11. | MAREVAN 1mg tablets |
| bs12. | MAREVAN 3mg tablets |
| bs13. | MAREVAN 5mg tablets |
| bs14. | *WARFARIN WBP 1mg tablets |
| bs15. | *WARFARIN WBP 3mg tablets |
| bs16. | *WARFARIN WBP 5mg tablets |
| bs17. | WARFARIN SODIUM 1mg tablets |
| bs18. | WARFARIN SODIUM 3mg tablets |
| bs19. | WARFARIN SODIUM 5mg tablets |
| bs1A. | WARFARIN SODIUM 0.5mg tablets |
| bs1B. | MAREVAN 0.5mg tablets |
| bs1C. | WARFARIN SODIUM 1mg/1mL susp |
| bs2.. | ACENOCOUMAROL |
| bs21. | SINTHROME 1mg tablets |
| bs22. | *SINTHROME 4mg tablets |
| bs23. | ACENOCOUMAROL 1mg tablets |
| bs24. | *NICOUMALONE 4mg tablets |
| bs3.. | PHENINDIONE |
| bs31. | *DINDEVAN 10mg tablets |
| bs32. | *DINDEVAN 25mg tablets |
| bs33. | *DINDEVAN 50mg tablets |
| bs34. | PHENINDIONE 10mg tablets |
| bs35. | PHENINDIONE 25mg tablets |
| bs36. | PHENINDIONE 50mg tablets |
| bs4.. | DABIGATRAN ETEXILATE |
| bs41. | PRADAXA 75mg capsules |
| bs42. | PRADAXA 110mg capsules |
| bs43. | PRADAXA 150mg capsules |
| bs4x. | DABIGATRAN ETEXILATE 150mg cap |
| bs4y. | DABIGATRAN ETEXILATE 110mg cap |
| bs4z. | DABIGATRAN ETEXILATE 75mg caps |
| bs5.. | *DICOUMAROL [NO DRUGS HERE] |
| bs6.. | RIVAROXABAN |
| bs61. | XARELTO 10mg tablets |
| bs62. | XARELTO 15mg tablets |
| bs63. | XARELTO 20mg tablets |
| bs64. | XARELTO 2.5mg tablets |
| bs6w. | RIVAROXABAN 2.5mg tablets |
| bs6x. | RIVAROXABAN 20mg tablets |
| bs6y. | RIVAROXABAN 15mg tablets |
| bs6z. | RIVAROXABAN 10mg tablets |
| bs7.. | APIXABAN |
| bs71. | ELIQUIS 2.5mg tablets |
| bs72. | APIXABAN 2.5mg tablets |
| bs73. | ELIQUIS 5mg tablets |
| bs74. | APIXABAN 5mg tablets |
| bs8.. | EDOXABAN |
| bs81. | LIXIANA 15mg tablets |
| bs82. | LIXIANA 30mg tablets |
| bs83. | LIXIANA 60mg tablets |
| bs84. | EDOXABAN 15mg tablets |
| bs85. | EDOXABAN 30mg tablets |
| bs86. | EDOXABAN 60mg tablets |
| **Prescription of antiplatelet therapy** | |
| bu... | ANTIPLATELET DRUGS |
| bu1.. | DIPYRIDAMOLE |
| bu11. | *PERSANTIN 25mg tablets |
| bu12. | PERSANTIN 100mg tablets |
| bu13. | PERSANTIN 10mg/2mL injection |
| bu14. | DIPYRIDAMOLE 25mg tablets |
| bu15. | DIPYRIDAMOLE 100mg tablets |
| bu16. | *VASYROL 25mg tablets |
| bu17. | *VASYROL 100mg tablets |
| bu18. | *CEREBROVASE 25mg tablets |
| bu19. | *CEREBROVASE 100mg tablets |
| bu1A. | *MODAPLATE 100mg tablets |
| bu1B. | *MODAPLATE 25mg tablets |
| bu1C. | DIPYRIDAMOLE 200mg m/r caps |
| bu1D. | PERSANTIN RETARD 200mg m/r cap |
| bu1E. | DIPYRIDAMOLE 50mg/5mL susp |
| bu1F. | ATTIA 200mg m/r capsules |
| bu1G. | OFCRAM PR 200mg m/r capsules |
| bu2.. | ASPIRIN [ANTIPLATELET] |
| bu21. | *ASPIRIN 100mg eff tabs |
| bu22. | *PLATET 100mg eff tabs |
| bu23. | ASPIRIN 75mg disp tabs |
| bu24. | *ANGETTES 75mg tablets |
| bu25. | *ASPIRIN 75mg tablets |
| bu26. | *PLATET 300mg eff tabs |
| bu27. | *ASPIRIN 300mg eff tabs |
| bu28. | *DISPRIN CV 100mg m/r tablets |
| bu29. | *ASPIRIN 100mg m/r tablets |
| bu2a. | *DISPRIN CV 300mg m/r tablets |
| bu2A. | NU-SEALS ASPIRIN 75mg e/c tabs |
| bu2b. | *ASPIRIN 300mg m/r tablets |
| bu2B. | ASPIRIN 75mg e/c tablets |
| bu2C. | *POSTMI 300mg e/c tablets |
| bu2c. | ASPIRIN 75mg soluble tablets |
| bu2D. | *POSTMI 75mg dispersible tabs |
| bu2d. | FLAMASACARD 162.5mg m/r caps |
| bu2E. | *POSTMI 75mg e/c tablets |
| bu2F. | CAPRIN 75mg e/c tablets |
| bu2G. | *NU-SEALS CARDIO 75 e/c tabs |
| bu2H. | *ENPRIN 75mg e/c tablets |
| bu2I. | ASPIRIN 162.5mg m/r capsules |
| bu2J. | *CASPAC XL 162.5mg m/r caps |
| bu2K. | MICROPIRIN 75mg e/c tablets |
| bu4.. | DIPYRIDAMOLE+ASPIRIN |
| bu41. | DIPYRID+ASP 200mg/25mg m/r cap |
| bu42. | ASASANTIN RETARD m/r capsules |
| bu43. | MOLITA 200mg/25mg m/r capsules |
| bu5.. | CLOPIDOGREL |
| bu51. | CLOPIDOGREL 75mg tablets |
| bu52. | PLAVIX 75mg tablets |
| bu53. | PLAVIX 300mg tablets |
| bu54. | CLOPIDOGREL 300mg tablets |
| bu55. | GREPID 75mg tablets |
| bu6.. | TICLOPIDINE |
| bu61. | *TICLOPIDINE HCL 250mg tablets |
| bu62. | *TICLID 250mg tablets |
| bu9.. | CILOSTAZOL |
| bu91. | CILOSTAZOL 100mg tablets |
| bu92. | PLETAL 100mg tablets |
| bu93. | CILOSTAZOL 50mg tablets |
| bu94. | PLETAL 50mg tablets |
| buA.. | PRASUGREL |
| buA1. | EFIENT 5mg tablets |
| buA2. | PRASUGREL 5mg tablets |
| buA3. | EFIENT 10mg tablets |
| buA4. | PRASUGREL 10mg tablets |
| buB.. | TICAGRELOR |
| buB1. | BRILIQUE 90mg tablets |
| buBz. | TICAGRELOR 90mg tablets |
| **Prescription of non-steroidal anti-inflammatory drugs** | |
| j2... | NON-STEROIDAL ANTI-INFLAMM NOS |
| j21.. | AZAPROPAZONE |
| j211. | *RHEUMOX 300mg capsules |
| j212. | *RHEUMOX 600mg tablets |
| j21y. | *AZAPROPAZONE 300mg capsules |
| j21z. | *AZAPROPAZONE 600mg tablets |
| j22.. | DICLOFENAC SODIUM |
| j221. | *VOLTAROL 25mg tablets |
| j221. | VOLTAROL 25mg tablets |
| j222. | VOLTAROL 50mg tablets |
| j224. | VOLTAROL 100mg suppositories |
| j225. | VOLTAROL 12.5mg paed supps |
| j226. | VOLTAROL RETARD 100mg m/r tabs |
| j227. | RHUMALGAN 25mg e/c tablets |
| j228. | RHUMALGAN 50mg e/c tablets |
| j22A. | *FLAMRASE SR 100mg m/r tablets |
| j22a. | DICLOFENAC 100mg suppositories |
| j22b. | DICLOFENAC 12.5mg paed supps |
| j22B. | MOTIFENE 75mg e/c+m/r capsules |
| j22c. | DICLOFENAC 100mg m/r tablets |
| j22C. | DICLOFENAC SODIUM 75mg m/r cap |
| j22d. | DICLOFENAC 25mg e/c tablets |
| j22D. | VOLTAROL 25mg suppositories |
| j22e. | DICLOFENAC 50mg e/c tablets |
| j22E. | VOLTAROL 50mg suppositories |
| j22f. | *VOLRAMAN 25mg tablets |
| j22F. | DICLOFENAC SODIUM 25mg supps |
| j22g. | *VOLRAMAN 50mg tablets |
| j22G. | DICLOFENAC SODIUM 50mg supps |
| j22h. | *VOLTAROL 50mg disp tablets |
| j22H. | DICLOMAX SR 75mg m/r capsules |
| j22h. | VOLTAROL 50mg disp tablets |
| j22i. | DICLOFENAC 50mg disp tablets |
| j22I. | DICLOFLEX 25mg e/c tablets |
| j22j. | *VALENAC 25mg e/c tablets |
| j22J. | DICLOFLEX 50mg e/c tablets |
| j22k. | *VALENAC 50mg e/c tablets |
| j22K. | DICLOFLEX RETARD 100mg m/r tab |
| j22l. | *DICLOZIP 25mg tablets |
| j22L. | *LOFENSAID-25 tablets |
| j22M. | *LOFENSAID-50 tablets |
| j22m. | DICLOZIP 50mg tablets |
| j22N. | RHUMALGAN CR 100mg m/r tablets |
| j22n. | VOLTAROL SR 75mg m/r tablets |
| j22o. | ARTHROTEC 50 tablets |
| j22O. | RHUMALGAN CR 75mg m/r tablets |
| j22p. | DICLOF+MISOPROS 50/0.2mg tabs |
| j22P. | VOLSAID RETARD 75mg m/r tabs |
| j22q. | DICLOMAX RETARD 100mg m/r caps |
| j22Q. | VOLSAID RETARD 100mg m/r tabs |
| j22r. | DICLOFENAC 100mg m/r capsules |
| j22R. | DICLOFENAC 75mg e/c+m/r caps |
| j22s. | *ISCLOFEN 50mg e/c tablets |
| j22S. | *LOFENSAID RETARD 75 m/r tabs |
| j22T. | *LOFENSAID RETARD 100 m/r tabs |
| j22t. | DICLOF+MISOPROS 75/0.2mg tabs |
| j22u. | *DICLOFENAC SODIUM 25mg tabs |
| j22U. | *SLOFENAC SR 100mg m/r tablets |
| j22v. | *DICLOFENAC SODIUM 50mg tabs |
| j22V. | *SLOFENAC SR 75mg m/r tablets |
| j22W. | *DIGENAC XL 100 m/r tablets |
| j22w. | *FLAMRASE 25mg tablets |
| j22x. | *FLAMRASE 50mg tablets |
| j22X. | *FLEXOTARD 100mg m/r tablets |
| j22Y. | *DICLOTARD 75 m/r tablets |
| j22y. | DICLOFENAC NA 75mg m/r tabs |
| j22Z. | *DICLOTARD 100 m/r tablets |
| j22z. | ARTHROTEC 75 tablets |
| j23.. | DIFLUNISAL [MUSCULO-SKEL] |
| j231. | *DOLOBID 250mg tablets |
| j232. | *DOLOBID 500mg tablets |
| j233. | *DIFLUNISAL 250mg tablets |
| j234. | *DIFLUNISAL 500mg tablets |
| j24.. | ETODOLAC |
| j241. | *LODINE 200mg capsules |
| j242. | *RAMODAR 200mg tablets |
| j243. | *ETODOLAC 200mg capsules |
| j244. | *LODINE 300mg capsules |
| j245. | *LODINE 200mg tablets |
| j246. | *ETODOLAC 200mg tablets |
| j247. | ETODOLAC 300mg capsules |
| j248. | LODINE SR 600mg m/r tablets |
| j249. | ETODOLAC 600mg m/r tablets |
| j24A. | *EBRETIN 200mg capsules |
| j24B. | *EBRETIN 300mg capsules |
| j24C. | ECCOXOLAC 300mg capsules |
| j24D. | ETOPAN XL 600mg m/r tablets |
| j24E. | ETOLYN 600mg m/r tablets |
| j25.. | FENBUFEN |
| j251. | *LEDERFEN 300mg capsules x21 |
| j252. | *LEDERFEN CP 300mg caps x84 |
| j253. | *LEDERFEN 300mg tablets |
| j254. | *LEDERFEN CP 300mg tablets |
| j255. | *LEDERFEN 450mg tablets |
| j256. | *LEDERFEN F 450mg eff tabs 56 |
| j257. | *FENBUFEN 300mg capsules |
| j258. | *FENBUFEN 300mg tablets |
| j259. | *FENBUZIP 300mg capsules |
| j25A. | *FENBUZIP 300mg tablets |
| j25B. | *FENBUZIP 450mg tablets |
| j25y. | *FENBUFEN 450mg tablets |
| j25z. | *FENBUFEN 450mg eff tablets |
| j26.. | FENOPROFEN [MUSC SKEL] |
| j261. | FENOPRON 300mg tablets |
| j262. | *FENOPRON 600mg tablets |
| j263. | FENOPROFEN 300mg tablets |
| j264. | *FENOPROFEN 600mg tablets |
| j27.. | FLURBIPROFEN |
| j271. | *FROBEN 50mg tablets |
| j271. | FROBEN 50mg tablets |
| j272. | *FROBEN 100mg tablets |
| j272. | FROBEN 100mg tablets |
| j273. | *FROBEN 100mg suppositories |
| j274. | *FROBEN SR 200mg capsules |
| j275. | *FLURBIPROFEN 200mg m/r caps |
| j27x. | FLURBIPROFEN 50mg tablets |
| j27y. | FLURBIPROFEN 100mg tablets |
| j27z. | *FLURBIPROFEN 100mg supps |
| j28.. | IBUPROFEN [MUSC-SKEL] |
| j281. | IBUPROFEN 200mg tablets |
| j282. | IBUPROFEN 400mg tablets |
| j283. | IBUPROFEN 600mg tablets |
| j284. | *APSIFEN 200mg tablets |
| j285. | *APSIFEN 400mg tablets |
| j286. | *APSIFEN F 600mg eff tablets |
| j287. | BRUFEN 200mg tablets |
| j288. | BRUFEN 400mg tablets |
| j289. | BRUFEN 600mg tablets |
| j28a. | BRUFEN 100mg/5mL syrup |
| j28A. | BRUFEN RETARD 800mg m/r tabs |
| j28b. | EBUFAC 200mg tablets |
| j28B. | IBUPROFEN 800mg m/r tablets |
| j28C. | CUPROFEN 200mg tablets |
| j28c. | EBUFAC 400mg tablets |
| j28d. | *FENBID 300mg Spansules |
| j28D. | *ISISFEN 400mg tablets |
| j28d. | FENBID 300mg Spansules |
| j28e. | *IBULAR 200mg tablets |
| j28E. | *LIDIFEN 200mg tablets |
| j28f. | *IBULAR 400mg tablets |
| j28F. | *LIDIFEN 400mg tablets |
| j28g. | *IBUMETIN 200mg tablets |
| j28G. | LIDIFEN 600mg tablets |
| j28H. | *CODAFEN CONTINUS m/r tablets |
| j28h. | *IBUMETIN 400mg tablets |
| j28i. | *IBUMETIN 600mg tablets |
| j28I. | NUROFEN PLUS tablets |
| j28J. | *JUNIFEN 100mg/5mL s/f susp |
| j28j. | *MOTRIN 200mg tablets |
| j28K. | *IBUMED 400mg tablets |
| j28k. | *MOTRIN 400mg tablets |
| j28l. | *MOTRIN 600mg tablets |
| j28m. | *PAXOFEN 200mg tablets |
| j28M. | *RIMAFEN 200mg tablets |
| j28n. | *PAXOFEN 400mg tablets |
| j28N. | *RIMAFEN 400mg tablets |
| j28o. | *PAXOFEN 600mg tablets |
| j28O. | IBUPRO+CODEINE 200/12.5mg tabs |
| j28p. | *MOTRIN 800mg tablets |
| j28P. | IBUPROFEN 600mg/sach eff grans |
| j28q. | *ARTHROFEN 200mg tablets |
| j28R. | *INOVEN 200mg caplets |
| j28r. | ARTHROFEN 400mg tablets |
| j28s. | JUNIFEN 100mg/5mL susp 150mL |
| j28S. | RELCOFEN 200mg tablets |
| j28t. | *ARTHROFEN 600mg tablets |
| j28T. | *RELCOFEN 400mg tablets |
| j28u. | *IBUPROFEN 800mg tablets |
| j28U. | SOLPAFLEX tablets |
| j28v. | BRUFEN 600mg granules |
| j28V. | IBUPRO+CODEINE 200/12.8mg tabs |
| j28W. | *ADVIL 200mg tablets |
| j28w. | IBUPROFEN 100mg/5mL syrup |
| j28X. | *ADVIL EXTRA STRENGTH tablets |
| j28x. | IBUPROFEN 200mg m/r capsules |
| j28Y. | IBUPROFEN 100mg/5mL s/f syrup |
| j28y. | IBUPROFEN 300mg m/r capsules |
| j28z. | *IBUPR+CODEIN 300/20mg m/r tab |
| j28Z. | ANADIN IBUPROFEN 200mg tablets |
| j29.. | INDOMETACIN |
| j291. | INDOMETACIN 25mg capsules |
| j292. | INDOMETACIN 50mg capsules |
| j293. | *INDOMETACIN 100mg supps |
| j293. | INDOMETACIN 100mg supps |
| j294. | ARTRACIN 25mg capsules |
| j295. | ARTRACIN 50mg capsules |
| j296. | *IMBRILON 25mg capsules |
| j297. | *IMBRILON 50mg capsules |
| j298. | *IMBRILON 100mg suppositories |
| j299. | *INDOCID 25mg capsules |
| j29a. | *INDOCID 50mg capsules |
| j29A. | *INDOMETACIN 75mg m/r tablets |
| j29B. | *FLEXIN CONTINUS 25mg m/r tabs |
| j29b. | *INDOCID 25mg/5mL suspension |
| j29C. | *FLEXIN CONTIN LS 50mg m/r tab |
| j29c. | *INDOCID 100mg suppositories |
| j29c. | INDOCID 100mg suppositories |
| j29d. | *INDOCID-R 75mg m/r capsules |
| j29D. | *INDOMETACIN 50mg m/r tablets |
| j29e. | *INDOFLEX 25mg capsules |
| j29E. | *INDOMETACIN 25mg m/r tablets |
| j29f. | *INDOLAR 25mg capsules |
| j29F. | *RIMACID 25mg capsules |
| j29G. | *ARTRACIN SR 75mg m/r capsules |
| j29g. | *INDOLAR 50mg capsules |
| j29h. | *INDOLAR 100mg suppositories |
| j29H. | *MAXIMET SR 75mg m/r capsules |
| j29i. | INDOLAR SR 75mg m/r capsules |
| j29j. | *INDOMOD 25mg m/r capsules |
| j29J. | *INDOTARD MR 75mg m/r capsules |
| j29k. | *INDOMOD 75mg m/r capsules |
| j29K. | PARDELPRIN MR 75mg m/r caps |
| j29l. | *MOBILAN 25mg capsules |
| j29m. | *MOBILAN 50mg capsules |
| j29n. | *RHEUMACIN LA 75mg m/r caps |
| j29o. | *SLO-INDO 75mg m/r capsules |
| j29p. | *FLEXIN CONTINUS 75mg m/r tabs |
| j29r. | *INDOMAX 25mg capsules |
| j29s. | *INDOMAX 75mg m/r capsules |
| j29t. | *RHEUMACIN LA 75mg m/r caps |
| j29u. | BERLIND 75 RETARD 75mg m/r cap |
| j29w. | *INDOMETHACIN 25mg/5mL susp |
| j29y. | INDOMETACIN 75mg m/r capsules |
| j29z. | *INDOMETACIN 25mg m/r capsules |
| j2a.. | KETOPROFEN |
| j2a1. | KETOPROFEN 50mg capsules |
| j2a2. | KETOPROFEN 100mg capsules |
| j2a3. | *KETOPROFEN 100mg supps |
| j2a3. | KETOPROFEN 100mg suppositories |
| j2a4. | *ALRHEUMAT 50mg capsules |
| j2a5. | *ORUDIS 50mg capsules |
| j2a5. | ORUDIS 50mg capsules |
| j2a6. | *ORUDIS 100mg capsules |
| j2a6. | ORUDIS 100mg capsules |
| j2a7. | *ORUDIS 100mg suppositories |
| j2a7. | ORUDIS 100mg suppositories |
| j2a8. | ORUVAIL 100mg m/r capsules |
| j2a9. | ORUVAIL 200mg m/r capsules |
| j2aA. | KETOPROFEN CR 100mg m/r caps |
| j2aB. | KETOPROFEN CR 200mg m/r caps |
| j2ab. | TILOKET 50mg capsules |
| j2ac. | AXORID 100mg/20mg m/r capsules |
| j2aC. | KETOVAIL 100mg m/r capsules |
| j2ad. | AXORID 200mg/20mg m/r capsules |
| j2aD. | KETOVAIL 200mg m/r capsules |
| j2aE. | KETONAL 50mg capsules |
| j2aF. | KETONAL 100mg capsules |
| j2aG. | ORUVAIL 150mg m/r capsules |
| j2aH. | LARAFEN CR 200mg m/r capsules |
| j2aI. | *KETOCID-200 m/r capsules |
| j2aI. | KETOCID-200 m/r capsules |
| j2aJ. | *FENOKET 200mg m/r capsules |
| j2aK. | *KETOZIP CR 200mg m/r capsules |
| j2aL. | *KETOTARD 200 XL m/r capsules |
| j2aM. | *JOMETHID XL 200mg m/r caps |
| j2aO. | *KETPRON XL 100mg m/r capsules |
| j2aP. | *KETPRON XL 200mg m/r capsules |
| j2aQ. | KETIL CR 100mg m/r capsules |
| j2aR. | KETIL CR 200mg m/r capsules |
| j2aS. | VALKET RETARD 200mg m/r caps |
| j2au. | KETPRFN+OMEP 200/20mg m/r caps |
| j2av. | KETPRFN+OMEP 100/20mg m/r caps |
| j2aw. | KETOPROFEN 150mg m/r capsules |
| j2ay. | KETOPROFEN 100mg m/r capsules |
| j2az. | KETOPROFEN 200mg m/r capsules |
| j2b.. | MEFENAMIC ACID [MUSCULO-SKEL] |
| j2b1. | MEFENAMIC ACID 250mg capsules |
| j2b2. | PONSTAN 250mg capsules |
| j2b3. | PONSTAN FORTE 500mg tablets |
| j2b4. | *PONSTAN 50mg/5mL paed susp |
| j2b5. | *PONSTAN 250mg disp tablets |
| j2b6. | *DYSMAN 250mg capsules |
| j2b7. | *DYSMAN 500mg tablets |
| j2b8. | *CONTRAFLAM 250mg capsules |
| j2b9. | *MEFLAM-250 capsules |
| j2bA. | *MEFLAM-500 tablets |
| j2bB. | *OPUSTAN 250mg capsules |
| j2bC. | *OPUSTAN 500mg tablets |
| j2bD. | *CONTRAFLAM 500mg tablets |
| j2bx. | MEFENAMIC ACID 500mg tablets |
| j2by. | MEFENAMIC ACID 50mg/5mL susp |
| j2bz. | *MEFENAMIC AC 250mg disp tabs |
| j2c.. | NAPROXEN |
| j2c1. | NAPROXEN 250mg tablets |
| j2c2. | NAPROXEN 500mg tablets |
| j2c3. | *LARAFLEX 250mg tablets |
| j2c4. | *LARAFLEX 500mg tablets |
| j2c5. | NAPROSYN 250mg tablets |
| j2c6. | NAPROSYN 500mg tablets |
| j2c7. | *NAPROSYN 125mg/5mL suspension |
| j2c8. | *NAPROSYN 500mg suppositories |
| j2c9. | *NAPROSYN granules 500mg/sach |
| j2cA. | *RHEUFLEX 250mg tablets |
| j2ca. | *VALROX 250mg tablets |
| j2cB. | *RHEUFLEX 500mg tablets |
| j2cb. | *VALROX 500mg tablets |
| j2cc. | *ARTHROSIN 250mg tablets |
| j2cC. | *PRANOXEN CONT 375mg m/r tabs |
| j2cd. | *ARTHROSIN 500mg tablets |
| j2cD. | *PRANOXEN CONT 500mg m/r tabs |
| j2ce. | *ARTHROXEN 250mg tablets |
| j2cE. | *NAPROSYN S/R 500mg m/r tabs |
| j2cf. | *ARTHROXEN 500mg tablets |
| j2cF. | *TIMPRON 500mg tablets |
| j2cg. | *NAPROSYN 375mg tablets |
| j2cG. | *RIMOXYN 500mg tablets |
| j2ch. | *NAPROXEN 375mg tablets |
| j2cH. | *RIMOXYN 250mg tablets |
| j2ci. | *NYCOPREN 250mg e/c tablets |
| j2cI. | *TIMPRON 250mg tablets |
| j2cj. | *NYCOPREN 500mg e/c tablets |
| j2cJ. | *TIMPRON 250 EC e/c tablets |
| j2cK. | *TIMPRON 500 EC e/c tablets |
| j2ck. | NAPROXEN 250mg e/c tablets |
| j2cL. | *CONDROTEC tablets |
| j2cl. | NAPROXEN 500mg e/c tablets |
| j2cM. | *ARTHROSIN 250mg e/c tablets |
| j2cm. | *PROSAID 250mg tablets |
| j2cN. | *ARTHROSIN 500mg e/c tablets |
| j2cn. | *PROSAID 500mg tablets |
| j2cO. | FEMINAX ULTRA 250mg tablets |
| j2co. | NAPROSYN EC 250mg e/c tablets |
| j2cp. | NAPROSYN EC 375mg e/c tablets |
| j2cP. | VIMOVO 500mg/20mg m/r tablets |
| j2cq. | NAPROSYN EC 500mg e/c tablets |
| j2cr. | NAPRATEC 500mg/0.2mg tabs 2x56 |
| j2cs. | *NAPROXEN 375mg m/r tablets |
| j2ct. | *NAPROXEN 500mg m/r tablets |
| j2cu. | NAPROX tab+MISOPROST tab pack |
| j2cv. | *NAPROXEN granules 500mg/sach |
| j2cw. | NAPROXEN 375mg e/c tablets |
| j2cx. | *NAPROX+MISOP 500mg/200mcg tab |
| j2cy. | *NAPROXEN 125mg/5mL suspension |
| j2cz. | *NAPROXEN 500mg suppositories |
| j2cZ. | NAPROX+ESOMEP 500/20mg m/r tab |
| j2d.. | PHENYLBUTAZONE |
| j2d1. | PHENYLBUTAZONE 100mg tablets |
| j2d2. | PHENYLBUTAZONE 200mg tablets |
| j2d3. | *BUTACOTE 100mg tablets |
| j2d4. | *BUTACOTE 200mg tablets |
| j2d5. | *BUTAZOLIDIN 100mg tablets |
| j2d6. | *BUTAZOLIDIN 200mg tablets |
| j2d7. | BUTAZONE 100mg tablets |
| j2d8. | BUTAZONE 200mg tablets |
| j2e.. | PIROXICAM |
| j2e1. | PIROXICAM 10mg capsules |
| j2e2. | PIROXICAM 20mg capsules |
| j2e3. | FELDENE 10mg capsules |
| j2e4. | FELDENE 20mg capsules |
| j2e5. | *FELDENE 10mg disp tablets |
| j2e6. | *FELDENE 20mg disp tablets |
| j2e7. | *FELDENE 20mg suppositories |
| j2e8. | *LARAPAM 10mg capsules |
| j2e9. | *LARAPAM 20mg capsules |
| j2eA. | *PIROFLAM-10 capsules |
| j2ea. | *PIROZIP 10mg capsules |
| j2eB. | *PIROFLAM-20 capsules |
| j2eb. | *PIROZIP 20mg capsules |
| j2ec. | FELDENE MELT 20mg tablets |
| j2ee. | *FLAMATROL 10mg capsules |
| j2ef. | *FLAMATROL 20mg capsules |
| j2ew. | PIROXICAM 20mg melt tablets |
| j2ex. | PIROXICAM 10mg disp tablets |
| j2ey. | PIROXICAM 20mg disp tablets |
| j2ez. | *PIROXICAM 20mg suppositories |
| j2f.. | SULINDAC |
| j2f1. | *CLINORIL 100mg tablets |
| j2f2. | *CLINORIL 200mg tablets |
| j2fy. | SULINDAC 100mg tablets |
| j2fz. | SULINDAC 200mg tablets |
| j2g.. | TIAPROFENIC ACID |
| j2g1. | *SURGAM 200mg tablets |
| j2g2. | SURGAM 300mg tablets |
| j2g3. | *SURGAM granules 300mg/sachet |
| j2g4. | *SURGAM SA 300mg m/r capsules |
| j2g5. | *TIAPROFENIC AC 300mg m/r caps |
| j2gx. | *TIAPROFENIC ACID 200mg tabs |
| j2gy. | TIAPROFENIC ACID 300mg tablets |
| j2gz. | *TIAPROFEN AC grans 300mg/sach |
| j2h.. | TOLMETIN |
| j2h1. | *TOLECTIN DS 400mg capsules |
| j2h2. | *TOLECTIN 200mg capsules |
| j2h3. | *TOLECTIN 400mg capsules |
| j2hy. | *TOLMETIN 200mg capsules |
| j2hz. | *TOLMETIN 400mg capsules |
| j2i.. | PIROXICAM-BETADEX |
| j2i1. | PIROXICAM-BETADEX 191.3mg tabs |
| j2i2. | *BREXIDOL 20mg tablets |
| j2i2. | BREXIDOL 20mg tablets |
| j2j.. | ACEMETACIN |
| j2j1. | ACEMETACIN 60mg capsules |
| j2j2. | EMFLEX 60mg capsules |
| j2k.. | NABUMETONE |
| j2k1. | RELIFEX 500mg tablets |
| j2k2. | NABUMETONE 500mg tablets |
| j2k3. | *RELIFEX 500mg/5mL suspension |
| j2k3. | RELIFEX 500mg/5mL suspension |
| j2k4. | *NABUMETONE 500mg/5mL susp |
| j2k4. | NABUMETONE 500mg/5mL susp |
| j2k5. | *RELIFEX 500mg disp tabs |
| j2k6. | *NABUMETONE 500mg disp tabs |
| j2l.. | TENOXICAM |
| j2l1. | TENOXICAM 20mg tablets |
| j2l2. | MOBIFLEX 20mg tablets |
| j2l3. | *TENOXICAM 20mg/sachet grans |
| j2l4. | *MOBIFLEX 20mg/sachet granules |
| j2l5. | *TENOXICAM 20mg eff tabs |
| j2l6. | *MOBIFLEX 20mg eff tabs |
| j2m.. | ACECLOFENAC |
| j2m1. | PRESERVEX 100mg tablets |
| j2m2. | ACECLOFENAC 100mg tablets |
| j2n.. | MELOXICAM |
| j2n1. | *MELOXICAM 15mg suppositories |
| j2n2. | MELOXICAM 7.5mg tablets |
| j2n3. | MELOXICAM 15mg tablets |
| j2n4. | *MOBIC 15mg suppositories |
| j2n5. | *MOBIC 7.5mg tablets |
| j2n6. | *MOBIC 15mg tablets |
| j2n7. | *MELOXICAM 7.5mg suppositories |
| j2n8. | *MOBIC 7.5mg suppositories |
| j2n9. | MELOXICAM 7.5mg disp tablets |
| j2nA. | MELOXICAM 15mg disp tablets |
| j2o.. | DICLOFENAC SODIUM 2 |
| j2o1. | DICLOFLEX SR 75mg m/r tablets |
| j2o2. | *FLAMATAK MR 75mg m/r tablets |
| j2o3. | *DIFENOR XL 100mg m/r tablets |
| j2o4. | DICLOVOL 25mg e/c tablets |
| j2o5. | DICLOVOL 50mg e/c tablets |
| j2o6. | DICLOVOL SR 75mg m/r tablets |
| j2o7. | *DICLOVOL RETARD 100mg m/r tab |
| j2o8. | DEXOMON SR 75mg m/r tablets |
| j2o9. | DEXOMON RETARD 100mg m/r tabs |
| j2oA. | *ACOFLAM SR 75mg m/r tablets |
| j2oB. | *ACOFLAM 50mg e/c tablets |
| j2oC. | *ACOFLAM 25mg e/c tablets |
| j2oD. | *ACOFLAM RETARD 100mg m/r tabs |
| j2oE. | FLAMATAK MR 100mg m/r tablets |
| j2oF. | RHEUMATAC RETARD 75mg m/r tabs |
| j2oG. | FLAMRASE SR 75mg m/r tablets |
| j2oH. | ECONAC 100mg suppositories |
| j2oJ. | *CLOSTERIL 100 m/r tablets |
| j2ol. | DEFANAC SR 75mg m/r tablets |
| j2oL. | RHUMALGAN XL 100mg m/r caps |
| j2om. | DEFANAC RETARD 100mg m/r tabs |
| j2oM. | RHUMALGAN SR 75mg m/r capsules |
| j2oo. | *DEFANAC 25mg e/c tablets |
| j2op. | *DEFANAC 50mg e/c tablets |
| j2oP. | *VOLTAROL ACTIVE 4% spray 15mL |
| j2oP. | VOLTAROL ACTIVE 4% spray 15mL |
| j2oq. | *FENACTOL 25mg e/c tablets |
| j2oQ. | *VOLTAROL ACTIVE 4% spray 30mL |
| j2oQ. | VOLTAROL ACTIVE 4% spray 30mL |
| j2oR. | ENSTAR XL 100mg m/r tablets |
| j2or. | FENACTOL 50mg e/c tablets |
| j2os. | FENACTOL SR 75mg m/r tablets |
| j2oS. | MASIDEMEN 75mg/200mcg m/r tabs |
| j2oT. | DICLF+MISP 75mg/200mcg m/r tab |
| j2ot. | FENACTOL RETARD 100mg m/r tabs |
| j2ou. | *VALDIC 75 RETRD 75mg m/r tabs |
| j2oU. | MASIDEMEN 50mg/200mcg m/r tabs |
| j2ov. | *VALDIC 100 RETARD m/r tabs |
| j2oV. | DICLF+MISP 50mg/200mcg m/r tab |
| j2ow. | ECONAC SR 75mg m/r tablets |
| j2ox. | ECONAC XL 100mg m/r tablets |
| j2oy. | *MOBIGEL 4% spray 25g |
| j2oz. | *DICLOFENAC SODIUM 4% spray |
| j2oz. | DICLOFENAC SODIUM 4% spray |
| j2p.. | IBUPROFEN [MUSC-SKEL USE 2] |
| j2p1. | *NUROFEN ADVANCE 200mg tablets |
| j2p2. | NUROFEN FOR CHILDREN susp |
| j2p3. | NUROFEN LONG LASTING m/r caps |
| j2p4. | GALPROFEN 100mg/5mL suspension |
| j2p5. | LIBROFEM 200mg tablets |
| j2p6. | ORBIFEN FOR CHILDREN s/f susp |
| j2p7. | MANDAFEN 600mg tablets |
| j2p8. | FEVERFEN 100mg/5mL s/f susp |
| j2p9. | NUROFEN FOR CHILDN singls susp |
| j2pa. | IBUCALM 400mg tablets |
| j2pA. | IBUPROFEN 100mg/5mL sachets |
| j2pB. | MANDAFEN 400mg tablets |
| j2pb. | NUROFEN EXPRESS 400mg/sach pdr |
| j2pc. | IBUPROFEN 400mg/sachet pdr |
| j2pC. | ORBIFEN 100mg/5mL s/f susp |
| j2pD. | NUROFEN MOBILE 200mg tablets |
| j2pE. | FENPAED 100mg/5mL s/f susp |
| j2pF. | MANDAFEN 100mg/5mL s/f susp |
| j2pG. | CUPROFEN 100mg/5mL s/f susp |
| j2pH. | NUROFEN 200mg tablets |
| j2pI. | NUROFEN 200mg caplets |
| j2pJ. | IBUPROFEN 200mg capsules |
| j2pK. | NUROFEN 200mg liquid capsules |
| j2pL. | CALPROFEN 100mg/5mL s/f susp |
| j2pM. | CUPROFEN PLUS tablets |
| j2pN. | ORBIFEN 100mg/5mL s/f sachets |
| j2pO. | CARE IBUPRO 100mg/5mL s/f susp |
| j2pP. | NUROFEN FOR CHILDRN stbry susp |
| j2pQ. | GALPROFEN IBUPROF 200mg caplet |
| j2pR. | LLOYDS IBUPROFN 100mg/5mL susp |
| j2pS. | NUROFEN TENSION HEADACHE tabs |
| j2pT. | SOLPADEINE MIGRAINE IBU+COD |
| j2pU. | NUROFEN MAXIMUM 400mg Caplets |
| j2pV. | NUROFEN EXTRA 400mg caps |
| j2pW. | IBUPROFEN 400mg capsules |
| j2pX. | ANADIN ULTRA D/S 400mg caps |
| j2pY. | GALPHARM MAX IBPRFN 400mg caps |
| j2pZ. | IBUCALM 200mg tablets |
| j2q.. | DEXKETOPROFEN |
| j2q1. | KERAL 25mg tablets |
| j2qz. | DEXKETOPROFEN 25mg tablets |
| j2r.. | DICLOFENAC POTASSIUM |
| j2r1. | VOLTAROL RAPID 25mg tablets |
| j2r2. | VOLTAROL RAPID 50mg tablets |
| j2r3. | *VOLTAROL PAIN-EZE 12.5mg tabs |
| j2r3. | VOLTAROL PAIN-EZE 12.5mg tabs |
| j2rx. | *DICLOFENC POTSSIUM 12.5mg tab |
| j2rx. | DICLOFENC POTSSIUM 12.5mg tabs |
| j2ry. | DICLOFENAC POTASSIUM 25mg tabs |
| j2rz. | DICLOFENAC POTASSIUM 50mg tabs |
| j2s.. | LORNOXICAM |
| j2s1. | *XEFO 4mg tablets |
| j2s2. | *XEFO 8mg tablets |
| j2sy. | *LORNOXICAM 8mg tablets |
| j2sz. | *LORNOXICAM 4mg tablets |
| j2t.. | DEXIBUPROFEN |
| j2t1. | SERACTIL 400mg tablets |
| j2t2. | SERACTIL 300mg tablets |
| j2ty. | DEXIBUPROFEN 300mg tablets |
| j2tz. | DEXIBUPROFEN 400mg tablets |
| **Prescription of anti-arrhythmic drugs** | |
| bdc.. | SOTALOL HYDROCHLORIDE |
| bdcx. | SOTALOL 160mg tablets |
| bdcu. | SOTALOL 40mg tablets |
| bdeH. | *SOTAL+HYDROCHLOR 160/25mg tab |
| bdc4. | SOTACOR 80mg tablets |
| bdcv. | SOTALOL 80mg tablets |
| bdc3. | BETA-CARDONE 200mg tablets |
| bdeJ. | *SOTAL+HYDROCHLO 80/12.5mg tab |
| bdc1. | BETA-CARDONE 40mg tablets |
| bdcw. | SOTALOL 200mg tablets |
| bdc5. | *SOTACOR 160mg tablets |
| bdc1. | *BETA-CARDONE 40mg tablets |
| bc9x. | *QUINIDINE 300mg tablets |
| bc22. | DISOPYRAMIDE 150mg capsules |
| bcax. | *TOCAINIDE 400mg tablets |
| bc21. | DISOPYRAMIDE 100mg capsules |
| bc9.. | QUINIDINE |
| bc2.. | DISOPYRAMIDE |
| bc5x. | *MEXILETINE 200mg capsules |
| bc9w. | *QUINIDINE 200mg tablets |
| bcb4. | ARYTHMOL 300mg tablets |
| bcd4. | MORACIZINE HCL 200mg tablets |
| bc5.. | MEXILETINE HYDROCHLORIDE |
| bc25. | RYTHMODAN 150mg capsules |
| bc5z. | *MEXILETINE 360mg m/r capsules |
| bc3.. | FLECAINIDE ACETATE |
| bc2w. | *DISOPYRAMIDE 150mg m/r tabs |
| bcd.. | MORACIZINE HYDROCHLORIDE |
| bcb.. | PROPAFENONE HYDROCHLORIDE |
| bcd3. | ETHMOZINE 300mg tablets |
| bc33. | TAMBOCOR 50mg tablets |
| bc9z. | *QUINIDINE 250mg m/r tablets |
| bc3w. | FLECAINIDE ACET 200mg m/r caps |
| bc95. | *QUINICARDINE 200mg tablets |
| bc54. | *MEXITIL PL 360mg Perlongets |
| bc9y. | *QUINIDINE 250mg m/r capsules |
| bcd5. | MORACIZINE HCL 250mg tablets |
| bc1.. | BRETYLIUM TOSYLATE |
| bc2a. | DISOPYRAMIDE 250mg m/r caps |
| bc3y. | FLECAINIDE ACETATE 100mg tabs |
| bc34. | TAMBOCOR XL 200mg m/r capsules |
| bc52. | *MEXITIL 200mg capsules |
| bcb2. | PROPAFENONE 300mg tablets |
| bc81. | *PROCAINAMIDE DURULES 500mg |
| bc91. | QUINIDINE SULPHATE 200mg tabs |
| bc92. | *QUINIDINE SULPHATE 300mg tabs |
| bc94. | *KINIDIN DURULES 250mg m/r tab |
| bc3x. | FLECAINIDE ACETATE 50mg tabs |
| bc2z. | DISOPYRAMIDE 250mg m/r tablets |
| bc23. | *DIRYTHMIN SA 150mg m/r tabs |
| bc93. | *KIDITARD 250mg m/r capsules |
| bc27. | RYTHMODAN RET 250mg m/r tabs |
| bcd2. | ETHMOZINE 250mg tablets |
| bca2. | *TONOCARD 600mg tablets |
| bc91. | *QUINIDINE SULPHATE 200mg tabs |
| bcd1. | ETHMOZINE 200mg tablets |
| bc31. | TAMBOCOR 100mg tablets |
| bca1. | *TONOCARD 400mg tablets |
| bc8x. | *PROCAINAMIDE 500mg m/r tabs |
| bc51. | *MEXITIL 50mg capsules |
| bc24. | RYTHMODAN 100mg capsules |
| bcay. | *TOCAINIDE 600mg tablets |
| bca.. | TOCAINIDE HYDROCHLORIDE |
| bc5w. | *MEXILETINE 50mg capsules |
| bcb3. | ARYTHMOL 150mg tablets |
| bc8.. | PROCAINAMIDE HYDROCHLORIDE |
| bc82. | *PRONESTYL 250mg tablets |
| bc8y. | *PROCAINAMIDE HCL 250mg tabs |
| bc28. | *ISOMIDE-100 100mg capsules |
| bcb1. | PROPAFENONE 150mg tablets |
| bcd6. | MORACIZINE HCL 300mg tablets |
| bb4.. | DRONEDARONE HYDROCHLORIDE |
| bb1.. | AMIODARONE HYDROCHLORIDE |
| bb15. | *AMIDOX 200mg tablets |
| bb1y. | AMIODARONE HCL 200mg tablets |
| bb41. | MULTAQ 400mg tablets |
| bb13. | CORDARONE X 150mg/3mL inj |
| bb12. | CORDARONE X 200mg tablets |
| bb11. | CORDARONE X 100mg tablets |
| bb17. | AMYBEN 200mg tablets |
| bb16. | AMYBEN 100mg tablets |
| bb4z. | DRONEDARONE 400mg tablets |
| bb1x. | AMIODARONE HCL 100mg tablets |
| **Prescription of statins** | |
| bxd.. | SIMVASTATIN |
| bxd1. | SIMVASTATIN 10mg tablets |
| bxd2. | SIMVASTATIN 20mg tablets |
| bxd3. | ZOCOR 10mg tablets 28CP |
| bxd4. | ZOCOR 20mg tablets 28CP |
| bxd5. | SIMVASTATIN 40mg tablets |
| bxd6. | ZOCOR 40mg tablets |
| bxd7. | ZOCOR 10mg tablets |
| bxd8. | ZOCOR 20mg tablets |
| bxd9. | ZOCOR 80mg tablets |
| bxdA. | SIMVADOR 10mg tablets |
| bxdB. | SIMVADOR 20mg tablets |
| bxdC. | SIMVADOR 40mg tablets |
| bxdD. | *RANZOLONT 10mg tablets |
| bxdE. | *RANZOLONT 20mg tablets |
| bxdF. | *RANZOLONT 40mg tablets |
| bxdG. | *ZOCOR HEART-PRO 10mg tablets |
| bxdH. | INEGY 10mg/20mg tablets |
| bxdI. | INEGY 10mg/40mg tablets |
| bxdJ. | INEGY 10mg/80mg tablets |
| bxdK. | SIMVADOR 80mg tablets |
| bxdu. | SIMVASTATIN 20mg/5mL oral susp |
| bxdv. | SIMVASTATIN 40mg/5mL oral susp |
| bxdw. | SIMVAST 80mg/EZETIMIB 10mg tab |
| bxdx. | SIMVAST 40mg/EZETIMIB 10mg tab |
| bxdy. | SIMVAST 20mg/EZETIMIB 10mg tab |
| bxdz. | SIMVASTATIN 80mg tablets |
| bxe.. | PRAVASTATIN SODIUM |
| bxe1. | *PRAVASTATIN 10mg tablets |
| bxe2. | *PRAVASTATIN 20mg tablets |
| bxe3. | LIPOSTAT 10mg tablets |
| bxe4. | LIPOSTAT 20mg tablets |
| bxe5. | PRAVASTATIN SODIUM 10mg tabs |
| bxe6. | PRAVASTATIN SODIUM 20mg tabs |
| bxe7. | PRAVASTATIN SODIUM 40mg tabs |
| bxe8. | LIPOSTAT 40mg tablets |
| bxg.. | FLUVASTATIN SODIUM |
| bxg1. | LESCOL 20mg capsules |
| bxg2. | LESCOL 40mg capsules |
| bxg3. | FLUVASTATIN 20mg capsules |
| bxg4. | FLUVASTATIN 40mg capsules |
| bxg5. | LESCOL XL 80mg m/r tablets |
| bxg6. | LUVINSTA XL 80mg m/r tablets |
| bxg7. | STEFLUVIN XL 80mg m/r tablets |
| bxg8. | DORISIN XL 80mg m/r tablets |
| bxg9. | NANDOVAR XL 80mg m/r tablets |
| bxgz. | FLUVASTATIN 80mg m/r tablets |
| bxi1. | ATORVASTATIN 10mg tablets |
| bxi2. | ATORVASTATIN 20mg tablets |
| bxi3. | ATORVASTATIN 40mg tablets |
| bxi4. | LIPITOR 10mg tablets |
| bxi5. | LIPITOR 20mg tablets |
| bxi6. | LIPITOR 40mg tablets |
| bxi7. | LIPITOR 80mg tablets |
| bxiz. | ATORVASTATIN 80mg tablets |
| bxj.. | CERIVASTATIN |
| bxj1. | *CERIVASTATIN Na 100mcgs tabs |
| bxj2. | *CERIVASTATIN Na 200mcg tabs |
| bxj3. | *CERIVASTATIN Na 300mcg tabs |
| bxj4. | *LIPOBAY 100micrograms tablets |
| bxj5. | *LIPOBAY 200micrograms tablets |
| bxj6. | *LIPOBAY 300micrograms tablets |
| bxj7. | *CERIVASTATIN Na 400mcg tabs |
| bxj8. | *LIPOBAY 400micrograms tablets |
| bxj9. | *LIPOBAY 800micrograms tablets |
| bxjz. | *CERIVASTATIN Na 800mcg tabs |
| bxk1. | CRESTOR 10mg tablets |
| bxk2. | CRESTOR 20mg tablets |
| bxk3. | CRESTOR 40mg tablets |
| bxk4. | CRESTOR 5mg tablets |
| bxkw. | ROSUVASTATIN 5mg tablets |
| bxkx. | ROSUVASTATIN 10mg tablets |
| bxky. | ROSUVASTATIN 20mg tablets |
| bxkz. | ROSUVASTATIN 40mg tablets |
| **Prescription of cardiac glycosides** | |
| b11D. | *DIGAMEX 125micrograms tablets |
| b113. | DIGOXIN 250microgram tablets |
| b14z. | *MEDIGOXIN 100mcg tablets |
| b123. | *DIGITALINE NATIVELLE 1mg/mL |
| b11A. | DIGOXIN 50microgram/mL elixir |
| b13.. | *LANATOSIDE C |
| b131. | *CEDILANID 250mcg tablets |
| b11.. | DIGOXIN |
| b118. | LANOXIN-PG 62.5mcg tablets |
| b13z. | *LANATOSIDE C 250mcg tablets |
| b116. | LANOXIN 250micrograms tablets |
| b119. | LANOXIN-PG 50mcg/mL elixir |
| b141. | *LANITOP 100microgram tablets |
| b115. | LANOXIN 125micrograms tablets |
| b122. | *DIGITALINE NATIVELLE 100mcg |
| b11C. | *DIGAMEX 62.5mcg tablets |
| b121. | *DIGITOXIN 100microgram tabs |
| b11E. | *DIGAMEX 250micrograms tablets |
| b1... | CARDIAC GLYCOSIDES |
| b151. | *OUABAINE ARNAUD 250mcg/1mL |
| b112. | DIGOXIN 125microgram tablets |
| b12.. | DIGITOXIN |
| b111. | DIGOXIN 62.5micrograms tablets |
| **Prescription of rate-limiting calcium channel blockers** | |
| bb3.. | VERAPAMIL HYDROCHLORIDE |
| bb31. | VERAPAMIL 40mg tablets |
| bb32. | VERAPAMIL 80mg tablets |
| bb33. | VERAPAMIL 120mg tablets |
| bb34. | *BERKATENS 40mg tablets |
| bb35. | *BERKATENS 80mg tablets |
| bb36. | *BERKATENS 120mg tablets |
| bb37. | *BERKATENS 160mg tablets |
| bb38. | *CORDILOX 40mg tablets |
| bb39. | *CORDILOX 80mg tablets |
| bb3A. | VERAPAMIL 240mg m/r tablets |
| bb3B. | HALF SECURON SR 120mg m/r tabs |
| bb3C. | VERAPAMIL 120mg m/r tablets |
| bb3D. | VERAPAMIL 40mg/5mL s/f soln |
| bb3F. | HALF-SECURON SR 120mg 28CP |
| bb3G. | *HYPANEZE 40 tablets |
| bb3H. | *HYPANEZE 80 tablets |
| bb3J. | *HYPANEZE 120 tablets |
| bb3K. | *VERAPRESS MR 240 m/r tablets |
| bb3L. | *ETHIMIL MR 240 m/r tablets |
| bb3M. | CORDILOX MR 240 m/r tablets |
| bb3N. | ZOLVERA 40mg/5mL oral solution |
| bb3O. | *RANVERA MR 240mg m/r tablets |
| bb3P. | VERA-TIL SR 240mg m/r tablets |
| bb3Q. | VERA-TIL SR 120mg m/r tablets |
| bb3a. | CORDILOX 120mg tablets |
| bb3b. | *CORDILOX 160mg tablets |
| bb3d. | *SECURON 40mg tablets |
| bb3e. | *SECURON 80mg tablets |
| bb3f. | *SECURON 120mg tablets |
| bb3g. | *SECURON 120mg tablets 56CP |
| bb3h. | *SECURON 160mg tablets 56CP |
| bb3i. | *SECURON 160mg tablets |
| bb3j. | SECURON SR 240mg m/r tablets |
| bb3k. | SECURON SR 240mg m/r tabs 28CP |
| bb3l. | UNIVER 120mg m/r capsules x28 |
| bb3m. | UNIVER 180mg m/r capsules x56 |
| bb3n. | UNIVER 240mg m/r capsules x28 |
| bb3p. | *GEANGIN 40mg tablets |
| bb3q. | *GEANGIN 80mg tablets |
| bb3r. | *GEANGIN 120mg tablets |
| bb3s. | VERTAB SR 240 m/r tablets |
| bb3v. | VERAPAMIL 120mg m/r capsules |
| bb3w. | VERAPAMIL 160mg tablets |
| bb3x. | *VERPAMIL HCL 120mg tabs x56 |
| bb3y. | VERAPAMIL 240mg m/r capsules |
| bb3z. | VERAPAMIL 180mg m/r capsules |
| bl5.. | DILTIAZEM HYDROCHLORIDE |
| bl51. | TILDIEM 60mg tablets |
| bl52. | *CALCICARD 60mg tablets |
| bl53. | *BRITIAZIM 60mg tablets |
| bl54. | ADIZEM-SR 120mg m/r tablets |
| bl55. | DILTIAZEM HCL 120mg m/r tabs |
| bl56. | *ANGIOZEM 60mg tablets |
| bl57. | *ADIZEM 60mg tablets |
| bl58. | TILDIEM RETARD 90mg m/r tabs |
| bl59. | TILDIEM RETARD 120mg m/r tabs |
| bl5A. | TILDIEM LA 300mg m/r capsules |
| bl5B. | ADIZEM-SR 90mg m/r capsules |
| bl5C. | ADIZEM-SR 120mg m/r capsules |
| bl5D. | ADIZEM-SR 180mg m/r capsules |
| bl5E. | ADIZEM-XL 300mg m/r capsules |
| bl5F. | DILZEM SR 60mg m/r capsules |
| bl5G. | DILZEM SR 90mg m/r capsules |
| bl5H. | DILZEM SR 120mg m/r capsules |
| bl5I. | ADIZEM-XL 240mg m/r capsules |
| bl5J. | ADIZEM-XL 180mg m/r capsules |
| bl5K. | ADIZEM-XL 120mg m/r capsules |
| bl5L. | DILZEM-XL 120mg m/r capsules |
| bl5M. | DILZEM-XL 180mg m/r capsules |
| bl5N. | DILZEM-XL 240mg m/r capsules |
| bl5O. | SLOZEM 120mg m/r capsules |
| bl5P. | SLOZEM 180mg m/r capsules |
| bl5Q. | SLOZEM 240mg m/r capsules |
| bl5R. | ANGITIL SR 90 m/r capsules |
| bl5S. | ANGITIL SR 120 m/r capsules |
| bl5T. | *METAZEM 60mg tablets |
| bl5U. | ANGITIL SR 180 m/r capsules |
| bl5V. | *CALCICARD CR 90mg m/r tablets |
| bl5V. | CALCICARD CR 90mg m/r tablets |
| bl5W. | CALCICARD CR 120mg m/r tablets |
| bl5W. | *CALCICARD CR 120mg m/r tabs |
| bl5X. | KENTIAZEM 60mg m/r capsules |
| bl5Y. | *OPTIL 60mg m/r tablets |
| bl5Z. | TILDIEM LA 200mg m/r capsules |
| bl5a. | DILTIAZEM HCL 90mg m/r tablets |
| bl5b. | DILTIAZEM HCL 300mg m/r caps |
| bl5c. | DILTIAZEM HCL 90mg m/r caps |
| bl5d. | DILTIAZEM HCL 120mg m/r caps |
| bl5e. | DILTIAZEM HCL 180mg m/r caps |
| bl5f. | DILTIAZEM HCL 60mg m/r caps |
| bl5g. | DILTIAZEM HCL 240mg m/r caps |
| bl5h. | DILTIAZEM HCL 200mg m/r caps |
| bl5j. | *ADIZEM-XL PLUS m/r capsules |
| bl5k. | *ANGIOZEM CR 90mg m/r tablets |
| bl5l. | DILCARDIA SR 60mg m/r capsules |
| bl5m. | *ANGIOZEM CR 120mg m/r tablets |
| bl5n. | ZEMTARD 300 XL m/r capsules |
| bl5o. | VIAZEM XL 120mg m/r capsules |
| bl5p. | VIAZEM XL 180mg m/r capsules |
| bl5q. | VIAZEM XL 240mg m/r capsules |
| bl5r. | VIAZEM XL 300mg m/r capsules |
| bl5s. | DILTIAZEM HCL 360mg m/r caps |
| bl5t. | VIAZEM XL 360mg m/r capsules |
| bl5u. | *CALAZEM 60mg m/r tablets |
| bl5v. | DILCARDIA SR 90mg m/r capsules |
| bl5w. | DILCARDIA SR 120mg m/r caps |
| bl5x. | ANGITIL XL 240 m/r capsules |
| bl5y. | ANGITIL XL 300 m/r capsules |
| bl5z. | DILTIAZEM HCL 60mg m/r tablets |
| blj.. | DILTIAZEM HYDROCHLORIDE 2 |
| blj1. | ZEMTARD 120 XL m/r capsules |
| blj2. | ZEMTARD 180 XL m/r capsules |
| blj3. | ZEMTARD 240 XL m/r capsules |
| blj4. | *OPTIL SR 90 m/r capsules |
| blj5. | *OPTIL SR 120 m/r capsules |
| blj6. | *OPTIL SR 180 m/r capsules |
| blj7. | *OPTIL XL 240 m/r capsules |
| blj8. | *OPTIL XL 300 m/r capsules |
| blj9. | *DILCARDIA XL 120mg m/r caps |
| bljA. | *DILCARDIA XL 180mg m/r caps |
| bljB. | *DILCARDIA XL 240mg m/r caps |
| bljC. | BI-CARZEM SR 60mg m/r capsules |
| bljD. | BI-CARZEM SR 90mg m/r capsules |
| bljE. | BI-CARZEM SR 120mg m/r caps |
| bljF. | *ZILDIL SR 60mg m/r capsules |
| bljG. | *ZILDIL SR 90mg m/r capsules |
| bljH. | *ZILDIL SR 120mg m/r capsules |
| bljJ. | SLOZEM 300mg m/r capsules |
| bljK. | BI-CARZEM XL 300mg m/r caps |
| bljL. | BI-CARZEM XL 240mg m/r caps |
| bljM. | ZEMRET 180 XL m/r capsules |
| bljN. | ZEMRET 240 XL m/r capsules |
| bljO. | ZEMRET 300 XL m/r capsules |
| bljP. | ADIZEM-XL 200mg m/r capsules |
| bljQ. | *DISOGRAM SR 60mg m/r capsules |
| bljR. | *DISOGRAM SR 90mg m/r capsules |
| bljS. | *DISOGRAM SR 120mg m/r caps |
| bljT. | *DISOGRAM SR 180mg m/r caps |
| bljU. | *DISOGRAM SR 240mg m/r caps |
| bljV. | *DISOGRAM SR 300mg m/r caps |
| bljW. | *HORIZEM SR 90mg m/r capsules |
| bljX. | *HORIZEM SR 120mg m/r capsules |
| bljY. | DILTIAZEM HCL XL 180mg m/r cap |
| bljZ. | DILTIAZEM HCL XL 240mg m/r cap |
| blja. | DILTIAZEM HCL XL 300mg m/r cap |
| bljb. | RETALZEM MR 60mg m/r tablets |
| bljc. | UARD 120XL m/r capsules |
| bljd. | UARD 180XL m/r capsules |
| blje. | UARD 240XL m/r capsules |
| bljf. | UARD 300XL m/r capsules |
| bl5i. | *DIL+HYDROCHLOR 150/12.5mg cap |

# Supplementary Table 3. The RECORD statement – checklist of items, extended from the STROBE statement that should be reported in observational studies using routinely collected health data.

|  | **Item No.** | **STROBE items** | **Location in manuscript where items are reported** | **RECORD items** | **Location in manuscript where items are reported** |
| --- | --- | --- | --- | --- | --- |
| **Title and abstract** | | | | | |
|  | 1 | (a) Indicate the study’s design with a commonly used term in the title or the abstract (b) Provide in the abstract an informative and balanced summary of what was done and what was found | Page 2 | RECORD 1.1: The type of data used should be specified in the title or abstract. When possible, the name of the databases used should be included.  RECORD 1.2: If applicable, the geographic region and timeframe within which the study took place should be reported in the title or abstract.  RECORD 1.3: If linkage between databases was conducted for the study, this should be clearly stated in the title or abstract. | Page 1 & 2 |
| **Introduction** | | | | | |
| Background rationale | 2 | Explain the scientific background and rationale for the investigation being reported | Page 4 |  |  |
| Objectives | 3 | State specific objectives, including any prespecified hypotheses | Page 5 |  |  |
| **Methods** | | | | | |
| Study Design | 4 | Present key elements of study design early in the paper | Pages 5 & 6 |  |  |
| Setting | 5 | Describe the setting, locations, and relevant dates, including periods of recruitment, exposure, follow-up, and data collection | Pages 5 & 6 |  |  |
| Participants | 6 | *(a) Cohort study* - Give the eligibility criteria, and the sources and methods of selection of participants. Describe methods of follow-up  *Case-control study* - Give the eligibility criteria, and the sources and methods of case ascertainment and control selection. Give the rationale for the choice of cases and controls  *Cross-sectional study* - Give the eligibility criteria, and the sources and methods of selection of participants  *(b) Cohort study* - For matched studies, give matching criteria and number of exposed and unexposed  *Case-control study* - For matched studies, give matching criteria and the number of controls per case | Page 5 | RECORD 6.1: The methods of study population selection (such as codes or algorithms used to identify subjects) should be listed in detail. If this is not possible, an explanation should be provided.  RECORD 6.2: Any validation studies of the codes or algorithms used to select the population should be referenced. If validation was conducted for this study and not published elsewhere, detailed methods and results should be provided.  RECORD 6.3: If the study involved linkage of databases, consider use of a flow diagram or other graphical display to demonstrate the data linkage process, including the number of individuals with linked data at each stage. | Supplementary Tables 1 & 2 |
| Variables | 7 | Clearly define all outcomes, exposures, predictors, potential confounders, and effect modifiers. Give diagnostic criteria, if applicable. | Page 6 | RECORD 7.1: A complete list of codes and algorithms used to classify exposures, outcomes, confounders, and effect modifiers should be provided. If these cannot be reported, an explanation should be provided. | Figure 1,  Supplementary Tables 1, 2 & 4 |
| Data sources/ measurement | 8 | For each variable of interest, give sources of data and details of methods of assessment (measurement).  Describe comparability of assessment methods if there is more than one group | Page 5 |  |  |
| Bias | 9 | Describe any efforts to address potential sources of bias | Page 5 |  |  |
| Study size | 10 | Explain how the study size was arrived at | Page 5 |  |  |
| Quantitative variables | 11 | Explain how quantitative variables were handled in the analyses. If applicable, describe which groupings were chosen, and why | Pages 5-7 |  |  |
| Statistical methods | 12 | (a) Describe all statistical methods, including those used to control for confounding  (b) Describe any methods used to examine subgroups and interactions  (c) Explain how missing data were addressed  (d) *Cohort study* - If applicable, explain how loss to follow-up was addressed  *Case-control study* - If applicable, explain how matching of cases and controls was addressed  *Cross-sectional study* - If applicable, describe analytical methods taking account of sampling strategy  (e) Describe any sensitivity analyses | Page 7 |  |  |
| Data access and cleaning methods |  | .. |  | RECORD 12.1: Authors should describe the extent to which the investigators had access to the database population used to create the study population.  RECORD 12.2: Authors should provide information on the data cleaning methods used in the study. | Page 5 |
| Linkage |  | .. |  | RECORD 12.3: State whether the study included person-level, institutional-level, or other data linkage across two or more databases. The methods of linkage and methods of linkage quality evaluation should be provided. | Pages 5 & 6 |
| **Results** | | | | | |
| Participants | 13 | (a) Report the numbers of individuals at each stage of the study (*e.g.*, numbers potentially eligible, examined for eligibility, confirmed eligible, included in the study, completing follow-up, and analysed)  (b) Give reasons for non-participation at each stage.  (c) Consider use of a flow diagram | Page 8 | RECORD 13.1: Describe in detail the selection of the persons included in the study (*i.e.,* study population selection) including filtering based on data quality, data availability and linkage. The selection of included persons can be described in the text and/or by means of the study flow diagram. | Page 8 |
| Descriptive data | 14 | (a) Give characteristics of study participants (*e.g.*, demographic, clinical, social) and information on exposures and potential confounders  (b) Indicate the number of participants with missing data for each variable of interest  (c) *Cohort study* - summarise follow-up time (*e.g.*, average and total amount) | Supplementary Figure 1, Table 1 |  |  |
| Outcome data | 15 | *Cohort study* - Report numbers of outcome events or summary measures over time  *Case-control study* - Report numbers in each exposure category, or summary measures of exposure  *Cross-sectional study* - Report numbers of outcome events or summary measures | Pages 9 & 10, Tables 2 & 3, Supplementary Tables 5-10 |  |  |
| Main results | 16 | (a) Give unadjusted estimates and, if applicable, confounder-adjusted estimates and their precision (e.g., 95% confidence interval). Make clear which confounders were adjusted for and why they were included  (b) Report category boundaries when continuous variables were categorized  (c) If relevant, consider translating estimates of relative risk into absolute risk for a meaningful time period | Pages 9 & 10, Tables 2 & 3 |  |  |
| Other analyses | 17 | Report other analyses done—e.g., analyses of subgroups and interactions, and sensitivity analyses | Pages 9 & 10, Supplementary Tables 5-10 |  |  |
| **Discussion** | | | | | |
| Key results | 18 | Summarise key results with reference to study objectives | Page 10 |  |  |
| Limitations | 19 | Discuss limitations of the study, taking into account sources of potential bias or imprecision. Discuss both direction and magnitude of any potential bias | Pages 10-12 | RECORD 19.1: Discuss the implications of using data that were not created or collected to answer the specific research question(s). Include discussion of misclassification bias, unmeasured confounding, missing data, and changing eligibility over time, as they pertain to the study being reported. | Page 15 |
| Interpretation | 20 | Give a cautious overall interpretation of results considering objectives, limitations, multiplicity of analyses, results from similar studies, and other relevant evidence | Pages 10-12 |  |  |
| Generalisability | 21 | Discuss the generalisability (external validity) of the study results | Pages 10-13 |  |  |
| **Other Information** | | | | | |
| Funding | 22 | Give the source of funding and the role of the funders for the present study and, if applicable, for the original study on which the present article is based | Page 15 |  |  |
| Accessibility of protocol, raw data, and programming code |  | .. |  | RECORD 22.1: Authors should provide information on how to access any supplemental information such as the study protocol, raw data, or programming code. | Supplementary Tables 1, 2 & 4 |

*Reference: Benchimol EI, Smeeth L, Guttmann A, Harron K, Moher D, Petersen I, Sørensen HT, von Elm E, Langan SM, the RECORD Working Committee. The REporting of studies Conducted using Observational Routinely-collected health Data (RECORD) Statement. *PLoS Medicine* 2015; in press.

*Checklist is protected under Creative Commons Attribution ([CC BY](http://creativecommons.org/licenses/by/4.0/)) license.

Supplementary Figure 1. Adherence to the ABC pathway and its components.

**ABC** n=5,531 (38.2%)

**BC**

n=4,854 (33.5%)

**AB** n=1,041

(7.2%)

**AC** n=433 (3.0%)

# Supplementary Table 4. Components of CHA_2_DS2-VASc and HAS-BLED risk assessment scores adjusted for in sensitivity analyses.

| Variables adjusted for in sensitivity analyses | Variable type | Component of HAS-BLED^c^ or CHA_2_DS_2_-VASc score |
| --- | --- | --- |
| Age | Continuous | HAS-BLED and CHA_2_DS_2_-VASc score |
| Gender | Categorical | CHA_2_DS_2_-VASc |
| Heart failure^a^ | Categorical | CHA_2_DS_2_-VASc |
| Hypertension^a^ | Categorical | CHA_2_DS_2_-VASc |
| Stroke/transient ischaemic attack/thromboembolism^a^ | Categorical | CHA_2_DS_2_-VASc |
| Diabetes^a^ | Categorical | CHA_2_DS_2_-VASc |
| Vascular disease (myocardial infarction, peripheral vascular disease or aortic plaque)^a^ | Categorical | CHA_2_DS_2_-VASc |
| Liver disease^a^ | Categorical | HAS-BLED |
| Renal disease^a^ | Categorical | HAS-BLED |
| Bleeding^a^ | Categorical | HAS-BLED |
| Antiplatelet or non-steroidal anti-inflammatory drug^b^ | Categorical | HAS-BLED |
| Harmful alcohol consumption^a^ | Categorical | HAS-BLED |

^a^diagnoses prior to care home entry

^b^prescription within 6 months prior to care home entry

^c^‘uncontrolled hypertension’ and ‘stroke’ components of HAS-BLED not adjusted for because of overlap with ‘hypertension’ and ‘stroke/transient ischaemic attack/thromboembolism’ components of CHA_2_DS_2_-VASc

# Supplementary Table 5. Assessment of multicollinearity between covariates (including CHA_2_DS_2_VASc and HAS-BLED scores), using the Variance Inflation Factor.

| **Covariate included in multivariate model** | **Variance Inflation Factor** |
| --- | --- |
| Age (continuous variable) | 1.08 |
| Gender | 1.26 |
| Welsh Index of Multiple Deprivation | 1.00 |
| Electronic Frailty Index (continuous variable) | 1.32 |
| CHA_2_DS_2_VASc score (continuous variable) | 1.48 |
| HAS-BLED score (continuous variable) | 1.42 |
| Smoking history | 1.10 |
| Dementia^a^ | 1.01 |
| Pulmonary disease^b^ | 1.07 |
| Cancer | 1.02 |
| Peptic ulcer disease | 1.01 |

^a^including Alzheimer’s disease, vascular dementia, younger onset dementia and other or unspecified dementia

^b^including asthma, chronic obstructive pulmonary disease and other pulmonary disease

# Supplementary Table 6. Assessment of multicollinearity between covariates (including individual components that constitute CHA_2_DS_2_VASc and HAS-BLED scores^a^), using the Variance Inflation Factor.

| **Covariate included in multivariate model** | **Variance Inflation Factor** |
| --- | --- |
| Age (continuous variable) | 1.10 |
| Gender | 1.09 |
| Welsh Index of Multiple Deprivation | 1.01 |
| Electronic Frailty Index (continuous variable) | 1.62 |
| Liver disease | 1.01 |
| Renal disease | 1.03 |
| Bleeding^b^ | 1.05 |
| Prescription of antiplatelet or non-steroid anti-inflammatory drug(s)^c^ | 1.31 |
| Harmful alcohol use^d^ | 1.04 |
| Heart failure | 1.09 |
| Hypertension | 1.13 |
| Diabetes | 1.05 |
| Stroke/transient ischaemic attack/thromboembolism | 1.03 |
| Vascular disease^e^ | 1.03 |
| Smoking history | 1.11 |
| Dementia^f^ | 1.01 |
| Pulmonary disease^g^ | 1.07 |
| Cancer | 1.02 |
| Peptic ulcer disease | 1.02 |

**^a^**two components of the HAS-BLED score (stroke and uncontrolled hypertension) not adjusted for because hypertension and stroke/transient ischaemic attack/thromboembolism already accounted for as part of the CHA_2_DS_2_VASc score

^b^including haemorrhagic stroke or major bleeding

^c^prescription within six months prior to care home entry

^d^including alcoholism and heavy drinker

^e^including peripheral vascular disease, aortic plaque and myocardial infarction

^f^including Alzheimer’s disease, vascular dementia, younger onset dementia and other or unspecified dementia

^g^including asthma, chronic obstructive pulmonary disease and other pulmonary disease

# Supplementary Table 7. Risk of the composite outcome, stroke, transient ischaemic attack, cardiovascular hospitalisation, major bleeding and mortality in care home residents aged ≥65 years by partial ABC adherence^a^ on care home entry (2003-2018) – Cox Regression Analysis.

|  | **Unadjusted Hazard Ratio (95% CI), p-value** | **Adjusted Hazard Ratio^b^ (95% CI), p-value** | **Adjusted Hazard Ratio^c^ (95% CI), p-value** |
| --- | --- | --- | --- |
| **Composite** | | | |
| 0 or 1 ABC criteria fulfilled | 1 | 1 | 1 |
| 2 ABC criteria fulfilled | 0.97 (0.92 to 1.02), p=0.182 | **0.95 (0.90 to 1.00), p=0.037** | 0.99 (0.94 to 1.04), p=0.716 |
| 3 ABC criteria fulfilled | 1.01 (0.96 to 1.06), p=0.826 | 0.97 (0.92 to 1.03), p=0.292 | 1.01 (0.96 to 1.07), p=0.655 |
| **Ischaemic stroke** | | | |
| 0 or 1 ABC criteria fulfilled | 1 | 1 | 1 |
| 2 ABC criteria fulfilled | 0.97 (0.74 to 1.27), p=0.826 | 0.95 (0.72 to 1.25), p=0.716 | 0.96 (0.73 to 1.27), p=0.782 |
| 3 ABC criteria fulfilled | 1.06 (0.80 to 1.39), p=0.700 | 1.12 (0.83 to 1.50), p=0.471 | 1.13 (0.84 to 1.52), p=0.420 |
| **Haemorrhagic stroke** | | | |
| 0 or 1 ABC criteria fulfilled | 1 | 1 | 1 |
| 2 ABC criteria fulfilled | 1.20 (0.67 to 2.16), p=0.538 | 1.31 (0.71 to 2.42), p=0.382 | 1.29 (0.70 to 2.40), p=0.412 |
| 3 ABC criteria fulfilled | **1.79 (1.01 to 3.19), p=0.046** | **1.95 (1.05 to 3.65), p=0.035** | **1.92 (1.03 to 3.59), p=0.041** |
| **Stroke of unknown origin** | | | |
| 0 or 1 ABC criteria fulfilled | 1 | 1 | 1 |
| 2 ABC criteria fulfilled | 0.79 (0.50 to 1.25), p=0.322 | 0.73 (0.45 to 1.18), p=0.199 | 0.69 (0.43 to 1.13), p=0.139 |
| 3 ABC criteria fulfilled | 1.01 (0.64 to 1.60), p=0.964 | 0.83 (0.50 to 1.37), p=0.463 | 0.81 (0.49 to 1.33), p=0.405 |
| **Transient Ischaemic Attack** | | | |
| 0 or 1 ABC criteria fulfilled | 1 | 1 | 1 |
| 2 ABC criteria fulfilled | 0.96 (0.59 to 1.57), p=0.879 | 0.90 (0.54 to 1.50), p=0.699 | 0.85 (0.51 to 1.41), p=0.524 |
| 3 ABC criteria fulfilled | 1.04 (0.63 to 1.72), p=0.878 | 1.04 (0.60 to 1.78), p=0.895 | 0.99 (0.58 to 1.69), p=0.971 |
| **Cardiovascular hospitalisation** | | | |
| 0 or 1 ABC criteria fulfilled | 1 | 1 | 1 |
| 2 ABC criteria fulfilled | **0.89 (0.82 to 0.98), p=0.016** | **0.87 (0.79 to 0.96), p=0.005** | 0.95 (0.87 to 1.05), p=0.340 |
| 3 ABC criteria fulfilled | 1.00 (0.91 to 1.10), p=0.945 | 0.91 (0.82 to 1.01), p=0.064 | 1.00 (0.90 to 1.10), p=0.925 |
| **Major bleeding** | | | |
| 0 or 1 ABC criteria fulfilled | 1 | 1 | 1 |
| 2 ABC criteria fulfilled | 1.02 (0.82 to 1.27), p=0.831 | 1.11 (0.88 to 1.39), p=0.369 | 1.05 (0.83 to 1.31), p=0.705 |
| 3 ABC criteria fulfilled | 1.18 (0.94 to 1.48), p=0.142 | 1.25 (0.98 to 1.59), p=0.073 | 1.21 (0.95 to 1.54), p=0.122 |
| **Myocardial infarction** | | | |
| 0 or 1 ABC criteria fulfilled | 1 | 1 | 1 |
| 2 ABC criteria fulfilled | 1.05 (0.73 to 1.52), p=0.781 | 1.02 (0.69 to 1.49), p=0.937 | 1.02 (0.69 to 1.50), p=0.928 |
| 3 ABC criteria fulfilled | 0.72 (0.48 to 1.09), p=1.124 | 0.71 (0.46 to 1.11), p=0.133 | 0.73 (0.47 to 1.14), p=0.163 |
| **All-cause mortality** | | | |
| 0 or 1 ABC criteria fulfilled | 1 | 1 | 1 |
| 2 ABC criteria fulfilled | 0.97 (0.93 to 1.02), p=0.270 | 0.96 (0.91 to 1.01), p=0.081 | 0.99 (0.94 to 1.05), p=0.799 |
| 3 ABC criteria fulfilled | 1.00 (0.95 to 1.05), p=0.852 | 0.98 (0.93 to 1.04), p=0.561 | 1.02 (0.97 to 1.08), p=0.479 |
| **Cardiovascular mortality** | | | |
| 0 or 1 ABC criteria fulfilled | 1 | 1 | 1 |
| 2 ABC criteria fulfilled | 0.99 (0.84 to 1.16), p=0.899 | 1.00 (0.84 to 1.18), p=0.960 | 1.04 (0.88 to 1.23), p=0.675 |
| 3 ABC criteria fulfilled | 0.92 (0.78 to 1.09), p=0.340 | 0.92 (0.77 to 1.10), p=0.369 | 0.96 (0.80 to 1.15), p=0.638 |

ABC, Atrial fibrillation Better Care; AF, atrial fibrillation; CI, confidence interval

Significant results in **bold**

^a^n=2,634 adherent to 0 or 1 ABC criteria, n=6,328 adherent to 2 ABC criteria, n=5,531 adherent to 3 ABC criteria

^b^hazard ratio adjusted for age, sex, Welsh Index of Multiple Deprivation, electronic Frailty Index, smoking, dementia, pulmonary disease, cancer, peptic ulcer disease, CHA_2_DS_2_VASc and HAS-BLED risk assessment scores

^c^hazard ratio adjusted for age, sex, Welsh Index of Multiple Deprivation, electronic Frailty Index, smoking, dementia, pulmonary disease, cancer, peptic ulcer disease and individual components that constitute CHA_2_DS_2_VASc and HAS-BLED risk assessment scores

# Supplementary Table 8. Risk of stroke, transient ischaemic attack, cardiovascular hospitalisation, major bleeding and mortality in care home residents aged ≥65 years by partial ABC adherence^a^ on care home entry (2003-2018) – Competing Risk Analysis.

|  | **Unadjusted sub-distribution hazard ratio (95% CI), p-value** | **Adjusted sub-distribution hazard ratio^b^ (95% CI), p-value** | **Adjusted sub-distribution hazard ratio^c^ (95% CI), p-value** |
| --- | --- | --- | --- |
| **Ischaemic stroke** | | | |
| 0 or 1 ABC criteria fulfilled | 1 | 1 | 1 |
| 2 ABC criteria fulfilled | 0.98 (0.75 to 1.27), p=0.858 | 0.98 (0.74 to 1.30), p=0.899 | 0.96 (0.72 to 1.27), p=0.766 |
| 3 ABC criteria fulfilled | 0.99 (0.75 to 1.30), p=0.926 | 1.07 (0.79 to 1.46), p=0.659 | 1.04 (0.77 to 1.42), p=0.782 |
| **Haemorrhagic stroke** | | | |
| 0 or 1 ABC criteria fulfilled | 1 | 1 | 1 |
| 2 ABC criteria fulfilled | 1.21 (0.67 to 2.18), p=0.526 | 1.34 (0.74 to 2.44), p=0.337 | 1.27 (0.69 to 2.33), p=0.436 |
| 3 ABC criteria fulfilled | 1.72 (0.97 to 3.07), p=0.065 | **1.93 (1.05 to 3.54), p=0.034** | 1.81 (1.00 to 3.29), p=0.05 |
| **Stroke of unknown origin** | | | |
| 0 or 1 ABC criteria fulfilled | 1 | 1 | 1 |
| 2 ABC criteria fulfilled | 0.80 (0.50 to 1.26), p=0.335 | 0.74 (0.45 to 1.22), p=0.237 | 0.68 (0.42 to 1.12), p=0.129 |
| 3 ABC criteria fulfilled | 0.96 (0.61 to 1.52), p=0.860 | 0.80 (0.46 to 1.37), p=0.411 | 0.76 (0.45 to 1.28), p=0.307 |
| **Transient Ischaemic Attack** | | | |
| 0 or 1 ABC criteria fulfilled | 1 | 1 | 1 |
| 2 ABC criteria fulfilled | 0.97 (0.60 to 1.59) p=0.918 | 0.93 (0.55 to 1.59), p=0.791 | 0.84 (0.49 to 1.44), p=0.523 |
| 3 ABC criteria fulfilled | 0.98 (0.59 to 1.63), p=0.948 | 1.00 (0.56 to 1.76), p=0.990 | 0.92 (0.53 to 1.61), p=0.776 |
| **Cardiovascular hospitalisation** | | | |
| 0 or 1 ABC criteria fulfilled | 1 | 1 | 1 |
| 2 ABC criteria fulfilled | 0.89 (0.81 to 0.97), p=0.012 | **0.88 (0.80 to 0.97), p=0.007** | 0.94 (0.85 to 1.03), p=0.173 |
| 3 ABC criteria fulfilled | 0.95 (0.87 to 1.04), p=0.294 | **0.87 (0.79 to 0.97), p=0.009** | 0.93 (0.84 to 1.03), p=0.185 |
| **Major bleeding** | | | |
| 0 or 1 ABC criteria fulfilled | 1 | 1 | 1 |
| 2 ABC criteria fulfilled | 1.03 (0.82 to 1.28), p=0.820 | 1.13 (0.90 to 1.42), p=0.296 | 1.04 (0.83 to 1.31), p=0.726 |
| 3 ABC criteria fulfilled | 1.11 (0.89 to 1.38), p=0.371 | 1.19 (0.93 to 1.53), p=0.160 | 1.13 (0.89 to 1.44), p=0.321 |
| **Myocardial infarction** | | | |
| 0 or 1 ABC criteria fulfilled | 1 | 1 | 1 |
| 2 ABC criteria fulfilled | 1.06 (0.73 to 1.53), p=0.757 | 1.04 (0.70 to 1.53), p=0.859 | 1.01 (0.68 to 1.50), p=0.946 |
| 3 ABC criteria fulfilled | 0.68 (0.45 to 1.02), p=0.065 | 0.68 (0.43 to 1.06), p=0.087 | 0.68 (0.44 to 1.06), p=0.089 |
| **All-cause mortality**^d^ | | | |
| 0 or 1 ABC criteria fulfilled | 1 | 1 | 1 |
| 2 ABC criteria fulfilled | 0.97 (0.93 to 1.02), p=0.270 | 0.96 (0.91 to 1.01), p=0.081 | 0.99 (0.94 to 1.05), p=0.799 |
| 3 ABC criteria fulfilled | 1.00 (0.95 to 1.05), p=0.852 | 0.98 (0.93 to 1.04), p=0.561 | 1.02 (0.97 to 1.08), p=0.479 |
| **Cardiovascular mortality**^d^ | | | |
| 0 or 1 ABC criteria fulfilled | 1 | 1 | 1 |
| 2 ABC criteria fulfilled | 0.99 (0.84 to 1.16), p=0.899 | 1.00 (0.84 to 1.18), p=0.960 | 1.04 (0.88 to 1.23), p=0.675 |
| 3 ABC criteria fulfilled | 0.92 (0.78 to 1.09), p=0.340 | 0.92 (0.77 to 1.10), p=0.369 | 0.96 (0.80 to 1.15), p=0.638 |

ABC, Atrial fibrillation Better Care; AF, atrial fibrillation; CI, confidence interval

Significant results in **bold**

^a^n=2,634 adherent to 0 or 1 ABC criteria, n=6,328 adherent to 2 ABC criteria, n=5,531 adherent to 3 ABC criteria

^b^main analysis – sub-distribution hazard ratio adjusted for age, sex, Welsh Index of Multiple Deprivation, electronic Frailty Index, smoking, dementia, pulmonary disease, cancer, peptic ulcer disease, CHA_2_DS_2_VASc and HAS-BLED risk assessment scores

^c^sensitivity analysis – sub-distribution hazard ratio adjusted for age, sex, Welsh Index of Multiple Deprivation, electronic Frailty Index, smoking, dementia, pulmonary disease, cancer, peptic ulcer disease and individual components that constitute CHA_2_DS_2_VASc and HAS-BLED risk assessment scores

^d^hazard ratio not sub-distributed, standard Cox regression analysis

# Supplementary Table 9. Risk of the composite outcome, stroke, transient ischaemic attack, cardiovascular hospitalisation, major bleeding and mortality in care home residents aged ≥65 years by partial ABC adherence^a^ on care home entry (2003-2018) – Cox Regression Analysis.

|  | **Unadjusted Hazard Ratio (95% CI), p-value** | **Adjusted Hazard Ratio^b^ (95% CI), p-value** | **Adjusted Hazard Ratio^c^ (95% CI), p-value** |
| --- | --- | --- | --- |
| **Composite** | | | |
| AB | **1.09 (1.02 to 1.17), p=0.009** | **1.09 (1.02 to 1.17), p=0.011** | 1.05 (0.98 to 1.13), p=0.140 |
| AC | **0.86 (0.78 to 0.95), p=0.004** | **0.84 (0.76 to 0.93), p=0.001** | **0.86 (0.77 to 0.95), p=0.003** |
| BC | **0.96 (0.92 to 0.99), p=0.021** | **0.96 (0.93 to 1.00), p=0.045** | 0.99 (0.95 to 1.03), p=0.540 |
| **Ischaemic stroke** | | | |
| AB | 1.11 (0.76 to 1.62), p=0.598 | 1.09 (0.74 to 1.59), p=0.662 | 1.11 (0.76 to 1.63), p=0.580 |
| AC | 0.97 (0.58 to 1.62), p=0.907 | 0.99 (0.59 to 1.67), p=0.979 | 0.99 (0.58 to 1.66), p=0.955 |
| BC | 0.91 (0.74 to 1.12), p=0.368 | 0.85 (0.69 to 1.06), p=0.148 | 0.85 (0.68 to 1.06), p=0.143 |
| **Haemorrhagic stroke** | | | |
| AB | 0.97 (0.45 to 2.09), p=0.939 | 0.94 (0.44 to 2.04), p=0.883 | 1.07 (0.49 to 2.32), p=0.862 |
| AC | 1.55 (0.68 to 3.53), p=0.298 | 1.70 (0.74 to 3.93), p=0.211 | 1.69 (0.73 to 3.91), p=0.217 |
| BC | 0.71 (0.46 to 1.08), p=0.107 | 0.73 (0.47 to 1.13), p=0.155 | 0.70 (0.45 to 1.08), p=0.103 |
| **Stroke of unknown origin** | | | |
| AB | 0.78 (0.36 to 1.67), p=0.521 | 0.83 (0.39 to 1.78), p=0.635 | 0.84 (0.39 to 1.81), p=0.656 |
| AC | 1.25 (0.55 to 2.83), p=0.598 | 1.11 (0.49 to 2.55), p=0.800 | 1.11 (0.49 to 2.54), p=0.803 |
| BC | 0.79 (0.54 to 1.14), p=0.201 | 0.83 (0.56 to 1.21), p=0.329 | 0.79 (0.54 to 1.17), p=0.241 |
| **Transient Ischaemic Attack** | | | |
| AB | 0.37 (0.12 to 1.15), p=0.086 | 0.37 (0.12 to 1.17), p=0.091 | 0.38 (0.12 to 1.19), p=0.096 |
| AC | 0.88 (0.32 to 2.38), p=0.796 | 0.79 (0.29 to 2.15), p=0.640 | 0.78 (0.29 to 2.15), p=0.636 |
| BC | 1.13 (0.78 to 1.64), p=0.504 | 1.09 (0.74 to 1.59), p=0.667 | 1.05 (0.71 to 1.54), p=0.823 |
| **Cardiovascular hospitalisation** | | | |
| AB | 0.98 (0.85 to 1.12), p=0.750 | 1.00 (0.87 to 1.14), p=0.947 | 0.94 (0.82 to 1.08), p=0.368 |
| AC | **1.49 (1.28 to 1.73), p<0.001** | **1.40 (1.19 to 1.63), p<0.001** | **1.45 (1.24 to 1.70), p<0.001** |
| BC | **0.82 (0.76 to 0.89), p<0.001** | **0.86 (0.80 to 0.93), p<0.001** | **0.90 (0.83 to 0.97), p=0.007** |
| **Major bleeding** | | | |
| AB | 0.99 (0.72 to 1.36), p=0.967 | 0.92 (0.67 to 1.27), p=0.609 | 1.01 (0.73 to 1.39), p=0.945 |
| AC | 1.31 (0.91 to 1.88), p=0.145 | 1.39 (0.96 to 2.00), p=0.080 | 1.33 (0.92 to 1.91), p=0.131 |
| BC | 0.87 (0.73 to 1.02), p=0.091 | 0.92 (0.78 to 1.09), p=0.351 | 0.86 (0.72 to 1.02), p=0.089 |
| **Myocardial infarction** | | | |
| AB | 1.30 (0.78 to 2.17), p=0.317 | 1.31 (0.78 to 2.19), p=0.306 | 1.20 (0.71 to 2.02), p=0.496 |
| AC | 1.71 (0.95 to 3.07), p=0.072 | 1.76 (0.97 to 3.19), p=0.063 | 1.81 (1.00 to 3.29), p=0.050 |
| BC | 1.09 (0.81 to 1.45), p=0.578 | 1.04 (0.77 to 1.41), p=0.799 | 1.05 (0.77 to 1.43), p=0.761 |
| **All-cause mortality** | | | |
| AB | **1.12 (1.05 to 1.20), p=0.001** | **1.12 (1.04 to 1.20), p=0.002** | **1.08 (1.01 to 1.16), p=0.028** |
| AC | **0.78 (0.71 to 0.87), p<0.001** | **0.78 (0.70 to 0.87), p<0.001** | **0.79 (0.71 to 0.88), p<0.001** |
| BC | 0.98 (0.94 to 1.01), p=0.203 | 0.97 (0.93 to 1.01), p=0.088 | 0.99 (0.95 to 1.03), p=0.659 |
| **Cardiovascular mortality** | | | |
| AB | 1.15 (0.91 to 1.44), p=0.237 | 1.12 (0.89 to 1.41), p=0.331 | 1.10 (0.87 to 1.38), p=0.424 |
| AC | 1.02 (0.75 to 1.39), p=0.884 | 1.03 (0.75 to 1.40), p=0.870 | 1.04 (0.76 to 1.42), p=0.820 |
| BC | 1.00 (0.89 to 1.14), p=0.961 | 1.02 (0.89 to 1.15), p=0.819 | 1.04 (0.91 to 1.18), p=0.581 |

ABC, Atrial fibrillation Better Care; AF, atrial fibrillation; CI, confidence interval

Significant results in **bold**

^a^n=1,041 adherent to AB criteria, n=433 adherent to AC criteria, n=4,854 adherent to BC criteria

^b^hazard ratio adjusted for age, sex, Welsh Index of Multiple Deprivation, electronic Frailty Index, smoking, dementia, pulmonary disease, cancer, peptic ulcer disease, CHA_2_DS_2_VASc and HAS-BLED risk assessment scores

^c^hazard ratio adjusted for age, sex, Welsh Index of Multiple Deprivation, electronic Frailty Index, smoking, dementia, pulmonary disease, cancer, peptic ulcer disease and individual components that constitute CHA_2_DS_2_VASc and HAS-BLED risk assessment scores

# Supplementary Table 10. Incidence and risk of stroke, transient ischaemic attack, cardiovascular hospitalisation, major bleeding and mortality in care home residents aged ≥65 years by ABC status on care home entry (2003-2018) – Competing Risk Analysis.

|  | **Unadjusted sub-distribution hazard ratio (95% CI), p-value** | **Adjusted sub-distribution hazard ratio^a^ (95% CI), p-value** | **Adjusted sub-distribution hazard ratio^b^ (95% CI), p-value** |
| --- | --- | --- | --- |
| **Ischaemic stroke** | | | |
| AB | 1.01 (0.69 to 1.47), p=0.960 | 0.99 (0.68 to 1.45), p=0.979 | 1.05 (0.72 to 1.54), p=0.796 |
| AC | 1.20 (0.72 to 2.00), p=0.494 | 1.24 (0.74 to 2.10), p=0.416 | 1.21 (0.72 to 2.04), p=0.473 |
| BC | 0.95 (0.78 to 1.17), p=0.660 | 0.90 (0.73 to 1.12), p=0.363 | 0.88 (0.71 to 1.10, p=0.266 |
| **Haemorrhagic stroke** | | | |
| AB | 0.90 (0.42 to 1.94), p=0.790 | 0.88 (0.41 to 1.89), p=0.745 | 1.01 (0.47 to 2.20), p=0.971 |
| AC | 1.84 (0.81 to 4.18), p=0.147 | 2.04 (0.89 to 4.66), p=0.092 | 1.98 (0.86 to 4.54), p=0.108 |
| BC | 0.73 (0.48 to 1.11), p=0.144 | 0.75 (0.49 to 1.16), p=0.202 | 0.71 (0.46 to 1.10), p=0.124 |
| **Stroke of unknown origin** | | | |
| AB | 0.72 (0.34 to 1.55), p=0.405 | 0.77 (0.36 to 1.66), p=0.511 | 0.79 (0.37 to 1.72), p=0.561 |
| AC | 1.48 (0.65 to 3.35), p=0.348 | 1.32 (0.56 to 3.11), p=0.524 | 1.30 (0.56 to 3.06), p=0.542 |
| BC | 0.82 (0.56 to 1.18), p=0.287 | 0.86 (0.57 to 1.28), p=0.449 | 0.81 (0.54 to 1.21), p=0.300 |
| **Transient Ischaemic Attack** | | | |
| AB | 0.34 (0.11 to 1.06), p=0.062 | 0.35 (0.11 to 1.09), p=0.069 | 0.36 (0.12 to 1.14), p=0.083 |
| AC | 1.08 (0.40 to 2.93), p=0.877 | 0.96 (0.35 to 2.64), p=0.936 | 0.92 (0.34 to 2.52), p=0.872 |
| BC | 1.19 (0.82 to 1.72), p=0.360 | 1.14 (0.78 to 1.66), p=0.501 | 1.07 (0.73 to 1.57), p=0.735 |
| **Cardiovascular hospitalisation** | | | |
| AB | 0.90 (0.79 to 1.04), p=0.148 | 0.92 (0.80 to 1.06), p=0.255 | 0.89 (0.78 to 1.02), p=0.100 |
| AC | **1.86 (1.60 to 2.16), p<0.001** | **1.76 (1.51 to 2.05), p<0.001** | **1.81 (1.55 to 2.11), p<0.001** |
| BC | **0.84 (0.78 to 0.91), p<0.001** | **0.88 (0.82 to 0.95), p=0.001** | **0.91 (0.84 to 0.98), p=0.013** |
| **Major bleeding** | | | |
| AB | 0.91 (0.66 to 1.24), p=0.540 | 0.85 (0.62 to 1.17), p=0.316 | 0.95 (0.69 to 1.31), p=0.747 |
| AC | **1.63 (1.13 to 2.33), p=0.008** | **1.75 (1.22 to 2.51), p=0.003** | **1.65 (1.15 to 2.38), p=0.007** |
| BC | 0.90 (0.76 to 1.07), p=0.233 | 0.96 (0.81 to 1.14), p=0.643 | 0.89 (0.74 to 1.05), p=0.170 |
| **Myocardial infarction** | | | |
| AB | 1.19 (0.72 to 1.99), p=0.497 | 1.21 (0.72 to 2.02), p=0.469 | 1.15 (0.67 to 1.95), p=0.612 |
| AC | 2.09 (1.17 to 3.76), p=0.013 | 2.19 (1.25 to 3.90), p=0.008 | 2.23 (1.25 to 3.98), p=0.007 |
| BC | 1.14 (0.85 to 1.52), p=0.387 | 1.09 (0.80 to 1.49), p=0.573 | 1.08 (0.79 to 1.48), p=0.631 |
| **All-cause mortality**^c^ | | | |
| AB | **1.12 (1.05 to 1.20), p=0.001** | **1.12 (1.04 to 1.20), p=0.002** | **1.08 (1.01 to 1.16), p=0.028** |
| AC | **0.78 (0.71 to 0.87), p<0.001** | **0.78 (0.70 to 0.87), p<0.001** | **0.79 (0.71 to 0.88), p<0.001** |
| BC | 0.98 (0.94 to 1.01), p=0.203 | 0.97 (0.93 to 1.01), p=0.088 | 0.99 (0.95 to 1.03), p=0.659 |
| **Cardiovascular mortality**^c^ | | | |
| AB | 1.15 (0.91 to 1.44), p=0.237 | 1.12 (0.89 to 1.41), p=0.331 | 1.10 (0.87 to 1.38), p=0.424 |
| AC | 1.02 (0.75 to 1.39), p=0.884 | 1.03 (0.75 to 1.40), p=0.870 | 1.04 (0.76 to 1.42), p=0.820 |
| BC | 1.00 (0.89 to 1.14), p=0.961 | 1.02 (0.89 to 1.15), p=0.819 | 1.04 (0.91 to 1.18), p=0.581 |

ABC, Atrial fibrillation Better Care; AF, atrial fibrillation; CI, confidence interval

Significant results in **bold**

^a^main analysis – sub-distribution hazard ratio adjusted for age, sex, Welsh Index of Multiple Deprivation, electronic Frailty Index, smoking, dementia, pulmonary disease, cancer, peptic ulcer disease, CHA_2_DS_2_VASc and HAS-BLED risk assessment scores

^b^sensitivity analysis – sub-distribution hazard ratio adjusted for age, sex, Welsh Index of Multiple Deprivation, electronic Frailty Index, smoking, dementia, pulmonary disease, cancer, peptic ulcer disease and individual components that constitute CHA_2_DS_2_VASc and HAS-BLED risk assessment scores

^c^hazard ratio not sub-distributed, standard Cox regression analysis
